# A Nanoparticle‐Integrated Complete Manufacturing Pipeline of Chemically Engineered Exosomes

**DOI:** 10.1002/advs.202516075

**Published:** 2026-03-24

**Authors:** Xiaowei Wen, Zixing Xu, Zerun Hao, Yanming Chen, Kai Xie, Haofan Yin, Xueying Wang, Jie Min, Sihan Sun, Baiding Chen, Chengxiu Ling, Mingming Xu, Yizhao Chen, Gang Ruan

**Affiliations:** ^1^ Wisdom Lake Academy of Pharmacy Xi'an Jiaotong‐Liverpool University Suzhou China; ^2^ Jiangsu Province Higher Education Key Laboratory of Cell Therapy Nanoformulation (Construction) Xi'an Jiaotong‐Liverpool University Suzhou China; ^3^ Suzhou Industrial Park Center of Excellence for Single‐Molecule and Single‐Cell Analysis Xi'an Jiaotong‐Liverpool University Suzhou China; ^4^ Suzhou Key Laboratory of Chest Disease Diagnosis and Treatment The Fourth Affiliated Hospital of Soochow University Suzhou China; ^5^ Department of Neurosurgery The Seventh Affiliated Hospital Southern Medical University Foshan China

**Keywords:** cell therapy, extracellular vesicle, nanomedicine, production, separation

## Abstract

Clinical translation of engineered exosomes, an emerging class of cell therapies, is hampered by challenges in each step of the manufacture flow, namely biogenesis, cargo loading, isolation, and storage. Here, we present a technology termed Tat‐PNCAS‐MIMS‐MSC‐Exo for manufacturing chemically engineered exosomes, with the above four steps being integrated by the use of the nanoparticle PNCAS‐Tat (Tat peptide‐conjugated protein‐nanoparticle co‐assembly supraparticle). This technology enables drastic improvements in all four steps of the manufacture flow of chemically engineered exosomes derived from mesenchymal stem cells (MSCs), a commonly‐used cell type for cell therapies. The stimulation effect of exosome biogenesis by Tat peptide can be amplified by nanoparticle conjugation, a previously unknown nano‐effect. The novel design of magnet setup MIMS (mobile internal magnetic separation) enables a unique capacity for scale‐up of magnetic isolation, i.e., near‐identical time for different scales to achieve near‐complete isolation. This offers an effective solution to the long‐standing problem of scale‐up in applying magnetic isolation for biomanufacturing, which usually requires larger scales than bioanalytical applications. The manufacture process is robust, scalable, and economical. We conduct mechanistic studies of the nano‐bio interactions, and demonstrate applications of the products in multiple disease models.

## Graphical abstract

We report a novel manufacture technology of chemically engineered exosomes. The four steps of manufacturing, i.e., biogenesis, loading, isolation, and storage, are integrated by the use of a nanoparticle. The manufacture technology incorporates three innovative components, i.e., a new nano‐bio effect, a new composite nanoparticle, and a new isolation instrumentation design. The performances of all four steps are greatly enhanced.

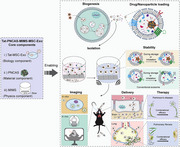

## 1 Introduction

Extracellular vesicles (EVs) such as exosomes have demonstrated great potential as an emerging class of cell therapies as well as nanomedicines [1, 2, 3, 4, 5, 6]. Compared with other types of EVs, e.g., microvesicles and apoptotic bodies, exosomes (common size range 50–150 nm in diameter) are believed to be favorable for therapy because the biogenesis of exosomes involves a distinct intracellular regulatory process which likely dictates their composition and functions, thereby yielding better‐controlled therapeutic outcomes [1, 3, 7, 8]. Mesenchymal stem cells (MSCs) are one of the most commonly used cell types to produce exosomes for therapeutics, especially for regeneration and immunomodulation [3, 5, 9, 10, 11, 12]. MSCs‐derived exosomes have been examined in many diseases and disorders, such as Alzheimer's disease, brain injury, chronic kidney disease, retinal ischemia‐reperfusion, corneal injury, diabetes, and COVID‐19, in preclinical studies as well as in clinical trials [3, 5, 9, 10, 11, 12]. The therapeutic efficacies of exosomes themselves are often not sufficiently powerful to yield clinical importance; thus ‘engineered exosomes’ have been introduced to offer enhanced or/and extra functionalities, by combining with drugs, nucleic acids, nanomaterials, or hydrogels [1, 3, 5, 13, 14, 15, 16, 17, 18]. In general, there are two kinds of engineered exosomes depending on the approaches to modify the exosome structures, namely chemically engineered exosomes (i.e., exosomes with chemicals/materials added) and genetically engineered exosomes (i.e., exosomes with genetic modifications). Currently, clinical translation of engineered exosomes faces major hurdles in the manufacturing aspect, including a lack of quantity and purity, difficulty in loading cargos with high quantity and efficiency, low efficiency, poor scalability, and the need of expensive instrument of isolation, low stability during storage, and high overall cost [1, 3, 4, 5, 6]. To make improvement in the manufacturing aspect of engineered exosomes, a number of techniques have been developed, such as starvation treatment on the source cells to stimulate their exosome generation, applying tangential flow filtration for exosome isolation to improve the scalability of isolation, employing mechanical treatment on exosomes for transiently opening the exosome membranes to increase loading of cargos, using cryoprotectants to reduce stability loss of exosomes during a freeze‐thaw cycle in lyophilization‐based storage, among others [3, 4, 5, 6, 19, 20, 21, 22]. However, typically each of these techniques can only improve one (or, in rare cases, two) [22] of the four steps of the manufacturing flow of engineered exosomes, i.e., biogenesis, loading of cargos, isolation, and storage [3, 4, 5, 6, 19, 20, 21, 22].

We envision an idea of an integrated platform technology that could improve all the above four steps of the manufacturing flow of chemically engineered exosomes. The idea involves simply incubating the source cell with a composite nanoparticle that is loaded with both magnetic nanoparticles and drugs and is coated with exosome biogenesis‐stimulating ligands. By doing so, the quantity of exosomes generated from each source cell is expected to be increased by the biogenesis‐stimulating ligand; loading of the cargos (drugs and nanoparticles) into exosomes is expected to be accomplished at the same time as the exosome biogenesis, utilizing the natural cellular uptake process of composite nanoparticles; the thus‐formed chemically engineered exosomes (exosomes loaded with drugs and nanoparticles) are expected to be isolated with a magnet, harnessing the magnetism of magnetic nanoparticles loaded in the exosomes; the storage stability of the engineered exosomes is expected to have an improvement compared to that of native exosomes, due to the presence of structurally stable nanoparticles (the magnetic nanoparticles) in the exosomes.

However, to make this idea practical and drastically superior to simple combinations of existing techniques, there are several critical problems to be solved with regard to achieving the required biological, nanomaterial, and magnetic attributes, respectively: (1) It is not enough just to stimulate the exosome biogenesis of source cells. Also required are all of the following: high endocytosis‐mediated uptake of composite nanoparticles into the source cells, a substantial percentage of the endocytosed composite nanoparticles being secreted into the extracellular environment, and a dominant majority of the objects collected by magnetic isolation from the extracellular environment being composite nanoparticles‐encapsulated exosomes (i.e., no or few free composite nanoparticles). It is unclear whether all of these are achievable. (2) The composite nanoparticles need to have high loading for both magnetic nanoparticles and drugs; and the size of the composite nanoparticles needs to be small enough to fit into exosomes (50–150 nm in diameter). But the composite nanoparticles reported in the literature rarely offer all of these three features [23, 24, 25, 26, 27, 28]. (3) With the conventional setup of a magnet, i.e., placing a magnet on the exterior surface of a container of exosome dispersion [29, 30]. the time durations needed for complete magnetic isolation of the composite nanoparticles‐encapsulated exosomes will be prohibitively long for industrial operation. This is because the diameters of the composite nanoparticles (<150 nm) are about one order of magnitude smaller than those of the magnetic beads typically used for magnetic isolation of exosomes (1–10 µm) [29, 30]. Since the magnetic force is proportional to magnetic volume (proportional to diameter to the third power) [31], the time duration for magnetic isolation of exosomes will be ∼1000 times longer for the composite nanoparticles than that for the typical magnetic beads. Although using some alternative magnet setups previously reported, e.g., microfluidic magnetic device [32] and quadruple magnetic flow sorter [31, 33], might help to reduce the time duration needed, these setups are primarily suitable for analytical rather than therapeutic applications as they suffer from low scalability and sometimes high cost.

**FIGURE 1:**
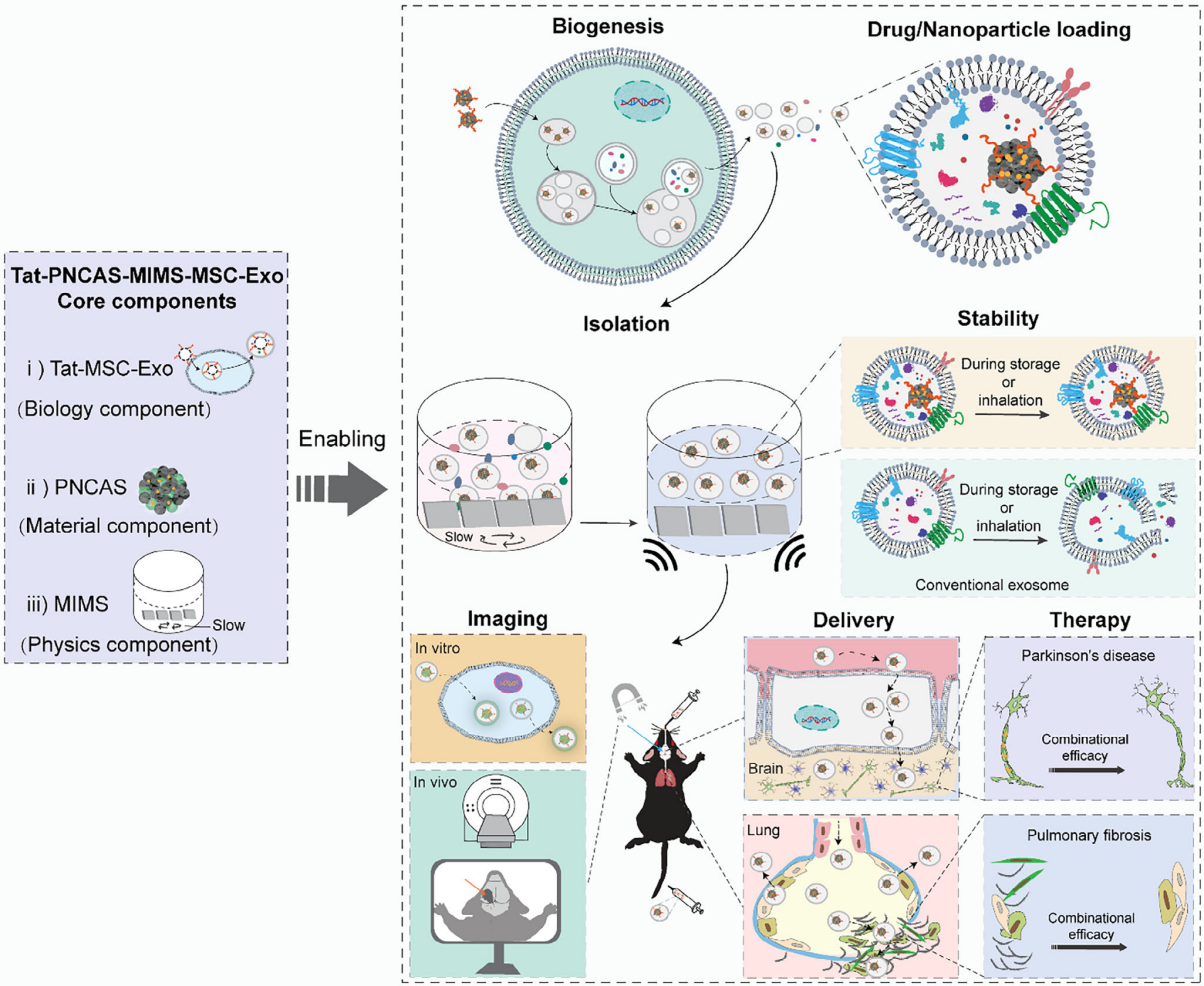
Schematic illustration of the integrated platform technology Tat‐PNCAS‐MIMS‐MSC‐Exo. The technology includes three core components, namely the biology component Tat‐MSC‐Exo, the material component PNCAS, and the physics component MIMS. These three components are linked by a simple operation, namely incubating MSCs with PNCAS‐Tat. The integrated technology enables drastic improvements in all four steps of the manufacturing flow of chemically engineered MSCs‐derived exosomes, namely biogenesis, drug/nanoparticle loading, isolation, and storage. Further, the presence of nanoparticles and drugs in exosomes enables enhanced or/and extra functionalities in the applications of exosomes, including imaging, delivery, and therapy. The drawing is not to scale.

In the present work, we solve these critical problems in realizing the above‐mentioned idea for producing chemically engineered MSCs‐derived exosomes. Our integrated technology, termed Tat‐PNCAS‐MIMS‐MSC‐Exo, incorporates three innovative core components (Figure 1): (1) The biology component Tat‐MSC‐Exo. We find that incubating MSCs with Tat peptide‐coated composite nanoparticles can not only stimulate the exosome biogenesis of MSCs by multiple folds, but also yield high endocytosis‐mediated uptake into MSCs, a substantial percentage of endocytosed composite nanoparticles being secreted from MSCs, and nearly all of the secreted composite nanoparticles being in exosomes. The idea of investigating Tat peptide (a HIV virus‐derived peptide) for this purpose was inspired by our recent publication on a comparative tracking study of the cellular transport of Tat peptide‐coated quantum dots (QDs) in MSCs vs. that in HeLa cells [34]. In that publication [34], the focus of the studies was on the intracellular transport of nanoparticles in MSCs in the context of improving delivery into the hard‐to‐transfect cells MSCs, whereas the focus of the present work is on the secretion of nanoparticles‐loaded exosomes from MSCs. Importantly, a key finding is a previously unknown nano‐effect, i.e., nanoparticles can amplify the exosome biogenesis‐stimulating effect of the ligands, making a relatively small stimulating effect of free Tat peptides become a large one with the use of Tat peptide‐coated nanoparticles. (2) The material component PNCAS. We adopt a type of composite nanoparticle called PNCAS (protein‐nanoparticle co‐assembly supraparticle) recently reported by us [35, 36]. PNCAS offers 50–100 nm physical diameter, excellent loading for both hydrophobic molecules (drugs) and hydrophobic nanoparticles (superparamagnetic iron oxide nanoparticles, i.e., SPIONs, QDs, etc.) in the same composite nanoparticle, as well as excellent structural and colloidal stability [35, 36]. (3) The physics component MIMS. We design a new magnetic isolation setup called MIMS (mobile internal magnetic separation). The MIMS design is readily scalable, and enables nearly complete isolation of PNCAS‐encapsulated exosomes from the extracellular medium within a short time duration. These three core components are linked by a simple operation, i.e., incubating MSCs with Tat peptide‐coated PNCAS (PNCAS‐Tat).

Below we describe the Tat‐PNCAS‐MIMS‐MSC‐Exo technology in detail. We demonstrate that combining the three core components (Tat‐MSC‐Exo, PNCAS, and MIMS) yields excellent performance in all four steps of the manufacturing flow of chemically engineered MSCs‐derived exosomes, namely biogenesis, loading of drugs and nanoparticles, isolation, and storage. We further show that, in comparison with native MSCs‐derived exosomes, the thus‐formed engineered MSCs‐derived exosomes offer enhanced or/and extra functionalities in biomedical applications, particularly in imaging, delivery, and therapy, due to the presence of nanoparticles and drugs in exosomes: the presence of SPIONs or/and QDs in exosomes allows for exosome imaging and tracking by magnetic resonance imaging (MRI), spinning‐disk confocal fluorescence microscopy, and transmission electron microscopy (TEM); the ultrahigh drug loading of PNCAS and the magnet‐responsive motion of SPIONs coupled with the barrier‐crossing ability of exosomes allows for enhanced delivery; the presence of a large amount of drugs in the exosomes allows for combinational effects in therapeutic efficacy between the drugs and exosomes (see Figure 1 for a schematic). It is worth pointing out that the primary focus of the present work is to develop a novel manufacture technology for chemically engineered exosomes. The secondary focus is to demonstrate the broad applicability of the products of the novel manufacture technology. And the tertiary focus is to explore the mechanisms of the nano‐bio interactions underlying the novel manufacture technology.

## 2 Results

### 2.1 Manufacture of Chemically Engineered MSCs‐Derived Exosomes: Exosome Biogenesis

**FIGURE 2:**
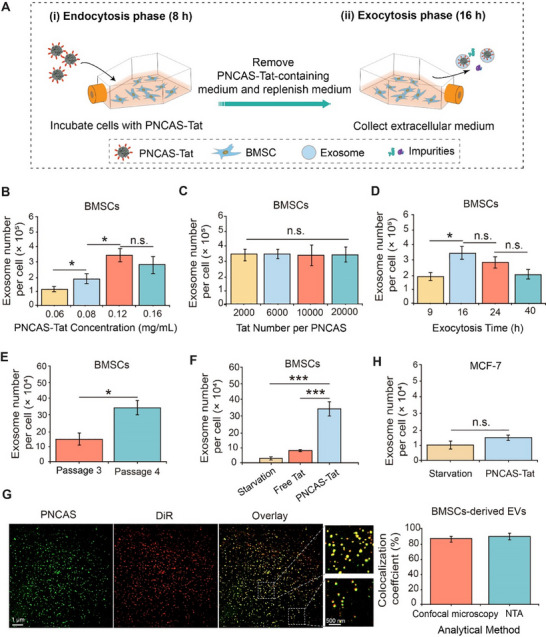
Stimulation effect of PNCAS‐Tat on exosome biogenesis. (A) Schematic of the procedure of using PNCAS‐Tat to stimulate exosome biogenesis in BMSCs. (B) Effect of the concentration of PNCAS‐Tat in cell culture medium on the number of exosomes generated per cell in BMSCs. Tat number per PNCAS used: 2000. Exocytosis time used: 16 h. Passage number used: 4. (C) Effect of the Tat number per PNCAS on the number of exosomes generated per cell in BMSCs. Concentration of PNCAS‐Tat used: 0.12 mg/mL. Exocytosis time used: 16 h. Passage number used: 4. (D) Effect of the time duration used for the exocytosis phase on the number of exosomes generated per cell in BMSCs. Concentration of PNCAS‐Tat used: 0.12 mg/mL. Tat number per PNCAS used: 2000. Passage number used: 4. (E) Effect of the passage number of BMSCs on the number of exosomes generated per cell in BMSCs. Concentration of PNCAS‐Tat used: 0.12 mg/mL. Tat number per PNCAS used: 2000. Exocytosis time used: 16 h. (F) Comparison of exosome number generated per cell in BMSCs by different methods: starvation (the common method) vs. free Tat peptide vs. PNCAS‐Tat. The conditions used in PNCAS‐Tat stimulation were: concentration of PNCAS‐Tat: 0.12 mg/mL; Tat number per PNCAS: 2000; exocytosis time: 16 h; BMSC passage number: 4. In the stimulation experiment of free Tat peptide, the number of Tat peptide molecules used was the same as that added for Tat conjugation with PNCAS in the PNCAS‐Tat stimulation experiment. (G) In the product after magnetic isolation, colocalization between PNCAS (containing QDs with green color) and exosomes (labeled by red vesicle dye DiR in the confocal fluorescence microscopy analysis, or analyzed by light scattering in the NTA measurement). The images are from confocal fluorescence microscopy. (H) In MCF‐7 cells, comparison of exosome number generated per cell by two different stimulation methods: starvation (the common method) vs. PNCAS‐Tat. The number concentrations of exosomes and PNCAS‐Tat were analyzed by NTA. Data in (B–H) are from three independent experiments and are presented as mean ± SD. In (B–D), data are analyzed with one‐way ANOVA followed by Tukey's multiple comparisons test. In (E), (F), (H), data are analyzed with a two‐tailed, unpaired *t*‐test. n.s., not significant; ^*^ *p* <0.05; ^***^ *p* <0.001.

**FIGURE 3:**
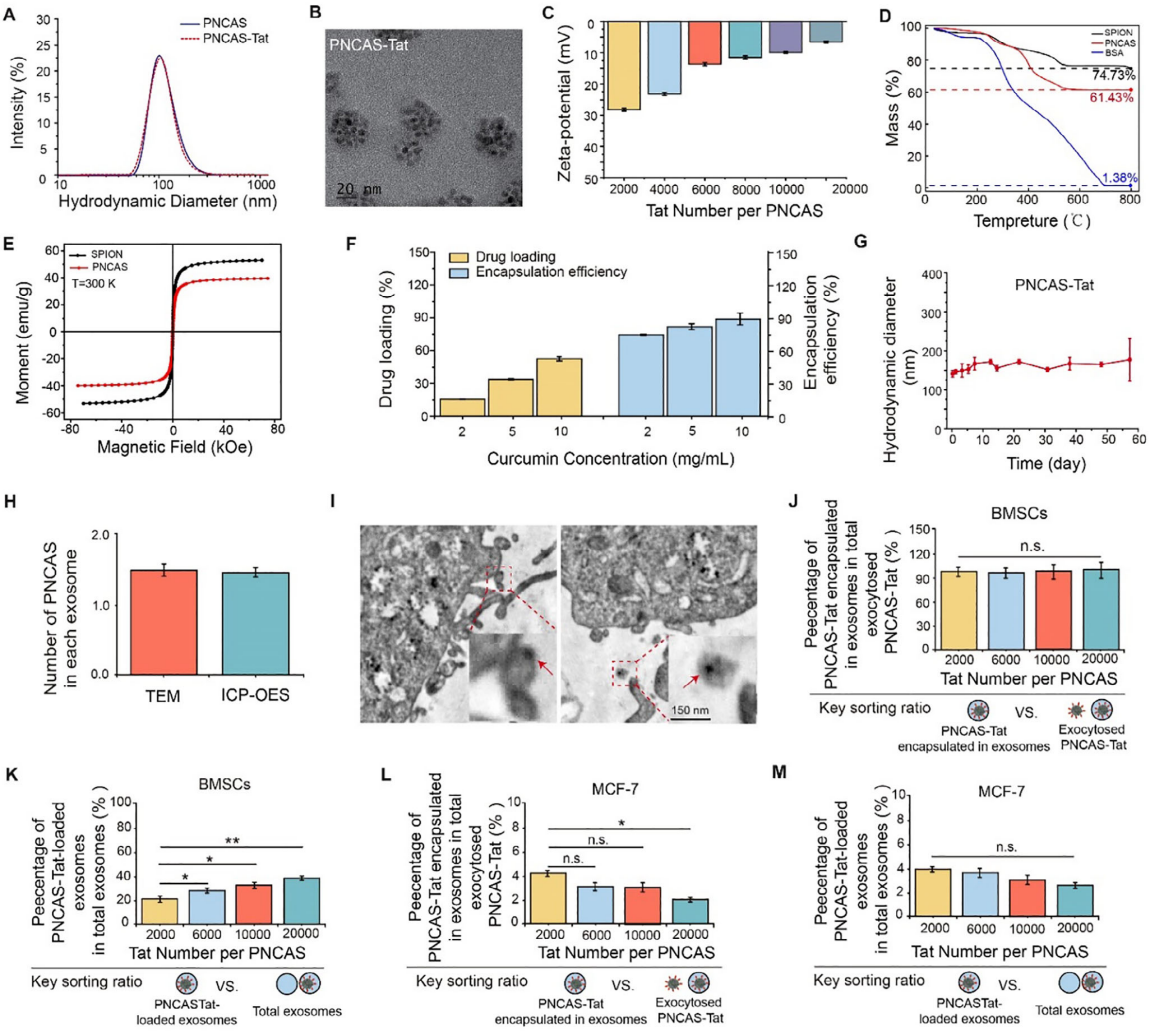
Drug and nanoparticle loading. (A) Dynamic light scattering (DLS) analysis of PNCAS and PNCAS‐Tat. (B) TEM of PNCAS‐Tat. Number of PNCAS‐Tat used to quantify the particle size: 50. Scale bar: 20 nm. (C) Zeta potential analysis of PNCAS‐Tat. (D) TGA of PNCAS. (E) Superconducting quantum interference device (SQUID) analysis of PNCAS. (F) Drug loading of PNCAS (drug: curcumin, or CUR). (G) Colloidal stability of CUR‐loaded PNCAS‐Tat in PBS at 4°C as measured by DLS. (H) Number of PNCAS loaded into each exosome of BMSCs, as quantified by two different methods namely TEM and ICP‐OES. Number of exosomes used to quantify the number of PNCAS in each exosome using TEM: 50. (I) Biological TEM for visualizing the exocytosis of PNCAS‐Tat in exosomes from BMSCs. (J) For BMSCs, quantification of the following key sorting ratio: percentage of PNCAS‐Tat encapsulated in exosomes in total exocytosed PNCAS‐Tat (the total exocytosed PNCAS‐Tat includes two fractions, i.e., PNCAS‐Tat encapsulated in exosomes and free PNCAS‐Tat in cell culture medium). (K) For BMSCs, quantification of the following key sorting ratio for BMSCs: percentage of PNCAS‐Tat‐loaded exosomes in total exosomes (the total exosomes include two fractions, i.e., PNCAS‐Tat‐loaded exosomes and exosomes without PNCAS‐Tat loading). (L) For MCF‐7 cells, quantification of the following key sorting ratio: percentage of PNCAS‐Tat encapsulated in exosomes in total exocytosed PNCAS‐Tat (the total exocytosed PNCAS‐Tat includes two fractions, i.e., PNCAS‐Tat encapsulated in exosomes and free PNCAS‐Tat in cell culture medium). (M) For MCF‐7 cells, quantification of the following key sorting ratio for BMSCs: percentage of PNCAS‐Tat‐loaded exosomes in total exosomes (the total exosomes include two fractions, i.e., PNCAS‐Tat‐loaded exosomes and exosomes without PNCAS‐Tat loading). In (C), (F), (G), (H), (J–M), data are from three independent experiments and are presented as mean ± SD. In (J–M), data are analyzed with one‐way ANOVA followed by Tukey's multiple comparisons test. n.s., not significant; ^*^ *p* <0.05; ^**^ *p* <0.01.

We found that incubating PNCAS‐Tat with bone marrow‐derived MSCs (BMSCs) caused a multi‐fold stimulation effect on the number of exosomes generated from BMSCs (the details on the composition, preparation, and physicochemical characterizations of PNCAS‐Tat will be described later in Figure 3). As schematically shown in Figure 2A, BMSCs were first incubated with a solution of PNCAS‐Tat in complete cell culture medium for 8 h (the endocytosis phase); after medium removal and phosphate saline buffer (PBS) washing for 3 times, the BMSCs were then incubated in PNCAS‐Tat‐free and serum‐free medium for 16 h (the exocytosis phase). The medium collected at the end of the exocytosis phase contained EVs secreted from the BMSCs. The number of exosomes in the medium was measured by nanoparticle tracking analysis (NTA), i.e., the number of microscopic objects in the diameter range 50–150 nm. We optimized the number of exosomes per cell with regard to concentration of PNCAS‐Tat, number of Tat peptide per PNCAS, time duration of the exocytosis phase, and passage number of BMSCs. Raising the concentration of PNCAS‐Tat incubated with BMSCs yielded an increased number of exosomes, until 0.12 mg/mL at which a plateau was reached (Figure 2B). Varying the number of Tat peptide per PNCAS in the range of 2000–20 000 essentially had no influence on the number of exosomes per cell (Figure 2C). A number above 20 000 caused considerable aggregation of PNCAS‐Tat in the cell culture medium. Increasing the time duration of the exocytosis phase up to 16 h resulted in a a higher number of exosomes per cell (Figure 2D). After 16 h, further prolonging the length of the exocytosis phase led to a reduction of the exosome number per cell (Figure 2D), indicating that endocytosis of the generated exosomes became significant. The 8 h time duration of the endocytosis phase was selected because it was the time point at which the net cellular uptake of PNCAS‐Tat reached a plateau (Figure S1). Using passage 4 of BMSCs produced more exosomes per cell than using passage 3 (Figure 2E). Only a narrow range of passage numbers of BMSCs could be used; outside the range, a lower passage number would give poor MSC purity, while a higher passage number would lead to MSC differentiation. In addition to producing more exosomes per cell than passage 3, passage 4 also showed slightly more molecular features of MSCs (that is, lower CD45 level, similar CD90 and CD44 levels, Figure S2). Under the optimized conditions (PNCAS‐Tat 0.12 mg/mL, Tat number per PNCAS 2,000, exocytosis phase 16 h, and BMSC passage number 4), the stimulation performance of PNCAS‐Tat on exosome biogenesis exhibited an ∼18‐fold improvement compared with that of the starvation treatment, the commonly‐used stimulation method (Figure 2F). Importantly, free Tat peptide (Tat peptide in the absence of PNCAS) caused only a ∼4‐fold increase in exosome secretion number over the starvation treatment (Figure 2F). Thus, these results suggest that being coated on the surface of PNCAS can amplify the stimulation effect of the Tat peptide.

**FIGURE 4:**
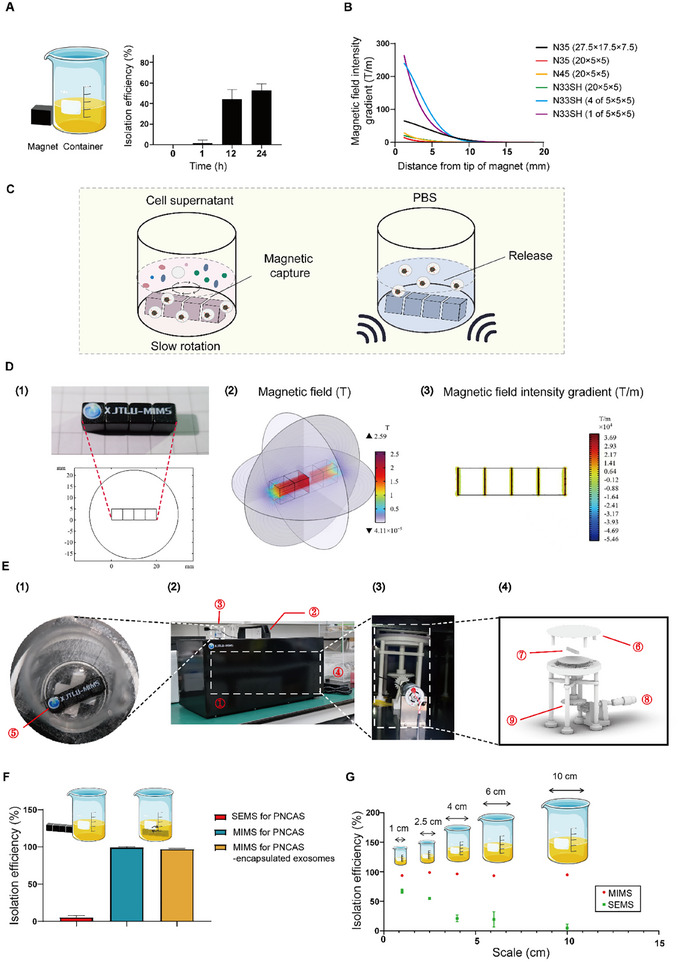
Employing the novel design of magnet setup, namely MIMS, for magnetic isolation. (A) Isolation efficiency of PNCAS in PBS at different times using the conventional magnet design. The magnet used was a N35 neodymium magnet (dimension 27.5 mm ×17.5 mm × 7.5 mm). The container used was a beaker with a 6 cm diameter bottom. A magnetic pole of the magnet was placed in contact with the outer surface of the container. (B) Magnetic field gradient as a function of distance from the tip (magnetic pole) of various neodymium magnets, including N35 (27.5 mm × 17.5 mm×7.5 mm, and 20 × 5 × 5 mm), N45 (20 mm × 5 mm × 5 mm), and N33SH (20 mm × 5 mm × 5 mm, 5 mm × 5 mm × 5 mm, and an assembly of 4 of 5 mm × 5 mm × 5 mm). The data were measured by a magnetometer. (C) Schematic of the overall design of MIMS. (D) Magnet used in MIMS. (1) A MIMS magnet is composed of multiple units of cubic magnets. A photo and a top‐view schematic of the magnet are shown (composed of 4 units of cubic magnets in this case). (2) Simulation of the 3D magnetic field distribution of the MIMS magnet. (3) Magnetic field gradient distribution from the top view of the MIMS magnet. White color indicates zero magnetic field gradient. (E) The instrument used in MIMS. A photo of the instrument prototype and a schematic are shown. (1) Sample loading area; (2) Instrument exterior view; (3) Internal structure of the instrument; (4) 3D model of the internal structure. ① Aluminum enclosure, ② Portable handle, ③ Sample container (beaker), ④ Power source, ⑤ Teflon‐coated capture magnet, ⑥ Support platform, ⑦ Drive magnet, ⑧ Drive motor, ⑨ Transmission gear. (F) A head‐to‐head comparison of isolation efficiency between the conventional magnet design (called SEMS here) and the novel magnet design (MIMS). For a head‐to‐head comparison, in SEMS and MIMS the same magnet was used, i.e., an assembly of 4 cubic magnet units. The isolation time duration examined was 1 h. The container used was a beaker with a 6 cm diameter. (G) Experimental demonstration of the unique scalability of the novel magnet design (MIMS), in comparison with the conventional magnet design (called SEMS here). With MIMS, for different container scales, near‐complete isolation was achieved using the same isolation time (1 h here). In comparison, with SEMS, after an isolation time 1 h, for a larger container scale, the isolation efficiency was much lower. In (A), (F), (G), data are from three independent experiments and are presented as mean ± SD. In (G), the standard deviations of the data points of MIMS are < 1.5% (the error bars are so small that they cannot be seen in the figure).

The identity of the exosome for the product collected by magnetic isolation (leveraging the MIMS magnet setup) was confirmed by transmission electron microscopy (TEM), NTA, and western blotting (Figure S3, the details on the design of MIMS will be described later in Figure 4). TEM of the product showed a classic “cup‐shaped” structure of an exosome (Figure S3A). The presence of PNCAS (containing the electron‐dense material SPION) in the exosome was also clearly visible in TEM (Figure S3A). NTA of the product (after magnetic isolation) showed that a dominant majority was in the diameter range 50–150 nm, and the average diameter was 128.4 nm (Figure S3B). It is worth mentioning that because NTA measures hydrodynamic diameter, which is usually somewhat (5%–50%) larger than physical diameter, the NTA peak somewhat larger than 150 nm in Figure S3B likely corresponds to exosomes as well. Western blotting showed the presence of three molecular biomarkers of exosomes, namely CD 63, TSG 101, and CD 81 (Figure S3C).

We assessed three aspects of the purity of the engineered exosome product. The percentage of the product (after magnetic isolation) in the hydrodynamic diameter range 50–150 nm and 50–200 nm were 80.0 ± 3.5% and 93.3 ± 1.9%, respectively, the latter of which accounts for the somewhat larger hydrodynamic size than physical size of exosomes (Figure S3B). We also analyzed two other aspects of purity of the engineered exosome product: (1) the percentage of product (after magnetic isolation) that had PNCAS encapsulated in the exosomes, as opposed to exosomes without PNCAS encapsulation; (2) the percentage of product (after magnetic isolation) that had PNCAS encapsulated in the exosomes, as opposed to PNCAS not encapsulated in exosomes (that is, free PNCAS). We employed a version of PNCAS that co‐encapsulated QDs (fluorescent nanoparticles) and SPIONs for the exosome biogenesis and isolation, and then used colocalization with a vesicle dye to study the PNCAS encapsulation in the product (after magnetic isolation). The colocalization between PNCAS (with fluorescence from QDs) and exosomes (with fluorescence from vesicle dye) were quantified by confocal fluorescence microscopy to be 90.36%, indicating that the PNCAS population and the vesicle population were near‐perfectly colocalized in the product (after magnetic isolation) (Figure 2G). We also compared the NTA results from two different modes, namely fluorescence mode and scattering mode, to analyze the PNCAS encapsulation in the product (after magnetic isolation). The exosome number concentration result from the fluorescence mode gave the number concentration of PNCAS‐encapsulated exosomes, while the exosome number concentration from the scattering mode gave the number concentration of all exosomes. Dividing these two values gave the percentage 92.35%, which was close to the confocal fluorescence microscopy result (90.36%) (Figure 2G). Taking the results of Figure S3B and Figure 2G together, it was concluded that the purity of the product (after magnetic isolation) was high: nearly all of the particulate contents were PNCAS‐encapsulated exosomes.

We found that the stimulation effect of PNCAS‐Tat on exosome biogenesis was cell type‐dependent. Using MCF‐7 cells (a breast cancer cell line) instead of BMSCs to incubate with PNCAS‐Tat resulted in only a very small, if any, stimulation effect on exosome biogenesis (Figure 2H). Varying the Tat number per PNCAS between 2000 and 20 000 did not significantly change the exosome number per cell in MCF‐7 cells (Figure S4). The mean sizes of exosomes produced from different cell types (BMSCs or MCF‐7 cells) and by different methods (PNCAS or starvation) were nearly identical (Figure S5).

### 2.2 Manufacture of Chemically Engineered MSCs‐Derived Exosomes: Drug and Nanoparticle Loading

The design of PNCAS harnessed our recent finding in co‐assembly of proteins and nanoparticles, i.e., simple mixing of hydrophobic nanoparticles (SPIONs or/and QDs) and bovine serum albumin (BSA) led to spontaneous and rapid formation of a highly stable supraparticle structure [35], which can further offer exceptionally high loading of drugs [36]. After the formation of PNCAS, we conjugated it with Tat peptide using SMCC [succinimidyl‐4‐(N‐maleimidomethyl)cyclohexane‐1‐carboxylate] conjugation chemistry. The hydrodynamic diameter of PNCAS‐Tat, as measured by dynamic light scattering (DLS), was 108.5 ± 1.5 nm with polydispersity (PDI) 0.226 ± 0.047, which was only slightly larger than that before the Tat conjugation (104.7 ± 0.9 nm, PDI 0.197 ± 0.039) (Figure 3A). TEM of PNCAS‐Tat showed the presence of multiple electron‐dense nanoparticles in each PNCAS, and the physical diameter 54.6 nm ± 1.8 nm (Figure 3B). Although it was difficult to determine the exact number of SPIONs in each PNCAS, the TEM images showed that this number was quite large: on just one imaging focal plane, 10‐20 SPIONs were visible in each PNCAS (Figure 3B). Adding a larger number of Tat peptide to each PNCAS in the conjugation reaction led to a more positive surface charge in PNCAS‐Tat, as indicated by a more positive value of zeta potential, due to the positive charge of Tat peptide (Figure 3C). Gradually increasing the Tat number added to each PNCAS from 2000 to 20 000 resulted in a gradual change of the zeta potential value from −28.21 ± 0.47 mV to −6.54 ± 0.32 mV (Figure 3C). The Tat number shown in Figure 3C refers to the addition amount (amount of Tat added to the conjugation reaction mixture) rather than the actual amount conjugated to PNCAS. However, these two amounts (the added amount and the conjugated amount) were closely correlated, that is a higher added amount gives rise to a higher conjugated amount, as indicated by the gradually more positive zeta potential with more added amount of Tat shown in Figure 3C. Thermogravimetric analysis (TGA) results of PNCAS, in comparison with those of SPIONs and BSA, indicated that the weight percentage of SPIONs in PNCAS was ∼81.87% (Figure 3D). The magnetic hysteresis loops measured by SQUID (superconducting quantum interference device) showed that both PNCAS and SPIONs were superparamagnetic (zero remanence and zero coercivity) at 300 K (Figure 3E). The saturation magnetization of PNCAS and SPIONs as measured by SQUID was 39.08 and 53.14 emu/g, respectively (Figure 3E). The ratio of these two values was 73.7%, which was fairly close to the weight percentage of SPIONs in PNCAS measured by TGA, suggesting that the interactions among the large number of SPIONs in a PNCAS were small (if any), not sufficient to significantly influence the magnetic properties.

We employed curcumin (CUR) as the model drug in the present work. CUR is a widely available, food‐derived compound with reported anti‐inflammatory, anti‐oxidant, and neuroprotective activities [37, 38, 39, 40]. We applied our recently reported drug loading method to load CUR in PNCAS [36]. An increase in the addition amount of CUR yielded an increase in the drug loading and encapsulation efficiency, with the maximum drug loading being ∼53% and encapsulation efficiency being ∼90% (Figure 3F). The CUR‐loaded PNCAS‐Tat maintained excellent colloidal stability, as indicated by the low level of change in hydrodynamic diameter during the 2‐month storage in PBS at 4°C (Figure 3G). The drug release from CUR‐loaded PNCAS‐Tat displayed a gradual release kinetics, with ∼40% cumulative drug release by 500 h (Figure S6).

We analyzed the average number of PNCAS loaded in each exosome in the product (after magnetic isolation) using two different methods, namely TEM (microscopy) and ICP‐OES (elemental analysis, inductively coupled plasma optical emission spectrometry). These two methods yielded close results, i.e., on average ∼1.5 PNCAS were loaded into each exosome in the product (after magnetic isolation) (Figure 3H; Figure S3A, a biological TEM image is shown in Figure 3I to visualize the exocytosis of PNCAS‐Tat in exosomes from BMSCs). This suggests that in each exosome in the product (after magnetic isolation), either 1 or 2 PNCAS was loaded, and the number of exosomes with 1 loaded‐PNCAS approximately equaled to the number of exosomes with 2 loaded‐PNCAS. Given the ∼53% CUR maximum loading capacity to each PNCAS (Figure 3F), it can be calculated that at maximum ∼1.0275 × 10^6^ (that is, ∼1 million) CUR molecules can be loaded into each exosome using PNCAS‐Tat (see Note S1 for the calculation).

We quantified several key sorting ratios in cellular transport relevant to the loading of PNCAS‐Tat into exosomes, including (1) the percentage of PNCAS‐Tat encapsulated in exosomes in total exocytosed PNCAS‐Tat (the total exocytosed PNCAS‐Tat includes two fractions, i.e., PNCAS‐Tat encapsulated in exosomes and free PNCAS‐Tat in extracellular medium, see Figure 3J for a schematic); (2) the percentage of PNCAS‐Tat‐loaded exosomes in total exosomes (the total exosomes include two fractions, i.e., PNCAS‐Tat‐loaded exosomes and exosomes without PNCAS‐Tat loading, see Figure 3K for a schematic); (3) the percentage of exocytosed PNCAS‐Tat in endocytosed PNCAS‐Tat (i.e., the percentage of what gets released from the cells, in the total population of PNCAS‐Tat that have entered the cells) (Figure S7F).

First, we analyzed the percentage of PNCAS‐Tat encapsulated in exosomes in total exocytosed PNCAS‐Tat from BMSCs (the first key sorting ratio, Figure 3J). We applied fluorescence colocalization on the cell culture medium after the exocytosis phase (before magnetic isolation), to quantify the percentage of green color (PNCAS‐Tat, containing QDs) that was colocalized with red color (vesicle dye). This percentage was found to be >90% for BMSCs (Figure 3J). Second, we investigated the percentage of PNCAS‐Tat‐loaded exosomes in total exosomes from BMSCs (the second key sorting ratio, Figure 3K). We applied fluorescence colocalization on the cell culture medium after the exocytosis phase (before magnetic isolation), to quantify the percentage of red color (vesicle dye) that was colocalized with green color (PNCAS‐Tat, containing QDs). It should be noted that this measurement was conducted before magnetic isolation, so that the fraction of exosomes without PNCAS‐Tat loading was kept for the measurement. From this colocalization analysis, we found that the percentage of PNCAS‐Tat‐loaded exosomes in total exosomes from BMSCs was in the range of ∼20%–40%, and interestingly that it displayed a clear dependence on the Tat number per PNCAS (Figure 3K). A higher Tat number per PNCAS varying from 2000 to 20 000 gave rise to a larger value of the percentage in BMSCs (Figure 3K).

Intrigued by the origin of this trend about the second key sorting ratio (Figure 3K), we investigated the dependence of the following three parameters relevant to this key sorting ratio on the Tat number per PNCAS, i.e., the cellular uptake of PNCAS‐Tat, the percentage of exocytosed PNCAS‐Tat to endocytosed PNCAS‐Tat (that is, the third key sorting ratio mentioned above), and the total number of exosomes generated from a BMSC. Using confocal fluorescence microscopy and flow cytometry, we found that the cellular uptake of PNCAS‐Tat into BMSCs was increased with a higher Tat number per PNCAS (Figure S7A–C). Furthermore, the percentage of exocytosed PNCAS‐Tat to endocytosed PNCAS‐Tat in BMSCs remained nearly unchanged (∼75%) with varying Tat number per PNCAS, as measured by flow cytometry (Figure S7F); and the total number of exosomes generated from a BMSC also remained nearly constant with varying Tat number per PNCAS (Figure 2C). These results, taken together, thus suggest that a higher Tat number per PNCAS led to an increase in cellular uptake in BMSCs yet no change in the ratio of exocytosed PNCAS‐Tat to endocytosed PNCAS‐Tat and no change in the total number of secreted exosomes, which in turn yielded a higher percentage of PNCAS‐encapsulated exosomes among all the secreted exosomes from BMSCs.

PNCAS‐Tat appears to have strong affinity with membranes in cells: after only 5 min of incubation time, a considerable amount of PNCAS‐Tat was already bound to the cell membrane (some may have already entered the cells) as imaged by confocal fluorescence microscopy, and a higher Tat number per PNCAS may lead to stronger affinity with cell membrane and greater amount of cellular uptake (Figure S8A,B). Even after being internalized into BMSCs, under TEM PNCAS‐Tat was often seen to adhere to the inner membranes of intracellular vesicles (Figure S8C). This is also consistent with the observation that in exosomes PNCAS‐Tat was often localized near the membranes (Figure 3I). A control experiment using PNCAS without Tat coating showed no significant PNCAS uptake into BMSCs, suggesting that interaction with Tat peptide, rather than with PNCAS, caused the biological responses of BMSCs (Figure S7A,B). It is worth pointing out that a large quantity of internalized PNCAS‐Tat and a large majority (∼75%) of internalized PNCAS‐Tat getting exocytosed are both beneficial features of our technology (Figure S7F).

In addition, we found that the sorting in cellular transport of PNCAS‐Tat was cell type‐dependent. In MCF‐7 cells, the percentage of PNCAS‐Tat encapsulated in exosomes in total exocytosed PNCAS‐Tat was only ∼5%, and the percentage of PNCAS‐Tat‐loaded exosomes in total exosomes was also only ∼5% (with the Tat number per PNCAS varying from 2000 to 20 000) (Figure 3L,M, respectively). These two percentages of MCF‐7 cells were much lower than those of BMSCs (Figure 3J,K, respectively).

### 2.3 Manufacture of Chemically Engineered MSCs‐Derived Exosomes: Magnetic Isolation

Due to the small size of PNCAS and the fact that the inner core of an exosome can only hold a small number (close to one) of PNCAS, magnetic isolation of the PNCAS‐encapsulated exosomes from the extracellular medium needs to overcome a crucial challenge, i.e., the design of the magnet setup. A radically different design of a magnet setup from existing ones is needed. This need can be seen from analyzing the fundamental equation of magnetic isolation of a magnetic particle in a fluid:

$$F_{\mathrm{mag}}=\mu_{0}\;\frac{4}{3}\pi r^{3}M_{p}\nabla H$$

where F_mag_ represents the magnetic force, µ_0_ is the permeability of free space (4π × 10^−7^ H · m^−1^), r is the radius (half of the diameter) of the magnetic particle, M_p_ is the saturation magnetization, and ∇H is the gradient of the external magnetic field. Because in our technology the magnetic particle is encapsulated in the exosome, the physical diameter of the particle must be smaller than that of the exosome (50–150 nm in diameter, which is 1–2 order of magnitude smaller than the cell and magnetic particles typically used for exosome isolation). In turn, the magnetic force is restricted by this fundamental limitation, governed by the above equation. Indeed, our experimental results showed that only ∼1% isolation efficiency of PNCAS (measured by NTA) was achieved after 1 h of isolation with the conventional magnetic setup, i.e., placing a magnet (neodymium N35 magnet, dimension 27.5 mm ×17.5 mm × 7.5 mm, here) in contact with the outer surface of a container (beaker with 6 cm diameter here) of PNCAS dispersion (Figure 4A). Furthermore, even if a stronger magnet is used, the magnetic field gradient still drops to nearly zero at a close distance (∼10 mm) from the edge of the magnet (Figure 4B). Thus, the conventional design of magnet setup yields poor performance in scaling up: a larger sample container leads to dramatically longer isolation time (see Note S2 for a simplistic calculation, and Figure 4G for an experimental demonstration). PNCAS was used here as a convenient proxy for PNCAS‐encapsulated exosomes to evaluate magnetic isolation, as each PNCAS‐encapsulated exosome contains on average ∼1.5 PNCAS as indicated by the results in Figure 3H. To overcome this problem, the only realistic possibility is to dramatically raise the magnetic field gradient ∇H in the above equation, by using alternative magnet setups. Some of the previously‐reported magnet setups, e.g., microfluidic magnetic device [32] and quadruple magnetic flow sorter [31, 33], might be helpful to increase the magnetic force, but they suffer from low scalability and sometimes high cost. Here, we present a new design termed MIMS to solve this problem.

MIMS increases the magnetic field gradient at the magnetic particle by drastically shortening the distance between the magnet and the magnetic particle. As mentioned above, there is a rapid reduction in magnetic field gradient with increasing distance from the magnet (Figure 4B). The magnet can only produce a high magnetic field gradient close to the magnet surface. In MIMS, the distance is shortened by (1) placing the magnet inside the dispersion of magnetic particles (hence the word “internal” in “MIMS”) and (2) rotating the magnet (hence the word “mobile” in “MIMS”). With the MIMS design, essentially every PNCAS‐encapsulated exosome is brought very close to the surface of the magnet. A MIMS cycle includes two phases, namely the capture phase and the release phase (Figure 4C). In the capture phase, a magnet rotates slowly (e.g., 5 rpm) in the extracellular medium, capturing PNCAS‐encapsulated exosomes along the way. In the release phase, after the medium is replaced with PBS, the magnet is treated by a brief bath sonication to release the captured PNCAS‐encapsulated exosomes into PBS. It is essential to use a slow rotation speed for the magnet in the capture phase, in order to minimize liquid flows opposite to the motion direction of magnetic capture.

In MIMS, the capture magnet is an assembly of multiple units of cubic magnet, with a thin inert polytetrafluoroethylene (PTEE, also called Teflon) coating to prevent corrosion (Figure 4D(1)). Figure 4D(2) shows the magnetic field distribution around the magnet, as determined by finite element simulation using the Ansys Maxwell software. As shown in Figure 4D(3), in the magnetic field gradient distribution of the MIMS capture magnet, the much higher magnetic field gradient close to the magnet than that far away from the magnet is clearly visible. Crucially, there are maximums of magnetic field gradient at all the junctions between neighboring unit cubic magnets (Figure 4D(3)). Thus, with the MIMS capture magnet slowly rotating in a dispersion of magnetic objects, these maximums along the entire length of the capture magnet can generate strong magnetic attraction forces at virtually every location in the dispersion. To perform magnetic isolation for a larger scale of dispersion, one can simply employ a MIMS capture magnet consisting of a larger number of units of a cubic magnet to ensure that every magnetic object in the dispersion can be brought close to the magnet.

We built a prototype of an electric motor‐controlled instrument to achieve automatic operation of MIMS. As shown in Figure 4E, the MIMS instrument integrates a direct‐current power supply, an electric motor, and a mechanical assembly. In the mechanical assembly, the motor's torque output, generated within a vertical plane, is transferred to a horizontal plane via perpendicular bevel gears, thereby rotating a drive magnet horizontally. This mechanical mechanism is designed to maintain a consistent, low‐speed rotation for the drive magnet fixed on a platform. The capture magnet is situated within a container, e.g., a beaker, with the liquid dispersion to be processed, on top of the drive magnet. The capture magnet is synchronized with the drive magnet to rotate at an identical speed in the container.

Figure 4F shows the isolation efficiency results of a head‐to‐head comparison between the novel design MIMS and the conventional design (called SEMS here, i.e., static external magnetic separation), using the same permanent magnet (an assembly of 4 cubic magnet units). With the MIMS design, after 1 h, >95% isolation efficiency for both PNCAS and PNCAS‐encapsulated exosomes was achieved. In stark contrast, with the SEMS design, after the same time duration (1 h), only ∼1% isolation efficiency of PNCAS was achieved. Furthermore, a crucial advantage of MIMS is its unique scalability: a larger scale of sample container requires essentially identical isolation time to achieve near‐complete isolation, using a larger number of cubic magnet units (see Note S2 for a simplistic calculation). Scalability is a bottleneck challenge in utilizing magnetic isolation for production, which usually requires larger scales than analytical applications. In an experimental demonstration of this scalability of MIMS, Figure 4G shows the isolation efficiency results after 1 h for 5 different scales of containers (1, 2.5, 4, 6, and 10 cm of the diameters of beakers) of PNCAS dispersion with MIMS vs. SEMS. With the MIMS design, for all the 5 different scales of containers, the isolation efficiency reached >95% after 1 h (Figure 4G). In contrast, with the SEMS design, after 1 h, the isolation efficiency decreased greatly with larger scales of container (Figure 4G). With the largest scale of container tested (10 cm), the isolation efficiency given by SEMS after 1 h was only ∼1% (Figure 4G). In addition, the MIMS design yielded remarkable reproducibility in isolation efficiency: <1.5% in standard deviations from three independent isolation experiments, which were greatly superior to those with the SEMS design, especially with the larger scales (Figure 4G). The release phase in a MIMS cycle can be completed in as short as 10 s; with bath sonication and some additional rinsing of the capture magnet, we were able to recover >90% of PNCAS or PNCAS‐encapsulated exosomes that had been captured (Figure S9). The integrity of PNCAS‐encapsulated exosomes remained intact during the MIMS process, as indicated by TEM (Figure S3A) and the close number values of PNCAS‐encapsulated exosomes measured before and after the MIMS process.

In addition, in order to explore the general broad applicability of the MIMS design, we conducted preliminary experiments on cell isolation with commercial antibody‐conjugated magnetic nanobeads. We labeled Jurkat leukemia T cells with the commercial MagDot 580 anti‐human CD45, and compared the magnetic isolation performance of MIMS and the conventional SEMS design. Indeed, the result showed a large improvement in isolation efficiency using MIMS in the same time frame (Figure S10). It is worth noting that, because in this cell isolation experiment each cell was attached to a large number of magnetic nanoparticles (MagDots, with a similar particle size as PNCAS), it was easier to isolate cells in this way than to isolate exosomes with just 1 to 2 PNCAS inside the exosomes, given the much larger magnetic force generated on each cell. Lastly, it is worth pointing out that MIMS should not be confused with magnetic stirring, although they bear some superficial resemblance (e.g., in both techniques a magnet rotates in a sample container). The purpose of magnetic stirring is to mix; thus the rotation speed is fast and a regular magnet (with maximums of magnetic field gradient only at the two ends of the magnet) is sufficient for the purpose. In contrast, the purpose of MIMS is to capture; thus the rotation speed is slow, and the magnet used has multiple maximums of magnetic field gradients (Figure 4D).

### 2.4 Manufacture of Chemically Engineered MSCs‐Derived Exosomes: Stability

**FIGURE 5:**
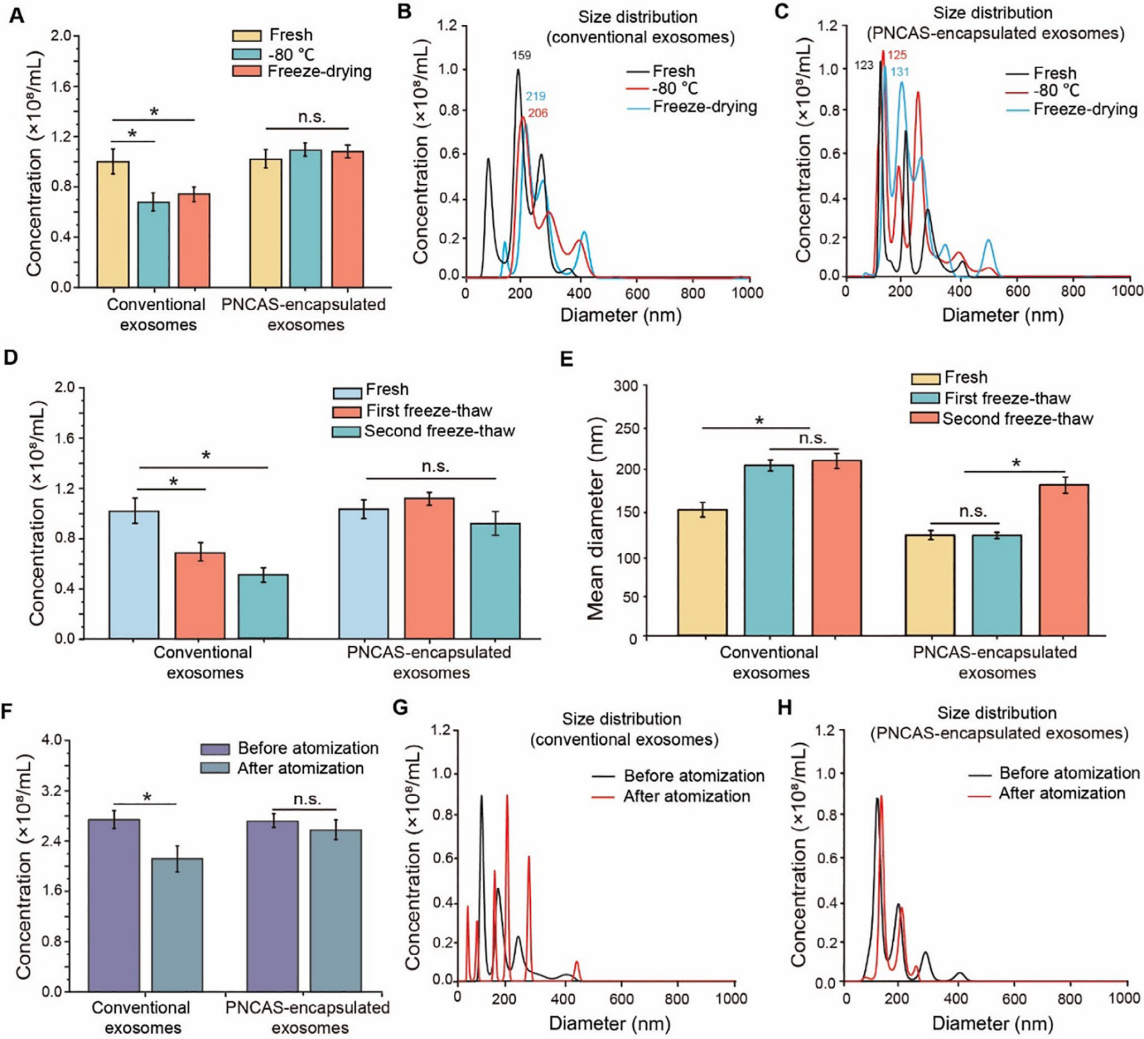
Stability improvement of exosomes offered by PNCAS encapsulation. (A) Change of particle number concentration due to storage conditions (−80°C and freeze drying) for different exosomes: conventional exosomes vs. PNCAS‐encapsulated exosomes. Storage time duration used: 1 month. (B,C) Change of particle size distribution due to storage conditions (−80°C and freeze drying) for different exosomes: conventional exosomes (B) vs. PNCAS‐encapsulated exosomes (C). Storage time duration used: 1 month. (D) Change of particle number concentration due to the first and second freeze‐drying cycles for different exosomes: conventional exosomes vs. PNCAS‐encapsulated exosomes. (E) Change of mean particle diameter due to the first and second freeze‐drying cycles for different exosomes: conventional exosomes vs. PNCAS‐encapsulated exosomes. (F) Change of particle number concentration due to a stress treatment (atomization) used in applications for different exosomes: conventional exosomes vs. PNCAS‐encapsulated exosomes. (G,H) Change of particle size distribution due to a stress treatment (atomization) used in applications for different exosomes: conventional exosomes (G) vs. PNCAS‐encapsulated exosomes (H). In (A), (D), (E), (F), data are from three independent experiments and are presented as mean ± SD. For (A, D, E), data are analyzed with one‐way ANOVA followed by Tukey's multiple comparisons test. For (F), data are analyzed with a two‐tailed, unpaired *t*‐test. n.s., not significant; ^*^ *p* <0.05.

We found that the presence of PNCAS in the PNCAS‐encapsulated exosomes significantly enhanced the stability of exosomes during storage and against stress conditions involved in some applications. To assess the potential stability enhancement effect during storage, we examined two storage conditions commonly used for exosomes, namely −80°C (typically for shorter‐term storage) and freeze drying (typically for longer‐term storage). The phase changes involved in these storage conditions are the primary causes of potential stability loss. NTA analysis showed that, for exosomes without PNCAS encapsulation, there was a significant (∼30%) reduction of number of exosomes (microscopic objects in the size range of exosomes) after 1‐month storage at −80°C or as lyophilized powder after freeze drying (Figure 5A). In contrast, for PNCAS‐encapsulated exosomes (isolated by MIMS), no significant reduction of exosome number occurred (Figure 5A). Further analysis of the size distribution by NTA revealed that, for the exosomes without PNCAS, the diameter of the largest portion increased from 159 nm (before storage) to 206 nm (after 1‐month storage at −80°C) and to 219 nm (after 1‐month storage by freeze drying), respectively (Figure 5B). In contrast, such changes were minimal for PNCAS‐encapsulated exosomes (isolated by MIMS): the diameter of the largest portion only changed from 123 nm (before storage) to 125 nm (after 1‐month storage at −80°C) and to 131 nm (after 1‐month storage by freeze drying), respectively (Figure 5C). We then focused on our studies on the freeze‐thaw cycles, which are the main causes of potential stability loss in storage under low temperature or by freeze drying. We found that the first freeze‐thaw cycle significantly reduced the number of exosomes and increased the mean size given by NTA for the exosomes without PNCAS encapsulation (Figure 5D,E). In contrast, for the PNCAS‐encapsulated exosomes (isolated by MIMS), no significant changes in the exosome number and mean size were found by NTA after one freeze‐thaw cycle (Figure 5D,E). After the second freeze‐thaw cycle, the PNCAS‐encapsulated exosomes (isolated by MIMS) maintained the exosome number, but showed a significant increase in the mean size by NTA (Figure 5D,E). This suggests that during storage of our PNCAS‐encapsulated exosome product, the freeze‐drying treatment should be limited to no more than one cycle (if no additional cryoprotection method is used).

Encouraged by the stabilizing effect found during storage (Figure 5A–E), we further assessed the stability of exosomes against other stress conditions. We examined the effect of nebulization (atomization inhalation), a common delivery method to the lung, on exosome stability using a commercial nebulizer. Indeed, as measured by NTA, PNCAS‐encapsulated exosomes (isolated by MIMS) showed no significant changes in exosome number and size distribution, while exosomes without PNCAS encapsulation displayed a reduction in exosome number and new NTA peaks with sizes larger than exosomes (Figure 5F–H).

### 2.5 Manufacture of Chemically Engineered MSCs‐Derived Exosomes: Quality Control

We conducted quality control optimization on the manufacturing flow of Tat‐PNCAS‐MIMS‐MSC‐Exo, to maximize the reproducibility and minimize the contamination. We achieved excellent reproducibility of the manufacturing flow. We assigned 3 different operators to conduct the manufacturing, with each operator processing 3 samples in parallel to complete the entire manufacturing flow. The results indicated only small variations both within the same operator and between different operators. For the hydrodynamic diameter of CUR‐loaded PNCAS, the deviations within the same operator and between different operators were both <10% (Figure S11A). For the zeta potential (surface charge) of CUR‐loaded PNCAS, the deviations within the same operator and between different operators were both <15% (Figure S11B). For the drug loading of CUR‐loaded PNCAS, the deviations within the same operator and between different operators were both <5% (Figure S11C). For the drug encapsulation efficiency of CUR‐loaded PNCAS, the deviations within the same operator and between different operators were both <10% (Figure S11C). For the number of exosomes generated per cell (before magnetic isolation), the deviations within the same operator and between different operators were both <10% (Figure S11D). For the percentage of PNCAS‐encapsulated exosomes (isolated by MIMS) in the total exosome population, the deviations within the same operator and between different operators were both <5% (Figure S11E).

We also achieved minimal contamination of the manufacturing flow. We used a well‐established surrogate, namely the endotoxin level, for the overall contamination control. Endotoxin contamination is ubiquitous, highly stable, and resistant to standard sterilization methods. Trace amounts of endotoxin can trigger severe immune responses, making it a critical safety concern for injectable drugs, medical devices, and biologics. Regulatory Agencies (e.g., FDA, EMA, CFDA) and pharmacopeias (e.g., USP, EP, ChP) mandate strict endotoxin limits (e.g., ≤0.25 EU/mL for sterile water for injection and irritation). Controlling endotoxin level indirectly ensures general contamination control. Here, through strict control of aseptic practices, raw materials (e.g., using low‐endotoxin BSA for producing PNCAS), water quality, and purification, we were able to control the endotoxin level of the products to be much lower than the allowed limits (Figure S12).

### 2.6 Mechanistic Studies of MSCs‐Derived Exosome Biogenesis With Stimulation by PNCAS‐Tat

The mechanistic studies sought to answer the following questions: (1) what is the biological pathway of MSCs‐derived exosome biogenesis in the presence of PNCAS‐Tat? (2) is there significant cell type dependence for the biological pathway of exosome biogenesis in the presence of PNCAS‐Tat? (3) is there a difference in the molecular content of MSCs caused by the presence of PNCAS‐Tat? (4) is there a difference in the molecular content of MSCs‐derived exosomes caused by the presence of PNCAS‐Tat?

**FIGURE 6:**
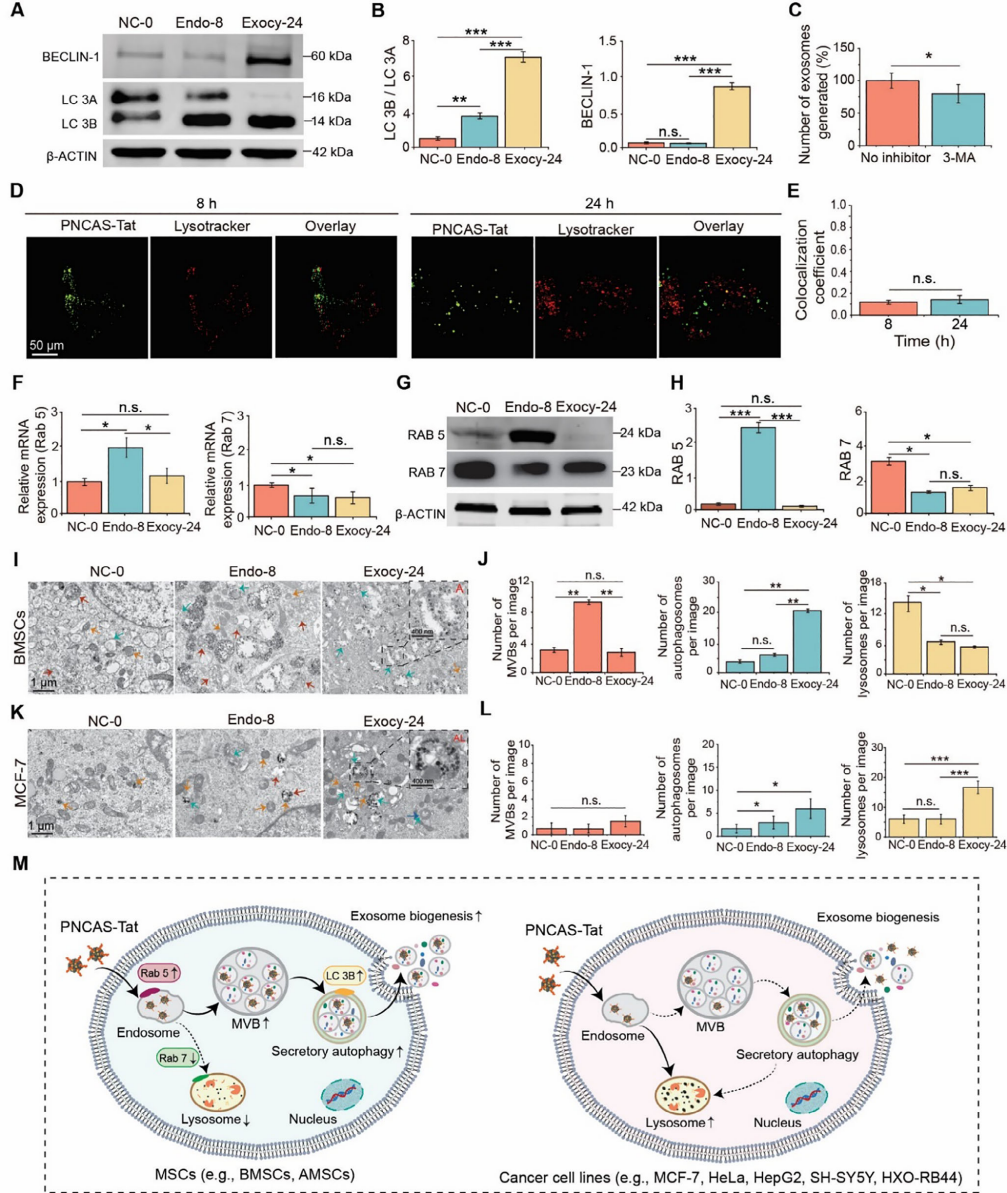
Mechanism studies of exosome biogenesis in the presence of PNCAS‐Tat. In this figure NC‐0 refers to cells without PNCAS‐Tat at time point 0; Endo‐8 refers to cells with PNCAS‐Tat at 8 h (endocytosis phase); Exocy‐24 refers to cells with PNCAS‐Tat at 24 h (exocytosis phase). (A) Protein levels of molecular markers of autophagy in BMSCs, as analyzed by western blotting. (B) Quantification results of (A). (C) Effect of the autophagy inhibitor 3‐MA on the relative amount of exosomes from BMSCs stimulated by PNCAS‐Tat. (D) Colocalization of PNCAS‐Tat (containing green QDs) with lysosomes (labeled by red LysoTracker) in BMSCs. Scale bar: 50 µm. (E) Quantification of Manders colocalization coefficient based on images from (D). n = 100 cells. Data are presented as mean ± SD. (F) mRNA expression analysis of Rab GTPases (Rab 5 mediates formation of early endosome; Rab 7 mediates fusion with lysosomes), as measured by real‐time qPCR. (G) Protein expression analysis of Rab GTPases, namely Rab5 and Rab7, as measured by western blotting. (H) Quantification results of (G). (I) TEM imaging of the cellular transport of PNCAS‐Tat in BMSCs. Red arrows point to MVBs; Blue arrows point to autophagosomes; yellow arrows point to lysosomes. A: autophagosome. Scale bar: 1 µm. (J) Quantification analysis of the numbers of MVBs, autophagosomes, and lysosomes in BMSCs, based on the images from (I). n = 8 cells. Data are presented as mean ± SD. (K) TEM imaging of the cellular transport of PNCAS‐Tat in MCF‐7 cells. Red arrows point to MVBs; Blue arrows point to autophagosomes; yellow arrows point to lysosomes. AL: autolysosome. Scale bar: 1 µm. (L) Quantification analysis of the numbers of MVBs, autophagosomes, and lysosomes in MCF‐7 cells, based on the images from (I). n = 8 cells. Data are presented as mean ± SD. (M) Schematic of the biological pathways of cellular transport of PNCAS‐Tat: MSCs vs. cancer cell lines. In MSCs, the cellular transport of PNCAS‐Tat primarily goes through the secretary autophagy pathway, and stimulates a large amount of MVB formation and exosome biogenesis. In contrast, in cancer cell lines, the cellular transport of PNCAS‐Tat primarily goes through the endosome‐lysosome pathway, and stimulates no (or only a small amount of) MVB formation and exosome biogenesis. For (B), (C), (F), (H), data are from three independent experiments and are presented as mean ± SD. For (B, F, H, J, L), data are analyzed with one‐way ANOVA followed by Tukey's multiple comparisons test. For (C, E), data are analyzed with a two‐tailed, unpaired *t*‐test. n.s., not significant; ^*^ *p* <0.05; ^**^ *p* <0.01; ^***^ *p* <0.001.

Several lines of results suggest that PNCAS‐Tat‐induced biogenesis of exosomes in MSCs is mediated by secretary autophagy, a mechanism that has recently been reported for viral propagation and for cellular response to stress [41, 42, 43]. Classical autophagy leads to degradation in lysosomes. Yet in recent years, a nonclassical autophagy pathway termed secretory autophagy, which leads to secretion (including EV secretion) rather than lysosomal degradation, has been described in many scenarios [41, 42, 43]. First, the expression levels of autophagy markers LC3B/LC3A and Beclin‐1 (both encoded by autophagy‐related genes, or ATGs) were raised in a time‐dependent manner with the incubation of PNCAS‐Tat with BMSCs (Figure 6A,B). Using an autophagy inhibitor 3‐MA to treat BMSCs in the presence of PNCAS‐Tat resulted in a significant reduction in the number of exosomes generated from the cells (Figure 6C). Second, in BMSCs, PNCAS‐Tat showed a low colocalization level (Manders colocalization coefficient < 0.2) with lysosomes stained by LysoTracker at both the 8 h time point (endocytosis phase) and the 24 h time point (exocytosis phase) (Figure 6D,E). Third, we studied the levels of Rab5 (regulator of formation of early endosome) and Rab7 (regulator of transport from late endosome to lysosome) in BMSCs with the incubation of PNCAS‐Tat [44]. The mRNA level of Rab5 was increased at the 8 h time point (endocytosis phase) compared with the zero‐time point, indicating increased activity of early endosome formation in BMSCs in response to PNCAS‐Tat (Figure 6F). Later, at the 24 h time point (exocytosis phase) the mRNA level of Rab5 was reduced (Figure 6F). Meanwhile, the mRNA level of Rab7 in BMSCs was reduced at the 8 h time point (endocytosis phase) and the 24 h time point (exocytosis phase) compared to the zero‐time point, with the incubation with PNCAS‐Tat, indicating suppression of transport to the lysosome (Figure 6F). Results of the protein levels of Rab5 and Rab7 (Figure 6G,H) showed the same trends as those of the mRNA levels (Figure 6F). Forth, we employed TEM to visualize the cellular transport process. As shown in Figure 6I,J, with the incubation of PNCAS‐Tat in BMSCs, we found a large increase (∼3‐fold) in the number of multivesicular bodies (MVBs) at the 8 h time point (endocytosis phase) which later dropped at the 24 h time point (exocytosis phase). This indicates that PNCAS‐Tat induced a large increase of formation of MVBs in BMSCs. It is known that MVBs are required for the formation of exosomes [1]; this observation thus offers a mechanistic insight into the stimulating effect of PNCAS‐Tat on exosome biogenesis in BMSCs. Meanwhile, we observed a large increase (∼7‐fold) in the number of autophagosomes at the 24 h time point (exocytosis phase) (Figure 6I,J). On the other hand, for the number of lysosomes, we detected a large reduction (∼2‐fold) at both the 8 h time point (endocytosis phase) and the 24 h time point (exocytosis phase) compared with the zero‐time point (Figure 6I,J).

In comparison, the TEM results of MCF‐7 cells revealed a clear difference from those of BMSCs. For MCF‐7 cells, we found no significant change in the number of MVBs at both the 8 and 24 h time points, only a ∼2‐fold increase (much lower than the ∼7‐fold increase for BMSCs) in the number of autophagosomes at the 24 h time point, and a large increase (∼3‐fold) in the number of lysosomes at the 24 h time point (Figure 6K,L). This indicates that, unlike BMSCs, for MCF‐7 cells the primary cellular transport pathway of PNCAS‐Tat does not involve secretary autophagy, but rather primarily involves the lysosomal pathway; and it does not induce increased MVB formation. To further study the cell type dependence of the secretary autophagy vs. lysosomal pathway competition, we used a convenient experimental method, namely LysoTracker colocalization. Adipose‐derived MSCs (AMSCs) showed a low colocalization with lysosomes (Manders colocalization coefficient < 0.2), a result similar to BMSCs (Figure S13). In stark contrast, in the 4 cancer cell lines examined (i.e., MCF‐7, HeLa, HepG2, and SH‐SY5Y), the results showed high colocalization levels with lysosomes (Manders colocalization coefficients 0.7–0.9) at the 24 h time point (Figure S13). Taken together, our results indicate that, in MSCs, the cellular transport of PNCAS‐Tat primarily goes through the secretary autophagy pathway, and stimulates a large amount of MVB formation and exosome biogenesis; in contrast, in cancer cell lines, the cellular transport of PNCAS‐Tat primarily goes through the endosome‐lysosome pathway, and stimulates no (or only a small amount of) MVB formation and exosome biogenesis. Figure 6M shows a schematic illustration of the biological pathways suggested by the mechanistic studies.

The main reason for the therapeutic capacity of exosomes is that they possess a large portion of the molecular contents, e.g., proteins, nucleic acids, and lipids, of the source cells [1]. Thus, we wanted to check to confirm that PNCAS‐Tat did not overly change the molecular contents of the source cells and exosomes. Using protein content as a representative of molecular contents in cells and exosomes, we performed proteomics analysis by liquid chromatography‐tandem mass spectrometry (LC‐MS/MS). Figure S14 shows the protein composition comparison of BMSCs after incubation with PNCAS‐Tat for 8 and 24 h, and BMSCs without PNCAS‐Tat incubation. The results demonstrate that incubation with PNCAS‐Tat did not change the vast majority of protein composition of BMSCs. Venn diagram reveals that, out of the ∼1900 detected proteins, 1416 of them (∼75%) were not qualified as differentially expressed proteins (DEPs) (fold change >1.5 and *p* value <0.05, Figure S14A). This pattern can also be seen from the volcano plots (Figure S14D). We then focused study on the DEPs. Gene Ontology (GO) analysis indicates upregulation of proteins involved in “wound healing,” “cellular response to stimuli,” and “extracellular vesicle,” etc. in BMSCs with PNCAS‐Tat incubation (Figure S14E). Kyoto Encyclopedia of Genes and Genomes (KEGG) pathway analysis shows upregulation of proteins involved in “motor proteins,” “exocytosis,” etc. (Figure S14F).

Figure S15 shows the protein composition comparison between PNCAS‐Tat‐induced exosomes from BMSCs and exosomes with starvation stimulation (conventional stimulation) from BMSCs. The results demonstrate that stimulation with PNCAS‐Tat did not change the vast majority of protein composition of exosomes from BMSCs. Venn diagram reveals that, out of the 831 detected proteins in the exosomes from BMSCs with starvation stimulation, 676 of them (81%) were also present (with similar concentrations) in the PNCAS‐Tat‐induced exosomes from BMSCs (Figure S15A). This pattern can also be seen from the volcano plots (Figure S15B). In addition, PNCAS‐Tat stimulation of BMSCs raised the total number of proteins in the exosomes (from 831 to 1061) (Figure S15A). Out of the 1862 detected proteins in BMSCs, exosomes with starvation stimulation contained 34.9% of them, while exosomes with PNCAS‐Tat stimulation increased this percentage value to 43.4% (Figure S15A). Further, GO analysis indicates enrichment of proteins involved in “wound healing,” “regeneration,” “response to hypoxia,” “extracellular vesicle,” etc. (Figure S15C). KEGG pathway analysis shows enrichment of proteins involved in “vesicle trafficking,” “motor proteins,” “endocytosis,” “autophagy,” “protein export,” “focal adhesion,” “ECM‐receptor interaction,” etc. (Figure S15D).

### 2.7 Applications of Chemically Engineered MSCs‐Derived Exosomes

The presence of PNCAS in the engineered MSCs‐derived exosomes offers the ability of imaging and tracking with multiple modalities. We demonstrate the following imaging modalities offered by SPIONs or/and QDs in the PNCAS encapsulated in exosomes: MRI, fluorescence imaging, and TEM. First, MRI can be applied to image the engineered MSCs‐derived exosomes in live animals. As shown in Figure S16A, T2*‐weighted MRI imaging of the brain of a live mouse at three different time points (0, 3, 24 h) after intravenous injection demonstrated successful delivery of engineered MSCs‐derived exosomes into the brain, after crossing the blood‐brain barrier (BBB). The MRI signal came from the SPIONs in the PNCAS‐encapsulated exosomes. Some of the engineered exosomes delivered to the brain by intravenous injection likely remained in the blood vessels without entering the brain parenchyma, and were later removed from the brain by neurovascular blood flows, as suggested by the observation that there were fewer MRI signals of SPIONs in the brain at 24 h compared with 3 h. The inherent capacity of exosomes to cross the BBB has been reported in a number of previous reports [4, 5].

Second, fluorescence microscopy can be used to image and track the engineered MSCs‐derived exosomes in live cells at the single‐particle level. Video S1 and Figure S16B show the movement of a PNCAS‐encapsulated vesicle (with green QDs), which was probably exiting the source cell (BMSC), as imaged by spinning‐disk confocal fluorescence microscopy. The lipid membranes of vesicles were labeled by a red fluorescent dye DiO. The microscope was equipped with two identical electron‐multiplying charge‐coupled device (EMCCD) cameras for simultaneous dual‐channel image acquisition (one channel for QD, the other for DiO). In the video, the PNCAS‐encapsulated vesicle was seen as a yellow particle, with green color from QD and red color from DiO (Video S1 and Figure S16B), in every frame in which the particle appeared. The combination of intense and stable fluorescence from QDs and simultaneous dual‐channel EMCCD‐based image acquisition permitted the unambiguous confirmation that the object being tracked was a PNCAS‐encapsulated vesicle. A sudden and large increase in mobility during the course of the trajectory indicated the probable occurrence of release to the extracellular space (Video S1 and Figure S16B). In addition, spinning‐disk confocal fluorescence microscopy was also used to image the uptake of PNCAS‐encapsulated exosomes into SH‐SY5Y cells (a neuroblastoma cell line) (Figure S16C). Third, as shown earlier in Figure 3I, TEM can be used to image the engineered MSCs‐derived exosomes below the diffraction limit of light, because the encapsulated PNCAS contain electron‐dense materials such as SPIONs and QDs.

We demonstrate the therapeutic applications of the engineered MSCs‐derived exosomes in neurological diseases. We first examined the brain delivery ability in vitro and in vivo. We employed an in vitro Transwell model of BBB, which contained HUVECs and astrocytes, to examine the ability of several different formulations to cross the BBB (Figure S16D). It was found that the PNCAS‐loaded exosomes formulation displayed a significantly higher BBB crossing ability at 24 and 48 h incubation times than PNCAS‐Tat, as measured by ICP‐OES (Figure S16E,F). Applying an external magnet to attract SPIONs led to further enhancement of BBB crossing (Figure S16E,F). By 48 h with magnet treatment, ∼40% of the PNCAS‐loaded exosomes had crossed the BBB in the Transwell model (Figure S16E,F). The same trend was found in vivo in mouse models. The different formulations were injected intravenously in mice. A magnet was placed on the mouse skulls for 3 h for assessing the effect of the magnetic attractive force on BBB crossing. At 48 h after the injection, all four formulations yielded significant brain delivery as shown by brain tissue imaging, with fluorescence of QDs or Prussian blue staining of SPIONs (Figure S16G–K). PNCAS‐loaded exosomes resulted in greater brain delivery than PNCAS‐Tat, and application of a magnet led to further delivery enhancement (Figure S16G–K).

We subsequently examined the ability of the engineered MSCs‐derived exosomes with CUR loading to treat Parkinson's disease (PD) in vitro and in vivo. The in vitro PD model used was 1‐methyl‐4‐phenylpyridinium (MPP)‐treated SH‐SY5Y cells. The engineered MSCs‐derived exosomes showed significant therapeutic effects with regard to increasing the level of antioxidative stress biomarker glutathione (GSH) and reducing the levels of oxidative stress biomarker malondialdehyde (MDA), reactive oxygen species (ROS), and apoptosis (Figure S17A–F). The therapeutic effect was found to be dose‐dependent, with the apoptosis‐reducing effect as an indicator (Figure S17G,H).

**FIGURE 7:**
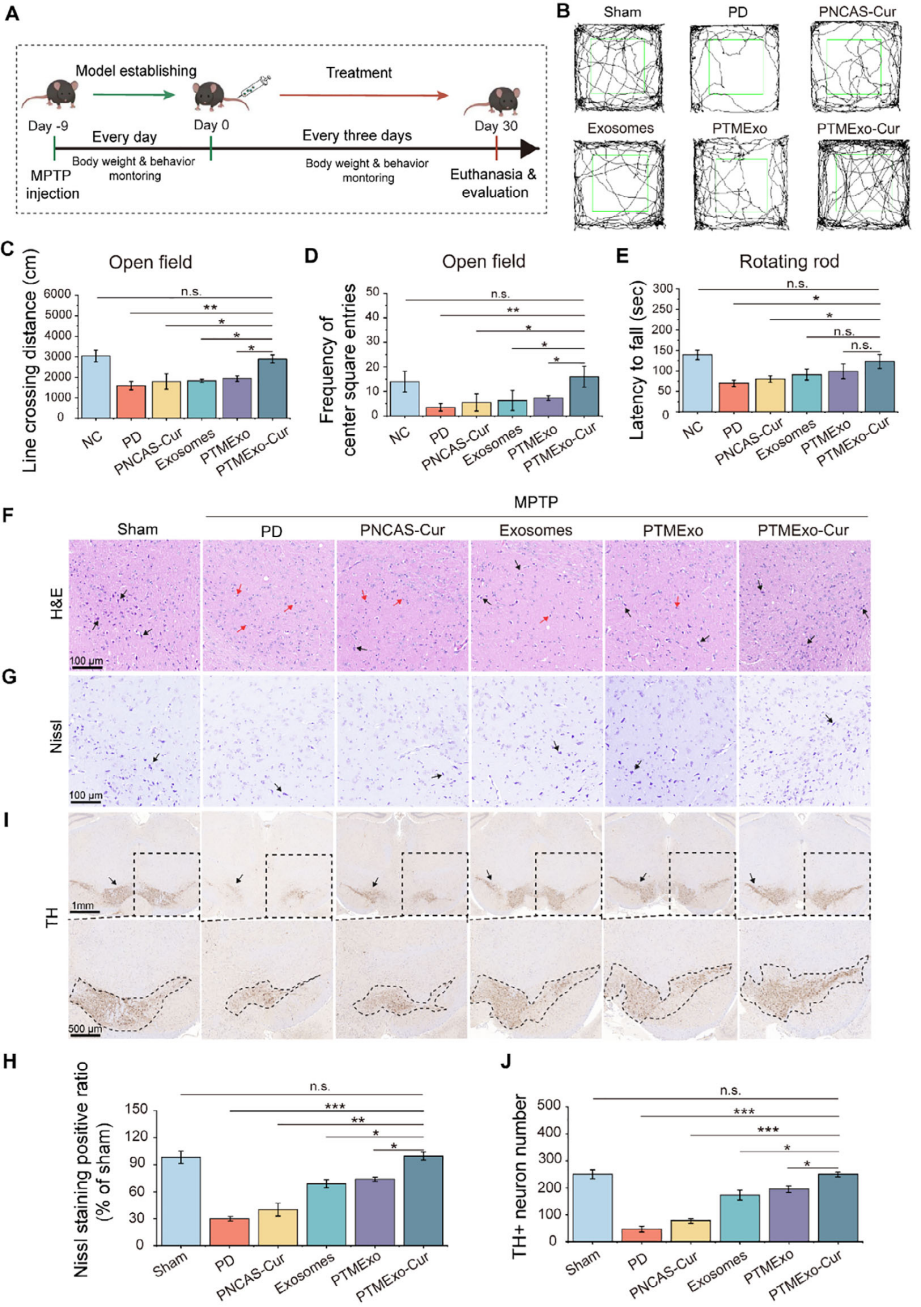
Applications in neurological disease: therapeutic efficacy in PD mice model. The different mice groups include Sham (normal mice control with PBS treatment), PD (PD mice with PBS treatment), PNCAS‐Cur (PD mice treated with CUR‐loaded PNCAS‐Tat), Exosomes (PD mice treated with MSCs‐derived exosomes without PNCAS or CUR), PTMExo (PD mice treated with PNCAS‐Tat‐encapsulated MSCs‐derived exosomes without CUR), and PTMExo‐Cur (PD mice treated with CUR‐loaded, PNCAS‐Tat‐encapsulated, MSCs‐derived exosomes). n = 5 mice in each group. (A) Schematic of experimental procedure. (B) Representative movement trajectories of mice in the open field behavioral analysis. (C,D) Quantification of (B). (E) Quantification results of rotating rod experiments. (F) H&E staining of the substantia nigra region of mouse brains. Black arrows: nucleus of normal neurons. Red arrows: condensed nucleus in diseased neurons. Scale bar: 100 µm. (G) Nissl staining of the substantia nigra region of mouse brains. Black arrows: Positive staining of Nissl bodies (rough endoplasmic reticulum and ribosomes) in neurons. Damaged or dying neurons may show loss of Nissl bodies. Scale bar: 100 µm. (H) Quantification of (G), the Nissl staining results. (I) Tyrosine hydroxylase (TH) immunohistochemistry of the substantia nigra region of mouse brains. Black arrows: TH‐positive dopaminergic neurons. Scale bar: 100 µm. (J) Quantification of TH‐positive dopaminergic neurons in the substantia nigra region of mouse brains. For (C,D,E,H,J), data are presented as mean ± SD and are analyzed with one‐way ANOVA followed by Tukey's multiple comparisons test. n.s., not significant; ^*^ *p* <0.05; ^**^ *p* <0.01; ^***^ *p* <0.001.

The in vivo PD model used was mice treated by the toxin 1‐methyl‐4‐phenyl‐1,2,3,6‐tetrahydropyridine (MPTP). PD mice were given treatment by tail vein injection of a formulation consisting of CUR‐loaded PNCAS‐Tat, or MSCs‐derived exosomes (without CUR or PNCAS), or PNCAS‐encapsulated MSCs‐derived exosomes (without CUR), or CUR‐loaded PNCAS‐encapsulated MSCs‐derived exosomes, with each injection containing 1 × 10^9^ particles/mouse given every 3 days for a total of 30 days (Figure 7A). PBS was injected to PD mice in the negative control sample. For those formulations with CUR, the CUR loading in PNCAS was 53%. For all treatments with tail vein injection, a magnet was placed on top of the mice skulls for 3 h for magnetism‐enhanced delivery. We utilized behavior analysis and neuron staining to evaluate the therapeutic outcome in mice. The results of these methods consistently showed that the engineered MSCs‐derived exosomes yielded a significant therapeutic efficacy on PD, and that the formulation of CUR‐loaded exosomes produced better therapeutic outcome than either the formulation of PNCAS‐loaded exosomes without CUR or the formulation of CUR‐loaded PNCAS‐Tat (Figure 7A–J). In the literature, MSCs‐derived exosomes have been reported to generate therapeutic efficacy on PD by regeneration and immunomodulation effects [1, 5]; on the other hand, the drug CUR has been reported to produce therapeutic efficacy on PD by anti‐inflammation and modulation of the aggregation and toxicity of α‐synuclein [37, 38, 39, 40]. Thus, our results suggest that CUR‐loaded MSCs‐derived exosomes could combine the therapeutic effects of MSCs‐derived exosomes and CUR, yielding improvement compared to the individual formulations. With the treatment of the best‐performing formulation (CUR‐loaded PNCAS‐encapsulated MSCs‐derived exosomes), the PD mice showed near complete recovery in terms of the results of behavior analysis and neuron staining (Figure 7B–J). It is worth noting that in some of the behavior experiment results (e.g., Figure 7D), the error bars are rather large. This is due to the high variability nature of animal behaviors linked to brain activity: the behavior of individual animals even in the same group can have large variation. This is why cellular experiments are needed to validate the conclusion of behavior experiments, as we have done here (Figure 7F–J).

**FIGURE 8:**
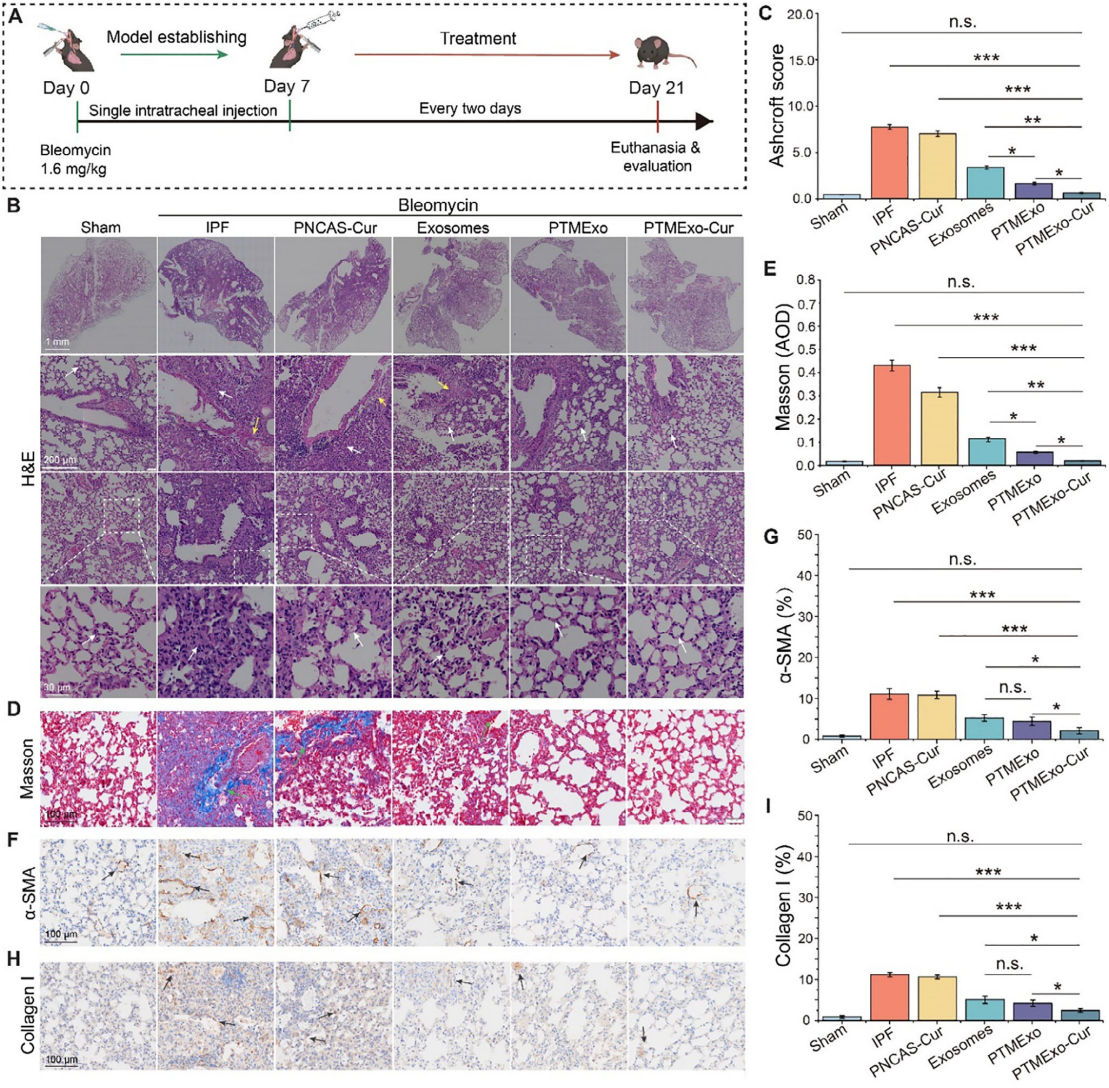
Application of engineered exosomes in treating idiopathic pulmonary fibrosis (IPF) in mice model. The different mice groups include Sham (normal mice control with PBS treatment), IPF (IPF mice with PBS treatment), PNCAS‐Cur (IPF mice treated with CUR‐loaded PNCAS‐Tat), Exosomes (IPF mice treated with MSCs‐derived exosomes without PNCAS or CUR), PTMExo (IPF mice treated with PNCAS‐Tat‐encapsulated MSCs‐derived exosomes without CUR), and PTMExo‐Cur (IPF mice treated with CUR‐loaded, PNCAS‐Tat‐encapsulated, MSCs‐derived exosomes). (A) Schematic of the experimental procedure. (B) H&E staining evaluation of lung tissues including areas near and far away from the bronchus. White arrows: alveolus; yellow arrows: bronchial fibrosis. (C) Quantification of Ashcroft scores of the H&E staining results. (D) Masson staining evaluation of lung tissues. Green arrows: blue stains of fibrotic tissues. Scale bar: 100 µm. (E) Quantification of the Masson staining results. AOD: average optical density. (F) immunohistochemistry of α‐SMA in lung tissues. Black arrows: α‐SMA‐positive cells. Scale bar: 100 µm. (G) Quantification of the results of α‐SMA immunohistochemistry. (H) immunohistochemistry of Collagen I in lung tissues. Black arrows: Collagen I‐positive cells. Scale bar: 100 µm. (I) Quantification of the results of Collagen I immunohistochemistry. Data are presented as mean ± SD (*n* = 5 mice) and analyzed with one‐way ANOVA followed by Tukey's multiple comparisons test. n.s., not significant; ^*^ *p* <0.05; ^**^ *p* <0.01; ^***^ *p* <0.001.

We demonstrate the therapeutic applications of the engineered MSCs‐derived exosomes in a respiratory disease. We examined the ability of the engineered MSCs‐derived exosomes with CUR loading to treat idiopathic pulmonary fibrosis (IPF) in a mouse model, which was induced by a single, high‐dose, intratracheal injection of bleomycin. At day 7, by which point lung fibrosis had started, the mice were given inhalation treatment using a nebulizer every 2 for 14 days with a formulation of CUR‐loaded PNCAS‐Tat‐encapsulated MSCs‐derived exosomes, or CUR‐loaded PNCAS‐Tat, or MSCs‐derived exosomes (without CUR and PNCAS), or PNCAS‐Tat‐encapsulated MSCs‐derived exosomes (without CUR), or PBS (equal volume), with each dose containing 1 × 10^9^ particles (except the PBS formulation) (Figure 8A). For those formulations with CUR, the CUR loading in PNCAS was 53%. We assessed fibrosis extent and collagen deposition of the lung tissues by haematoxylin and eosin (H&E) staining, Masson staining, and immunostaining of α‐SMA and collagen I. The results suggested that the formulation of CUR‐loaded MSCs‐derived exosomes produced better therapeutic efficacy than either the formulation of MSCs‐derived exosomes (without CUR) or the formulation of CUR‐loaded PNCAS‐Tat, with regard to reduction of fibrosis and collagen deposition, thus supporting the therapeutic value of combining exosomes and CUR (Figure 8B–I). Prussian blue staining showed that, after inhalation, the engineered exosomes were able to reach both bronchus and alveoli (Figure S18). Interestingly, PNCAS‐Tat‐encapsulated MSCs‐derived exosomes without CUR appeared to yield better therapeutic efficacy than MSCs‐derived exosomes without PNCAS‐Tat (statistically significant for the Ashcroft score and Masson staining, not statistically significant for immunostaining of α‐SMA and collagen I, Figure 8B–I). This could be explained by the improved exosome stability against the stress condition in inhalation offered by the PNCAS encapsulation in exosomes (Figure 5F–H). An alternative explanation could be the increased number of bioactive molecules in the MSCs‐derived exosomes stimulated by PNCAS‐Tat, as indicated by the omics analysis (Figure S15). Finally, the best‐performing formulation (CUR‐loaded PNCAS‐Tat‐encapsulated MSCs‐derived exosomes) reversed the lung fibrosis and collagen deposition close to the level of healthy mice (Figure 8B–I).

We examined the therapeutic efficacy of the engineered MSCs‐derived exosomes in a skin wound healing model in mice. A wound (0.6 cm) was induced by cutting the back skin of a mouse. The mice were given treatment every 3 days by subcutaneous injection of a formulation (each injection 1 × 10^9^ particles, except the Sham group) of CUR‐loaded PNCAS‐Tat‐encapsulated MSCs‐derived exosomes, or CUR‐loaded PNCAS‐Tat, or MSCs‐derived exosomes (without CUR or PNCAS‐Tat), or PNCAS‐Tat‐encapsulated MSCs‐derived exosomes (without CUR), or saline (equal volume) (Figure S19A). For those formulations with CUR, the CUR loading in PNCAS was 53%. The therapeutic efficacy was evaluated by naked eye inspection (for the wound closure rate), H&E staining (for the scar length), Masson staining (for the collagen content), and immunostaining of CD31 (angiogenesis marker). The results indicated that the formulation of CUR‐loaded PNCAS‐Tat‐encapsulated MSCs‐derived exosomes yielded better wound healing outcome than either the formulation of MSCs‐derived exosomes (without CUR or PNCAS‐Tat) or the formulation of CUR‐loaded PNCAS‐Tat, with regard to scar length, collagen content, and new blood vessel formation, thus supporting the therapeutic value of combining exosomes and CUR (Figure S19B–I).

We examined the therapeutic efficacy of engineered MSCs‐derived exosomes in an in vitro model of ischemic heart disease. The in vitro model was established by treating H9C2 cells (cardiomyocytes) under hypoxia stress. The therapeutic efficacy was analyzed by cell viability, ROS level, and mitochondria activity. Compared with no treatment, both engineered MSCs‐derived exosomes (with PNCAS‐Tat) and MSCs‐derived exosomes (without PNCAS‐Tat) resulted in high therapeutic efficacy, as indicated by increased cell viability, reduced ROS level, and increased mitochondria activity (Figure S20). The formulation of PNCAS‐Tat‐encapsulated exosomes exhibited a similar or higher level of efficacy compared with the formulation of exosomes without PNCAS‐Tat (Figure S20).

We examined the therapeutic efficacy of engineered MSCs‐derived exosomes in an in vitro model of polycystic ovary syndrome (PCOS), a female reproductive system disease. An in vitro PCOS model was established by treating KGN cells with dehydroepiandrosterone (DHEA), which stimulates inflammation and oxidative stress. The therapeutic efficacy was analyzed by cell viability and ROS level. Compared with no treatment, both engineered MSCs‐derived exosomes (with PNCAS‐Tat) and MSCs‐derived exosomes (without PNCAS‐Tat) resulted in high therapeutic efficacy, as indicated by increased cell viability and reduced ROS level (Figure S21). The formulation of PNCAS‐Tat‐encapsulated exosomes exhibited a similar or higher level of efficacy compared with the formulation of exosomes without PNCAS‐Tat (Figure S21).

We examined the biodistribution of engineered exosomes (PNCAS‐Tat‐encapsulated MSCs‐derived exosomes) among major organs of healthy mice 24 h after intravenous injection (each injection containing 1 × 10^9^ particles). The results indicated that the highest percentage of engineered exosomes was in the spleen, with the liver also taking a large portion (Figure S22A). Notably, a significant amount of the engineered exosomes was delivered to the brains of healthy mice which, unlike PD mice, had intact BBB (Figure S22A). Relative to the injected amount of engineered exosomes, ∼4% was delivered to the brains of healthy mice (Figure S21B). With a magnet placed on top of the mice skulls, the percentage of brain delivery relative to the injected amount was increased to ∼9% (Figure S22B).

We performed biocompatibility tests on the engineered exosomes (PNCAS‐Tat‐encapsulated MSCs‐derived exosomes). We introduced a high dose of engineered exosomes into healthy mice by intravenous injection (1 × 10^10^ particles/mouse). After 24 h, whole blood was collected, and biomarkers were assessed. We found that the injection of high dose of engineered exosomes caused no significant changes to the levels of biomarkers of liver function (aspartate aminotransferase, i.e., AST, alanine aminotransferase, i.e., ALT, alkaline phosphatase, i.e., ALP, lactic dehydrogenase, i.e., LDH, and total bilirubin) (Figure S23A–E). Similarly, there were no significant changes to the levels of biomarkers of kidney function (uric acid, urea, and creatinine) (Figure S23F–H). No significant changes were detected on the levels of immunogenicity biomarkers (white blood cell, neutrophil, lymphocyte, and monocyte) (Figure S23I–L). Moreover, in PD mice, after 10 injections of the formulation of engineered MSCs‐derived exosomes (with CUR and PNCAS‐Tat, each injection containing 1 × 10^9^ particles/mouse, one injection given every 3 days for a total of 30 days), no significant changes were observed to the tissue morphology of heart, liver, spleen, lung, and kidney (Figure S23M). We also evaluated the neurotoxicity of engineered exosomes (PNCAS‐Tat‐encapsulated MSCs‐derived exosomes) in healthy mice, considering the potential risk of iron‐induced neurotoxicity caused by the presence of iron in the engineered exosomes and the ability of exosomes to enter the brains. We introduced engineered exosomes (PNCAS‐Tat‐encapsulated MSCs‐derived exosomes) to healthy mice at an equivalent dose level as therapy (1 × 10^10^ particles/mouse), and performed behavior analysis, brain tissue morphology analysis, and neuronal analysis after 30 days. The results indicate no significant changes in these measures (Figure S24), suggesting that at the therapeutic dose level, the engineered exosomes pose no significant risk of neurotoxicity. Taken together, the results of the above tests showed excellent biocompatibility of the engineered exosomes.

## 3 Discussion

Manufacture remains a bottleneck issue for cell therapies in general, and for EV therapies in particular [45]. In the present work, we have developed an integrated technology Tat‐PNCAS‐MIMS‐MSC‐Exo to address the manufacturing challenge of chemically engineered MSCs‐derived exosomes. By integrating three innovative core components, including a novel biology component (Tat‐MSC‐Exo), a novel physics component (MIMS), and a novel material component (PNCAS), the Tat‐PNCAS‐MIMS‐MSC‐Exo technology can greatly improve every stage of the manufacturing flow of chemically engineered MSCs‐derived exosomes, i.e., biogenesis, drug and nanoparticle loading, isolation, and storage. This technology enables a ∼18‐fold improvement in the number of exosomes generated per source cell (to 3.5 × 10^5^ exosomes/cell), compared to the commonly‐used stimulation method of exosome biogenesis (starvation treatment) (Figure 2F). It should be mentioned that, in addition to the starvation treatment, in the literature a number of other methods have been reported to stimulate the exosome secretion from a source cell [46, 47, 48, 49, 50, 51, 52, 53, 54]. These methods could be broadly categorized as genetic methods, physical methods, and chemical methods. The genetic methods incorporate ‘booster genes,’ i.e., genes that can enhance exosome secretion, in the source cells [46]. The physical methods employ physical stimuli, such as mechanics, acoustics, or electricity, to increase the exosome secretion [47, 48, 49]. A common limitation of the physical methods is the difficulty to deliver identical stimuli to individual cells, leading to poor controllability and reproducibility of production. A recent paper by Wang et al. reported an innovative solution to address this limitation by using a microfluidics platform [50]. The chemical methods utilize chemical stimuli, such as hypoxia, low pH, or certain molecules, to enhance the exosome secretion [51, 52, 53, 54]. Our PNCAS‐Tat‐mediated stimulation can be considered as a type of chemical methods. The purity of the product (engineered exosomes) can reach ∼90% in three aspects (Figure s3; Figure 2G). The ∼10% impurities in the product primarily contain (1) non‐exosome EVs (such as ectosomes) that are nonspecifically adsorbed to drug‐loaded PNCAS‐Tat, and (2) drug‐loaded PNCAS‐Tat not encapsulated in EVs. On the one hand, these impurities could offer therapeutic benefits, because they contain molecular contents with therapeutic capacities (ectosomes contain therapeutic molecules from the source cells; drug‐loaded PNCAS‐Tat contain drug). On the other hand, these impurities pose little extra safety risk, because they contain little to no content that the pure products do not have. Nevertheless, it is necessary to keep the quantity of these impurities to be at a low level, in order to offer consistent pharmacokinetics and pharmacodynamics. The entire manufacturing process is remarkably robust. We have achieved excellent reproducibility of the product properties (Figure S11). We have also achieved good contamination control (Figure S12). Efforts to build a Good Manufacturing Process (GMP)‐like quality management system are underway. The source cells used in the current manufacturing process are MSCs. In future studies, for another given type of source cells, a high‐throughput screening, potentially in conjunction with artificial intelligence, would be conducted to identify the optimal peptide sequence.

MIMS enables rapid, efficient, and scalable isolation of PNCAS‐loaded exosomes with low cost. Over 95% of the PNCAS‐loaded exosomes in the dispersion can be isolated within 1 h (Figure 4F). The performance is due to the unique design of MIMS, which provides a high magnetic field gradient (thus high magnetic force) in an automatic fashion to attract magnetic objects at every location in the dispersion. The MIMS design also offers a crucial advantage for scale‐up: the isolation times needed to achieve near‐complete isolation are nearly the same for larger scales as for smaller scales, using a larger number of magnet units in larger‐scale isolation. Additionally, MIMS circumvents the common problems encountered in magnetic column, tangential flow filtration, and microfluidics. e.g., clogging of channels or pores [55]. Scale‐up is a long‐standing problem in applying magnetic isolation for biomanufacturing, although it has been widely employed for biological detection and analysis which typically uses small sample volumes [31]. Magnetic isolation is currently not yet a common method in industry for isolating therapeutic exosomes despite its advantages in low cost, easy operation, and high specificity. MIMS (especially coupled with PNCAS) would enable the realization of the full potential of magnetic isolation for therapeutic exosomes.

The current yield of our manufacturing process can reach ∼5 × 10^12^ engineered exosomes from each production cycle. This level of scale is sufficient for animal experiments and phase 1 clinical trials [3, 4, 14]. For phase II, III clinical trials and commercial mass production, scale‐up is needed, primarily in magnetic isolation, PNCAS fabrication, and cell culture. The scale‐up of magnetic isolation is expected to benefit from the MIMS design, as discussed above. For PNCAS fabrication scale‐up, as reported in our previous publication, preliminary studies indicate that the fabrication can be scaled up without significant loss of qualities [36]. For cell culture scale‐up, a large‐scale cell culture method (e.g., multilayered culture flasks, hollow fiber cartridges, or bioreactors) well‐established in industry can be used [56]. The manufacturing process of engineered exosomes is rather economical, because the costs of all the raw materials, devices, and processing steps are fairly low. It is worth noting that, in the extracellular medium after PNCAS‐Tat stimulation of MSCs, there is a 60%–70% exosome population that does not have PNCAS loaded (Figure 3K). This population could also be isolated by MIMS using PNCAS conjugated with anti‐CD63 antibody or another exosome‐recognizing molecule, and be used for therapeutic applications. In addition to exosome isolation, MIMS could find broader applications in magnetic isolation of cells and biomolecules, especially given its advantage in scale‐up (Figure S10).

The Tat‐PNCAS‐MIMS‐MSC‐Exo technology is built upon our finding of a new biological phenomenon, i.e., with MSCs, Tat‐coated nanoparticles can stimulate their endocytosis, MVB generation, secretory autophagy, and exosome biogenesis (Figure 6). This phenomenon might be a consequence of the Tat peptide being derived from the HIV virus and/or a cellular response to stress. Secretory autophagy is an emerging field in cell biology [41, 42, 43]. Recently, there have been a few publications in the literature reporting that incubating with nanoparticles led to secretory autophagy in cells [57]. Compared with these reports, the present work has advanced the knowledge of secretory autophagy biology in the following ways. First, we have found that, in MSCs, Tat‐coated nanoparticles can result in not only secretory autophagy, but also an increased number of MVBs, thereby yielding a higher number of exosomes. Second, we have found a clear cell type dependence of this biological phenomenon. In cancer cell lines (e.g., MCF‐7, HeLa, HepG2, SH‐SY5Y, and HXO‐RB44), although this biological phenomenon does occur to some extent, the degree is much lower than that in MSCs (e.g., BMSCs and AMSCs). It is worth noting that we recently reported a distinct difference in the endocytosis mechanism of Tat‐conjugated QDs between BMSCs and HeLa cells, i.e., the former uses caveolae‐mediated endocytosis while the latter does macropinocytosis [34]. The striking differences in nanoparticle‐cell interactions between MSCs and cancer cell lines deserve further studies. Third, we have found that Tat‐coated nanoparticles can stimulate more exosome secretion than free Tat peptide, that is, amplifying the stimulation effect of Tat peptide. This previously unknown nano‐effect could potentially serve as a general design principle for the stimulation of exosome secretion. There could be several possible mechanisms for this amplification effect: (1) Cooperative binding. Multiple Tat molecules on a nanoparticle could generate a multivalence effect in binding with the cell membrane, producing greater binding affinity; (2) Protective effect. Nanoparticles could protect Tat molecules from degradation; (3) Synergistic effect. The individual stimulating effects of Tat molecules and nanoparticles could in some way enhance each other. A further intriguing question is what role the secretary autophagy process plays in this amplification effect. Future work will investigate these mechanistic questions and generalize this nano‐effect with more types of stimulating molecules, nanoparticles, and cells. Fourth, we have quantified the key sorting ratios in the trafficking of PNCAS‐Tat in BMSCs, and the results are summarized as follows. (1) The number ratio of exocytosed PNCAS‐Tat to endocytosed PNCAS‐Tat is ∼75%. (2) The number ratio of PNCAS‐Tat encapsulated in exosomes to all exocytosed PNCAS‐Tat is ∼95%. (3) The number ratio of exosomes with encapsulated PNCAS‐Tat to all exosomes can be increased by raising the number of Tat peptides on each PNCAS to increase the endocytosis of PNCAS‐Tat, until it reaches the maximum ratio ∼40%. (4) The number ratio of exosomes with PNCAS‐Tat to EVs with PNCAS‐Tat is ∼92%. (5) In each exosome the encapsulated number of PNCAS‐Tat is 1 or 2 (on average 1.5). These ratios are robust, indicating a tightly regulated biological process.

Loading drugs or/and nanoparticles to EVs has been a challenge for the field of engineered EVs with regard to loading amount, efficiency, precision control, as well as EV stability. We have achieved excellent loading amount, efficiency, and precision control for both drugs and nanoparticles in our engineered exosomes. This is due to the excellent loading of drugs and nanoparticles in PNCAS, the ideal size of PNCAS, the excellent structural and colloidal stability of PNCAS‐Tat, and the robust biological process of Tat‐induced exosome biogenesis in MSCs. The loading capacity of drugs, with CUR as the model drug, reaches the level of 1 million drug molecules in each product EV (Figure 3F; Note S1), representing a multi‐fold improvement compared to the previous methods [20]. Crucially, in our engineered EVs, loading a large amount of drug molecules into EVs does not destabilize the EVs. This is because the drug molecules are pre‐loaded in the water‐soluble PNCAS and are thus not in the vesicle membranes. Table S1 provides a comparative summary of different loading methods for EV engineering. The reason why PNCAS offers high loading of drugs and nanoparticles, as well as excellent stability, could be cooperative hydrophobic forces between albumin, nanoparticles, and drugs in PNCAS [35, 36]. In a PNCAS, hydrophobic nanoparticles could be considered as numerous small hydrophobic units connected together, offering a stable scaffold and the source of cooperative hydrophobic forces with the hydrophobic regions of albumin and hydrophobic drugs [35, 36]. The types of nanoparticles and drugs co‐encapsulated into PNCAS can be extended beyond SPIONs/QDs and CUR. In principle, any nanoparticles with a hydrophobic surface and hydrophobic drugs are suitable for co‐encapsulation by PNCAS. Studies are ongoing to load nucleic acids together with hydrophobic drugs into PNCAS, using a modified version of PNCAS. It is possible to combine the presented technology with genetic engineering techniques of MSCs in the literature [58, 59, 60] to achieve co‐loading of nucleic acids (or proteins) together with drugs and nanoparticles in MSCs‐derived exosomes. For example, a miRNA could be co‐encapsulated into PNCAS together with a hydrophobic drug, and after the PNCAS‐Tat‐stimulated exosome biogenesis, both the miRNA and the drug would be effectively loaded into the exosome. Alternatively, a DNA plasmid, which expresses a fusion protein including a tissue‐targeting protein and an exosome membrane protein, could be co‐encapsulated into PNCAS together with a hydrophobic drug. This approach would yield exosomes with both the therapeutic function of the drug and the tissue‐targeting function of the targeting protein.

Stability of EVs is often a concern not only for storage but for the processes involving stressful treatments of EVs, e.g., filtration, injection, and inhalation. Here, we have used freeze‐thaw cycle (for storage) and inhalation (for delivery) as examples to demonstrate that our engineered exosomes have a built‐in mechanism of stability enhancement offered by the encapsulated PNCAS, which is structurally stable and mechanically much more rigid than native exosomes. This mechanism is analogous to that of carbon fiber‐enhanced plastics, which are commonly used in aerospace and sports equipment. In a related effort, a recent publication reported increasing the cholesterol content in the exosome membranes to enhance the stability of exosomes [61]. The stability of our engineered exosomes is sufficient for use as off‐the‐shelf products, as suggested by the observation that they maintain the number of particles in the size range of exosomes after freeze‐drying and re‐dispersion. It is possible to combine with other cryoprotectants to further improve the product stability in storage. It is worth noting that our engineered EVs have achieved the rare combination of both exceptionally high drug loading and excellent EV stability.

EVs are known for their exceptional ability to cross delivery barriers, including membranes (e.g., BBB and skin) and hydrogels (e.g., extracellular matrix) [4, 5, 6, 14]. The exceptional barrier‐crossing ability of EVs coupled with the ultrahigh drug loading ability of PNCAS in our technology provides a powerful combination for drug delivery. As reviewed by Terstappen et al., the BBB‐crossing formulations currently in clinical trials or under preclinical development (e.g., exosomes, nanoparticles, and cell‐penetrating peptides) are often limited by their low drug loading [62]. Our technology thus offers a solution to this limitation. The delivery capacity of our technology is further enhanced by magnet‐guided delivery, which is enabled by the SPIONs in our engineered exosomes. Magnet‐guided delivery can not only improve the barrier‐crossing ability but enable targeted delivery to a specific location (e.g., the brain, lung) (Figure S22). Clinical trials using magnet‐guided drug delivery have been previously reported [63, 64], suggesting that it is safe for clinical use. The three‐in‐one combination of drug delivery capacities, i.e., ultrahigh drug loading, exceptional barrier‐crossing, and built‐in targeting, can be extended to various drugs and diseases.

The ability to image a therapeutic entity is important for understanding its behaviors. EV therapies often suffer from the difficulty to image and track the EVs in biological environments [65]. Here utilizing the nanoparticles encapsulated in exosomes as the imaging probes, we have demonstrated the ability of multimodal imaging, including MRI in live animals, single‐particle fluorescence tracking in live cells, and TEM. The imaging capacity can be extended to other modalities, such as X‐ray imaging, ultrasound imaging, and super‐resolution microscopy, by using other types of nanoparticles or small molecule probes for encapsulation into PNCAS.

We have shown the therapeutic efficacy of our engineered exosomes in multiple disease models, including PD (in vivo and in vitro), IPF (in vivo), skin wound healing (in vivo), heart failure (in vitro), and PCOS (in vitro), thus demonstrating the broad applicability of the engineered exosomes. In these experiments, the therapeutic efficacies of PNCAS‐encapsulated MSCs‐derived exosomes (without drug) have been demonstrated in all the above disease models; moreover, the therapeutic benefits of combining exosome and drug have been shown in PD, IPF, and skin wound healing. In these experiments, CUR has been used as the model drug. In future studies, for each disease the optimal choice of drug needs to be identified. Furthermore, more detailed research on the therapeutic behaviors and mechanisms of the engineered exosomes are needed in future studies.

EVs are believed to be highly biocompatible in general [1, 3, 4, 5, 6]. The PNCAS in our engineered exosomes are typically composed of serum albumin and iron oxide nanoparticles, both with a long history of safe clinical uses [35]. We have demonstrated the biocompatibility of our engineered exosomes by blood tests, tissue morphology studies, and behavior analysis. More detailed biocompatibility studies are needed in future work. In particular, the potential neurotoxicity and cardiotoxicity of iron oxide nanoparticles due to nanozyme activity will be studied in more detail. It is worth noting that Fe_3_O_4_ nanozymes could exhibit different nanozyme activities at different environments. It was previously reported that these nanozymes exhibit peroxidase (POD)‐like activity (catalyzing H_2_O_2_ to produce •OH and H_2_O) at an acidic pH environment, and catalase (CAT)‐like activity (catalyzing H_2_O_2_ to produce O_2_ and H_2_O) at a neural pH environment [66]. The former (POD‐like activity) could cause toxicity by generating reactive oxygen species (ROS), while the latter (CAT‐like activity) could offer therapeutic efficacy by scavenging ROS. With the doses, time durations, and analytical methods used in our therapeutic studies, no neurotoxicity and cardiotoxicity from the engineered exosomes have been detected. Some previous reports from other research groups have also found no neurotoxicity and cardiotoxicity from iron oxide nanoparticles [67, 68]. If neurotoxicity or cardiotoxicity is indeed found in more detailed studies, it will be minimized by reducing the number of SPIONs encapsulated in each PNCAS.

In conclusion, we have developed a nanoparticle‐integrated technology Tat‐PNCAS‐MIMS‐MSC‐Exo to improve all four steps of the manufacture flow of chemically engineered MSCs‐derived exosomes, with large performance improvements compared to simple combinations of existing techniques. The scalability, robustness, and controllability of the manufacture process as well as the biocompatibility of the product offer significant translation potential in this technology. The capacity of loading large quantities of drugs and nanoparticles in an exosome with exceptional stability opens up new avenues in combinational therapy, drug delivery, and theranostics. The findings of mechanistic studies present new insights into the interplays among endocytosis, secretory autophagy, and exosome biogenesis. The studies on applications and mechanisms in the present work are preliminary in nature. Future work will further pursue the applications in specific diseases and examine the mechanisms of nano‐bio interactions systematically.

## 4 Methods

### 4.1 Materials

Cadmium oxide (CdO, 99.99%), zinc nitrate hexahydrate [Zn(NO_3_)_2_·6H_2_O, 98%], sulfur powder (S, 99.98%), selenium powder (Se, 100 mesh, 99.5%), 1‐octadecene (90%), stearic acid (95%), trioctylphosphine (TOP, 90%), cysteamine (98%), Fe(acac)_3_, oleylamine, oleic acid, hexadecanediol, benzyl ether, hexane, ethanol, N,N‐dimethylformamide (DMF), dichloromethane (DCM), triethylamine (TEA, 4%), bovine serum albumin (BSA), curcumin (CUR), and N‐succinimidyl 4‐(maleimidome.)cyclo‐hexanecarboxylate (SMCC) were purchased from Sigma–Aldrich (USA). BSA (sulfhydryl blocked, ≥98%) was purchased from Aladdin Scientific (USA). Tat peptide (sequence C‐YGRKKRRQRRR) was purchased from ChinaPeptides (Shanghai, China). Paraformaldehyde (PFA) was purchased from Yuanye BioTECH (Shanghai, China). THF (tetrahydrofuran) was purchased from Sinopharm Chemical Reagent (Shanghai, China). 3‐Methyladenine (3‐MA) was purchased from Shanghai BaiLi Biotechnology (Shanghai, China). MagDot 580 Anti‐Human CD 45+ conjugate was acquired from Core Quantum Technologies (USA). DiR and LysoTracker were purchased from KeyGEN BioTECH (Nanjing, China). Hoechst 33342 and DAPI were purchased from Thermo Fisher (USA).

The information about the antibodies used is shown below. Anti‐CD63 Mouse mAb: Servicebio; GB12620‐100; Research Resource Identifier (RRID): AB_3713241; 1:500 dilution). Anti‐TSG101 Mouse mAb: Servicebio; GB15618‐100; Research Resource Identifier (RRID): AB_3717500; 1:500 dilution. Anti‐CD81 Rabbit pAb: Servicebio; GB111073‐100; Research Resource Identifier (RRID): AB_3717501; 1:500 dilution. Anti‐ Rab 5 Rabbit mAb: Cell Signaling Technology; #3547; Research Resource Identifier (RRID): AB_2300649; 1:500 dilution. Anti‐ Rab 7 Rabbit mAb: Cell Signaling Technology; #9367; Research Resource Identifier (RRID): AB_1904103; 1:500 dilution. Anti‐ LC 3A/B Rabbit Rabbit: Abcam; Research Resource Identifier (RRID): AB_62721; 1:300 dilution. Anti‐Beclin‐1 Rabbit pAb: Servicebio; GB112053‐100; Research Resource Identifier (RRID): AB_3713242; 1:500 dilution. Anti‐β‐actin Rabbit mAb: Abcam; Research Resource Identifier (RRID): AB_179467; 1:800 dilution. Anti‐TH Rabbit mAb: Abcam; Research Resource Identifier (RRID): AB_137869; 1:500 dilution. Anti‐CD31 Rabbit pAb: Abcam; Research Resource Identifier (RRID): AB_28364; 1:50 dilution. Anti‐Collagen I Rabbit pAb: Abcam; Research Resource Identifier (RRID): AB_270993; 1:500 dilution. Anti‐α‐SMA Rabbit pAb: Abcam; Research Resource Identifier (RRID): AB_5694; 1:200 dilution.

The information about the cell lines used is shown below. Jurkat cell line: Fuheng #FH0878; Research Resource Identifier (RRID): CVCL_0065; Purchased in March 2024. MCF‐7 cell line: Fuheng #FH0215; RRID: CVCL_0031; Purchased in October 2022. SH‐SY5Y cell line: Fuheng #FH0156; RRID: CVCL_0019; Purchased in October 2022. H9C2 cell line: Fuheng #FH1004; RRID: CVCL_0286; Purchased in June 2023. KGN cell line: Fuheng #FH1125; RRID: CVCL_0375; Purchased in March 2023. HeLa cell line: Fuheng #FH0314; RRID: CVCL_0030; Purchased in October 2022. HepG2 cell line: Fuheng #FH0076; RRID: CVCL_0027; Purchased in October 2022. HUVEC cell line: Fuheng #FH1122; RRID: CVCL_2959; Purchased in September 2024. The cell lines were routinely tested and were mycoplasma‐free. The cell culture reagents were purchased from Biosharp Life Sciences (Hefei, China).

Sprague–Dawley (SD) rats, C57BL/J6 mice, and Kunming mice were acquired from Genepharma Biotechnology (Suzhou, China). Animal maintenance and experimentation were performed according to protocols approved by the Institutional Animal Care and Use Committee (IACUC) of the Experimental Animal Center at the Medical Center of Soochow University (the Fourth Affiliated Hospital of Soochow University, Suzhou Du‐Shu Lake Hospital) (Approval No. 2024‐241141).

### 4.2 Cell Culture

BMSCs were extracted from 6‐week‐old SD rats. After euthanasia by CO_2_, the whole femur and tibia were obtained and kept in a dish containing PBS with 1% penicillin‐streptomycin (PS). After the bone tissue was washed with PBS three times, the marrow cavity was repeatedly scoured by injecting Dulbecco's modified Eagle's medium (DMEM)/F12 medium with 10% fetal bovine serum (FBS) and 1% PS to leach out cells inside. The cells were collected by centrifugation at 1000 rpm for 5 min. The erythrocytes were then eliminated by Red Blood Cell Lysis Buffer (medium: buffer = 1:3). After centrifugation at 1000 rpm for another 5 min, the cells were re‐suspended and cultured in a 37°C incubator with 5% CO_2_. Half of the medium was abandoned and replaced by fresh medium after 24 h. The cell medium was refreshed every 3 days and the cells were passaged every week. The identity of MSCs was confirmed by their morphological features and molecular markers (CD90+, CD44+, CD45−). Cells between passage 3 and passage 5 were used. Cells with passage numbers outside this range had much less features of MSCs.

MCF‐7 cells (breast cancer cell line), HeLa cells (cervical cancer cell line), HepG2 cells (liver cancer cell line), SH‐SY5Y cells (neuroblastoma cell line), and human umbilical vein endothelial cells (HUVECs) were cultured in high glucose DMEM with 10% FBS and 1% PS, and placed in a 37°C incubator with 5% CO_2_. H9C2 cells (rat cardiomyocytes) and KGN cells (human ovarian granulosa cell line) were cultured in DMEM/F12 medium with 10% FBS and 1% PS, and placed in a 37°C incubator with 5% CO_2_.

Astrocytes were extracted from 1‐day newborn SD rats. The rats were euthanized by being placed in a CO_2_ environment and then treated with 75% ethanol for sterilization. After decapitating and opening the skull with surgical scissors, the rat brains were gently obtained and washed with PBS containing 5% PS three times to scour off the blood. The meninges were carefully removed using ophthalmic tweezers under an optical microscope; the brain tissue was then moved to a 15 mL centrifuge tube, and was pipetted several times until being dissolved after adding 0.25% trypsin (3–5 mL). The centrifuge tube was capped and placed in a 37°C incubator for 5 min. Tissue digestion was stopped by adding an equal volume of culture medium (DMEM/F12 + 10% FBS + 1% PS + 25 µg/mL transferrin + 10 nM biotin + 30 nM sodium selenate + 1 µg/mL butane diamine + 5 µg/mL insulin + 20 nM hydrocortisone + 20 nM progesterone + 1% bFGF). Finally, cells were seeded in cell culture bottles after being centrifuged for 5 min at 1000 rpm. The medium was exchanged 3 days later when cell adherence was confirmed. High purity of astrocytes was obtained by shaking the cell culture bottles for 2 h at 200 rpm on the fourth day of cell culture to reduce the number of microglial cells and oligodendrocytes. The supernatant was removed. The remaining cells were cultured to the third passage for the ensuing experiments.

### 4.3 Preparation and Characterizations of PNCAS and Drug (CUR)‐Loaded PNCAS

The following methods are adopted from reference 36: synthesis of hydrophobic SPIONs and QDs; preparation of PNCAS; loading the drug CUR to PNCAS; measurement of drug loading and encapsulation efficiency; TEM, DLS, NTA, zeta potential, SQUID, TGA, and fluorescence spectroscopy analysis.

### 4.4 Conjugation of PNCAS With Tat Peptide and Physicochemical Characterizations of PNCAS‐Tat

PNCAS (with or without drug) was prepared with BSA (sulfhydryl blocked), was centrifuged for 45 min at 13 500 rpm (0.4 mg PNCAS per tube), and were re‐suspended in PBS (1 mL, pH 7.4). Then, a specific amount of SMCC (in DMF) was added to react with ‐NH_2_ in BSA on the surface of PNCAS for 30 min at 25°C, with the molar ratio of PNCAS: SMCC being 1: 1000, 2000, 4000 et al. for conjugation with different amounts of Tat peptide. The mixed solution was then centrifuged at 13 500 rpm for 45 min and re‐suspended with PBS (1 mL, pH 6.8). A solution of Tat peptide (with a cysteine at the N‐terminus, in PBS with pH 6.8) was added and shaken overnight at 4°C (the molar ratio of SMCC:Tat was 1:1). The mixed solution was centrifuged at 13 500 rpm for 45 min. The Tat‐conjugated PNCAS was collected and re‐suspended with PBS (pH 7.4) for further study.

### 4.5 Biogenesis of Exosomes

#### 4.5.1 Biogenesis by PNCAS‐Tat Stimulation

PNCAS‐Tat were dissolved in DMFM/F12 medium (containing FBS) and incubated with BMSCs for 8 h (the endocytosis phase). The cells were subsequently washed with PBS three times. Fresh DMFM/F12 medium (no FBS) was added and BMSCs were incubated for 16 h (the exocytosis phase). The cell supernatant (containing exosomes) was collected for isolation or analysis.

#### 4.5.2 Conventional Biogenesis (Stimulation by Serum Starvation)

BMSCs were cultured in DMFM/F12 medium (containing FBS) for 8 h and washed with PBS three times. Subsequently, the DMFM/F12 medium (containing FBS) was replaced by fresh DMFM/F12 medium (no FBS) for 16 h. The cell supernatant (containing exosomes) was collected for isolation or analysis.

### 4.6 Isolation of Exosomes

#### 4.6.1 MIMS‐Based Isolation of Exosomes (Containing Magnetic Nanoparticles)

The cell supernatant was centrifuged at 2000 g for 10 min to remove dead cells and cell debris, and the supernatant was transferred to a container in a MIMS setup (details of the magnet and instrument can be seen in the main text). The isolation of magnetic nanoparticles‐containing exosomes was conducted by slow rotation (e.g., 5 rpm) of the MIMS magnet in the container. The magnet (with exosomes attached to the surface) was transferred to another container with PBS. A brief (10 s) bath sonication was performed to release the exosomes to the buffer solution.

#### 4.6.2 Isolation of Exosomes by Ultracentrifugation

The cell supernatant was centrifuged at 2000 g for 10 min to remove dead cells and cell debris. Ultracentrifugation was performed at 100 000 g for 70 min, washed with PBS, and another round of ultracentrifugation was performed at 100 000 g for 70 min (Beckman Optima L‐XP).

### 4.7 Characterizations of Exosomes

The size and number concentration of exosomes were analyzed by NTA, in which exosomes were either labeled with DiL dye for fluorescent detection or were unlabeled for scattering detection. The surface biomarkers (CD 63, TSG 101, and CD 81) of exosomes were analyzed by Western blotting. The morphology of exosomes was visualized by TEM (JEM‐200CX, JEOL).

The number of PNCAS in each exosome was analyzed by two different methods, namely TEM and elemental analysis of iron, which yielded similar results. TEM provided direct visualization of the number of PNCAS in an exosome. The elemental analysis of iron was conducted by an inductively coupled plasma optical emission spectrometer (ICP‐OES). First, ICP‐OES coupled with NTA were employed to determine the average amount of iron in each PNCAS (in a dispersion of PNCAS, NTA gave the number of PNCAS, and ICP‐OES gave the total amount of iron). Then, in a dispersion of PNCAS‐encapsulated exosomes, ICP‐OES was used to determine the total amount of iron, and combined with the above result of the average amount of iron in each PNCAS, to calculate the number of PNCAS in the dispersion. Finally, NTA was used to determine the number of exosomes in the dispersion of PNCAS‐encapsulated exosomes, and combined with the result of the PNCAS number in the dispersion, to calculate the average number of PNCAS in an exosome.

### 4.8 Endotoxin Measurement

A recombinant factor C (rFC) assay was conducted using the Rhinogen Recombinant Factor C Endpoint Fluorescent Assay Kit (RHINO BIO#RAF‐02). To eliminate exogenous endotoxin contamination from the instrument used for experiments, the equipment was subjected to dry heat sterilization at 250°C for a minimum of 2 h. The standard endotoxin (National Institutes for Food and Drug Control, Cat. #150601) was prepared by dilution in nonpyrogenic water. Both sample solutions and diluted standard endotoxin were dispensed into a nonpyrogenic white 96‐well plate in duplicate or greater replicates. For quality control, each sample group included a corresponding spike recovery group to assess the efficiency of standard recovery. Following a pre‐incubation of the plate in a 37°C cell incubator for 30 min, each well was treated with the fluorogenic substrate and rFC enzyme solution. Fluorescence was measured at 0 and 60 min using a microplate reader (SpectraMax I3X, Molecular Devices). A standard curve was generated using serially diluted standard endotoxin samples ranging from 0.005 to 5 EU/mL. The endotoxin levels in the test samples were calculated based on the standard curve.

### 4.9 Live Cell Imaging

Live cell imaging studies were performed using a live cell spinning‐disk confocal imaging system (SpinSR10, Olympus, Japan) consisting of a cell culture chamber, an epi‐fluorescent microscope, a spinning‐disk confocal system, and two EMCCD cameras for simultaneous dual‐channel recording. Image processing and analysis were conducted using the Olympus analysis and Image J software.

### 4.10 TEM of Cells

After incubating PNCAS‐Tat with cells for a specific time duration (e.g., 8, 24 h), the cells were washed with PBS three times and trypsinized for 2 min. The cells were harvested and fixed by glutaraldehyde (2.5%, 24 h) and osmium tetroxide (1%, 2 h). The cells were dehydrated in graded acetone. The cells were embedded in Epon and cut into thin slices (100 nm thickness). The cells were then stained with uranyl acetate/lead citrate for TEM analysis.

### 4.11 RT‐qPCR

Total RNA was extracted from BMSCs using the RNA easy kit (Qiagen, UK) according to the manufacturer's instructions. RNA samples were treated with DNase I (Invitrogen, USA) and quantified using a NanoDrop spectrophotometer (NanoDrop One/OneC, ThermoFisher, USA). Genes (Rab5a‐NM_022692.2; Rab7a‐NM_023950.4; β‐Actin‐NM_031144.3) were determined relative to β‐Actin according to the TaqMan gene expression assay protocol (Applied Biosystems/Life Technologies, UK). TaqMan primers and probes were purchased from Servicebio (Wuhan, China). The primer sequences used were as follows:

**Table:** 

Rab5a (Norway rat)	Forward: 5'‐GAGCAACAAGACCCAACGGGC‐3'
	Reverse: 5'‐ATGGCGGCTTGTGCTCCTCG‐3
Rab7a (Norway rat)	Forward: 5'‐TGTTGGAAAGACATCGCTCAT‐3'
	Reverse: 5'‐TCACCTCCTTGGTCAGAAAGT‐3
β‐Actin (Norway rat)	Forward: 5'‐GTTACGCGCTCCCTCATGCC‐3'
	Reverse: 5'‐ATGGCGGCTTGTGCTCCTCG‐3

All TaqMan PCR reactions were performed with three biological replicates, and each replicate was tested in duplicate.

### 4.12 Western Blotting

Total protein was extracted from BMSCs using RIPA lysate (with 0.1% of PMSF protease inhibitor, Servicebio, China) according to the manufacturer's instructions. Protein concentration was measured with a Bradford protein‐assay (BCA) kit (Adamas Life, China). Equal aliquots of protein (30 µg) were boiled for 5 min in 6 × loading buffer and separated on 10% SDS‐PAGE gels. Samples were transferred to PVDF membranes (0.45 µm), and probed with anti‐CD 63 (1:500, GB12620‐100, Servicebio, China), anti‐CD 81 (1:500, GB111073‐100, Servicebio, China), anti‐actin (1:800, ab179467, Abcam, UK), anti‐TSG 101 (1:500, GB15618‐100, Servicebio, China), anti‐Rab 5 (1:500, #3547, Cell Signaling Technology, USA), anti‐Rab 7 (1:500, #9367, Cell Signaling Technology, USA), anti‐Beclin‐1 (1:500, GB112053‐100, Servicebio, China), anti‐LC 3A/B (1:300, ab62721, Abcam, UK) antibodies. Blocking was carried out in BSA buffer without antibody for 1 h at room temperature. The membranes were incubated with secondary antibody (horseradish peroxidase‐conjugated goat anti‐rabbit or goat anti‐mouse 1:5000) for 30 min, followed by three washes. Protein bands were visualized with chemiluminescence reagents (ECL, Servicebio, China) and analyzed by the Image J software.

### 4.13 Proteomic Analysis

Exosomes or BMSCs were lysed by sonication and the total protein concentration was measured by a BCA protein assay kit. Protein samples were initially dissolved in 8 M urea (Greagent, China), followed by treating with 12 mM Tris (2‐carboxyethyl) phosphine hydrochloride (Adamas Life, China) at 37°C for 1 h and 16 mM iodoacetamide (Adamas Life, China) at room temperature for 1 h in the dark. The solution was diluted to a urea concentration of less than 1.5 M. Then trypsin (10 µg, Sequencing grade, Adamas Life, China) was added to the solution and protein digestion was performed overnight at 37°C. The resulting peptides were further purified using C18 (S03230G‐A‐100G, SiliCycle, Canada) solid‐phase extraction and quantified with a BCA Protein assay Kit. The protein lysates of BMSCs and exosomes were analyzed by high‐performance liquid chromatograph‐mass spectrometry (HPLC‐MS) (Thermo Fisher Scientific, USA). Raw data were processed and analyzed by Spectronaut 14 (Biognosys AG, Switzerland) with default settings. The MS raw data were searched against the Uniprot FASTA database within the default parameters. Adjust *p*‐values (p.adj cutoffs on precursor and protein levels were set at 0.05 or 0.01). Proteins significantly up‐regulated were included for GO and KEGG analysis.

### 4.14 MRI in Live Animals

MRI image acquisition on mice was performed using a 9.4 T MRI scanner (Bruker 9.4 T BioSpec 94/30 MRI, Germany). Before an MRI image acquisition, the mouse was anesthetized with 2% isoflurane. T2‐weighted images were acquired using a turboRARE‐T2 sequence with the following parameters: repetition time/echo time = 2500/36.0 ms, field of view = 2.00/1.80 cm, matrix size = 56 × 256, and slice thickness = 0.5 mm (20 slices per animal). T2* maps were calculated using a pixel‐wise linear fitting algorithm on the series of images.

### 4.15 BBB‐Crossing Studies

#### 4.15.1 In Vitro Study

Astrocytes extracted from SD rat were seeded on the outer bottom of a Transwell insert placed upside down in a six‐well plate at a density of 1 × 10^5^ cells/cm^2^. The plate was then cultured in an incubator for 8 h for cell adherence. HUVECs were then seeded on the inner bottom of the Transwell insert at a density of 1 × 10^5^ cells/cm^2^. The cell culture medium was exchanged daily for a week. After the cells proliferated to a sufficient density, the trans‐endothelial electrical resistance (TEER) values were analyzed by an electrical resistance system. A model with TEER values above 220 Ω·cm^2^ was considered a suitable BBB model and was used for subsequent experiments. A sample (e.g., PNCAS‐Tat, PNCAS‐Tat‐encapsulated exosomes) was added to the top compartment of a Transwell BBB model, and, when needed, a magnet was placed under the bottom compartment of the Transwell BBB model. The amount of iron in the sample was determined by ICP‐OES after drying and acid hydrolysis. At different time points (24, 48 h), the cell culture medium in the bottom compartment was collected, and ICP‐OES was employed to obtain the iron amount. The percentage of particles that have crossed the BBB was determined by dividing these two iron amounts.

#### 4.15.2 In Vivo Study

C57BL/J6 mice were anesthetized with 4% chloral hydrate, samples (e.g., PNCAS‐Tat, PNCAS‐Tat‐encapsulated exosomes) were injected into mice from the caudal vein. Here, the PNCAS contained both QDs and SPIONs with a molar ratio of 2:1. Magnets were placed on the skull of the mice for 3 h. Mice were euthanized by CO_2_ at 48 h after injection, and the mice brains were obtained and fixed in PFA for 24 h. The fixed brain tissues were embedded and coronal serial sectioned at 5 mm. The brain tissues were then dehydrated and dewaxed. The amount of BBB crossing was analyzed by fluorescence of QDs or Prussian blue staining of SPIONs in the brain tissue samples.

### 4.16 Animal Procedures for In Vivo Studies of Therapeutic Effects

Each group included 5 mice kept in an SPF environment. The weight of mice was maintained at 25–35 g before the experiment.

PD animal model was induced with a 10‐day consecutive intraperitoneal injections of MPTP solution (22 mg/mL, Beyotime Biotechnology, China) in C57BL/J6 mice (10‐month‐old, male). PD mice were given treatment by tail vein injection of a formulation consisting of CUR‐loaded PNCAS‐Tat, or MSCs‐derived exosomes (without CUR or PNCAS‐Tat), or PNCAS‐Tat‐encapsulated MSCs‐derived exosomes (without CUR), or CUR‐loaded PNCAS‐Tat‐encapsulated MSCs‐derived exosomes, with each injection containing 1 × 10^9^ particles performed every 3 days for a total of 30 days. PBS was injected to PD mice in the negative control sample. For those formulations with CUR, the CUR loading in PNCAS was 53%. For all treatments with tail vein injection, a magnet was placed on top of the mice skulls for 3 h for magnetism‐enhanced delivery. Behavior analysis of mice was conducted as follows. A rotating rod was used to analyze the motor coordination of mice. The mice were placed on a horizontal rod with a rotation rate 30 rounds per minute. The number of drops within 1 min was recorded for five times each mouse. An open field test was used to analyze the spontaneous activity of mice. A 500 mm × 500 mm × 300 mm field was used with a camera located directly above to cover the entire field. The mice were placed in the central grid and photographed with the camera for 5 min. A computer tracking analysis system was used to analyze the activities of mice. Mice were euthanized and brain tissues were collected for histological and immunochemical examination.

Pulmonary fibrosis model was induced with a single intratracheal injection of a bleomycin sulfate solution (1.6 mg/kg, Sigma–Aldrich, USA) in C57BL/J6 mice (6‐week‐old, male). At day 7, by which point lung fibrosis had started, the mice were given inhalation treatments using a nebulizer (PARI 047F45‐LCS, USA) every 2 days for 14 days with a formulation of CUR‐loaded PNCAS‐Tat‐encapsulated MSCs‐derived exosomes, or CUR‐loaded PNCAS‐Tat, or MSCs‐derived exosomes (without CUR or PNCAS‐Tat), or PNCAS‐Tat‐encapsulated MSCs‐derived exosomes (without CUR), or PBS (equal volume), with each dose containing 1 × 10^9^ particles (except the PBS formulation). For those formulations with CUR, the CUR loading in PNCAS was 53%. Animals were euthanized, and lung tissues were collected for histological and immunochemical examination.

The skin injury mice model was established by using a sterile circular biopsy perforator (with a diameter of 8 mm) to perform full‐layer skin excision on both sides of the shaved and disinfected mouse back, forming a circular full‐layer skin wound (0.6 cm). The mice were given treatment every 3 days by subcutaneous injection of a formulation (each injection containing 1 × 10^9^ particles) of CUR‐loaded PNCAS‐Tat‐encapsulated MSCs‐derived exosomes, or CUR‐loaded PNCAS‐Tat, or MSCs‐derived exosomes (without CUR or PNCAS‐Tat), or PNCAS‐Tat‐encapsulated MSCs‐derived exosomes (without CUR). For those formulations with CUR, the CUR loading in PNCAS was 53%. Animals were euthanized, and skin tissues were collected for histological and immunochemical examination.

### 4.17 Histology

Immunostaining was performed on tissue slides (e.g., mouse brains, lungs, and skins) fixed in 4% paraformaldehyde (PFA) for 15 min, followed by permeabilization with 0.3% Triton X‐100 for 10 min and blocking with 5% BSA solution for 2 h prior to antibody staining. Next, the tissue slides were incubated with primary antibodies against TH (1:500, ab137869, Abcam), or CD 31 (1:50, ab28364, Abcam), or collagen I (1:500, ab270993, Sigma–Aldrich), or α‐SMA (1:200, ab5694, Abcam). Tissue slides were then incubated with second antibodies (1:1000, ab288151, Abcam) for 3 h at room temperature. Finally, tissue slides were scanned using a slide scanning system (SLIDEVIEW VS200, Olympus). Histochemistry score (H‐score) was used for the positive cell rate analysis using ImageJ software.

Hematoxylin‐Eosin (H&E) staining and Masson's trichrome staining were performed on paraffin‐embedded tissue sections (thickness: 5 µm). H&E‐stained lung sections were used for pulmonary fibrosis Ashcroft scoring. Ashcroft score was performed by averaging the scores from one blinded and one non‐blinded scorer. H&E‐stained skin sections were used for scar length scoring using ImageJ software. Masson‐stained lung and skin were used for collagen content analysis (collagen fiber area/tissue area of the test area) using ImageJ software.

### 4.18 Establishing In Vitro Disease Models of Cells

#### 4.18.1 PD Cell Model

SH‐SY5Y cells were treated with a solution of MPP+ iodide (1.5 µM, Adamas Life, China) in DMEM containing 10% FBS for 24 h. Different treatments were added to the cells for 24 h. The cells were then collected and analyzed.

#### 4.18.2 Ischemia Cell Model of Cardiomyocytes

H9C2 cells (cardiomyocytes) were cultured in 96‐well plates or 6‐well plates with a cell density of ∼30% before ischemia treatment. After reaching the optimal density, cells were put into the hypoxia cell culture chamber with 1% O_2_, 5% CO_2,_ and 94% N_2_, at 37°C and in 100% humidified atmosphere for 24 h to mimic ischemia. This was followed by another 24 h incubation in the standard cell culture chamber with 21% O_2_, 5% CO_2,_ and 74% N_2_, as the reperfusion mimicry to cause ROS accumulation. Different treatments were added to the cells for 24 h. The cells were then collected and analyzed.

#### 4.18.3 PCOS Cell Model

KGN cells were treated with 10 mM of dehydroepiandrosterone (DHEA) for 24 h. Different treatments were added to the cells for 24 h. The cells were then collected and analyzed.

### 4.19 Lipid Peroxidation (MDA) Assay

To assess oxidative stress in cells, the MDA assay was performed. After treatments, SH‐SY5Y cells were harvested and lysed on ice for 20 min, and then centrifuged at 12 000 rpm for 5 min to obtain the supernatant. The protein content was analyzed using the BCA kit (Adamas Life, China), and MDA content was analyzed using the Lipid Peroxidation MDA Assay Kit (Adamas Life, China) according to the manufacturer's protocol. In brief, 0.2 mL of the supernatant, 3 mL of MDA assay buffer, and ddH2O (1 mL) were mixed and boiled for 50 min, and then quickly cooled in cold water. The cooled mixture was centrifuged at 3 000 rpm for 15 min at 4°C. After centrifugation, light absorption was measured using a microplate reader (measurement wavelength 532 nm, Infinite 200PRO, Switzerland). MDA level was presented as the ratio of MDA concentration to protein concentration.

### 4.20 Glutathione (GSH) Assay

SH‐SY5Y cells were harvested and lysed at ice for 10 min, and then centrifuged at 13 500 rpm for 10 min to obtain the supernatant. The protein content was analyzed using the BCA kit (Adamas Life, China), and GSH was analyzed using the GSH detection kit (KeyGEN, China) according to the manufacturer's protocol. In brief, 20 µL of supernatant and 180 µL of GSH assay buffer were mixed for 2 min at room temperature. Light absorption was measured using a microplate reader (measurement wavelength 412 nm, Infinite 200PRO, Switzerland). GSH level was presented as the ratio of GSH concentration to protein concentration.

### 4.21 Cell Apoptosis Assay

Cells (SH‐SY5Y, or H9C2, or KGN) were collected using 0.25% trypsin without ethylenediaminetetraacetic acid (EDTA). Cells were washed thrice with PBS, and apoptosis was measured using the annexin V‐FITC/PI apoptosis detection kit (Adamas Life, China) according to the manufacturer's protocol. In brief, the washed cells were re‐suspended in 100 µL of 1 × Annexin V binding buffer and mixed with Annexin V‐FITC (5 µL) and PI (10 µL) in the dark at room temperature for 15 min. The cells were then added with 1 × Annexin V binding buffer (400 µL). The stained cells were analyzed by flow cytometry (FACSCelesta, BD Biosciences, USA) using FITC (494/518 nm) and PI (535/617 nm) channels, respectively. A total of 10,000 cell counts were collected per sample.

### 4.22 Reactive Oxygen Species (ROS) Assay

Cells (SH‐SY5Y, or H9C2, or KGN) were collected using 0.25% trypsin with EDTA. All cells were washed twice with DMEM/F12 medium (no FBS), and the ROS level was measured using the ROS detection fluorescent probe DHR123 (KeyGEN, China) according to the manufacturer's protocol. In brief, the washed cells were re‐suspended in DMEM/F12 medium (1 mL, no FBS) and mixed with DHR123 (10 µL) in the dark at room temperature for 10 min. The cells were then centrifuged at 1000 rpm for 5 min. The stained cells were re‐suspended and analyzed by flow cytometry (FACSCelesta, BD Biosciences, USA) using the FITC (494/518 nm) channel. A total of 10 000 cell counts were collected per sample.

### 4.23 In Vivo Toxicity and Immunogenicity Assays

A high dose of engineered exosomes was administered into healthy Kunming mice by intravenous injection (1 × 10^10^ particles/mouse). After 24 h, whole blood was collected. Liver function indexes (ALT, AST, LDH, ALP, total bilirubin), kidney function indexes (uric acid, urea, and creatinine), and immunoreaction indexes (white blood cell, neutrophil, lymphocyte, and monocyte) in the serum were analyzed by a detection kit according to the manufacturer's instructions (Roche).

In addition, in PD mice, 10 injections of engineered exosomes (each injection 1 × 10^9^ particles) were administered to each mouse by intravenous injection in a course of 30 days (one injection every 3 days). At the end of the 30‐day period, the mice were sacrificed, and major organs (heart, liver, spleen, lung, kidney) were sliced for an H&E staining study of tissue morphology.

### 4.24 Statistical Analysis

Data are presented as mean ± standard deviation (SD). Statistical analysis was performed using a two‐tailed, unpaired *t*‐test or one‐way ANOVA followed by Tukey's multiple comparisons test, as appropriate. A *p*‐value less than 0.05 was designated statistically significant. Statistical analysis was performed with GraphPad Prism 10 software.

## Author Contributions

G.R. conceptualized and supervised this work, and wrote the manuscript. X.W. led the biology studies, including manufacturing, mechanistic studies, and applications, and wrote the initial draft of the manuscript. Z.X. led the nanomaterial development, and conducted the initial reduction‐to‐practice of the MIMS design. Z.H. conducted a large portion of the biology studies and performed substantial optimizations of MIMS. Y.C. performed significant optimizations of MIMS. K.X. performed a substantial portion of the biological application studies. H.Y. and X.W. made substantial contributions to the biological application studies. J.M. made a significant contribution to the nanomaterial development. S.S. made a significant contribution to the biological application studies. B.C., C.L., and M.X. were involved in the omics studies. Y.C. was involved in neural system studies.

## Funding

XJTLU Research Development Funding RDF‐21‐02‐007, XJTLU PGRS FOSA2212024, XJTLU PGRS FOSLG240406, XJTLU PGRS FOSST240901, and Basic Research Program of Jiangsu (No. BK20251811).

## Conflicts of Interest

G.R., X.W., Z. X., Z. H., Y. C. have filed patent applications based on this work. G. R. is a co‐founder and equity holder of Core Quantum Technologies Inc., the manufacturer of a commercial reagent (MagDot) used in this work.

## Supporting information




**Supporting File 1**: advs74394‐sup‐0001‐SuppMat.pdf.

**Supporting File 2**: advs74394‐sup‐0002‐Video1.pptx.

## Data Availability

The data that support the findings of this study are available from the corresponding author upon reasonable request.
