# Supplementary material for: A Nanoparticle‐Integrated Complete Manufacturing Pipeline of Chemically Engineered Exosomes
Source: Adv Sci (Weinh). 2026 Mar 24;13(32):e16075. doi: 10.1002/advs.202516075 (PMC13252649; doi:10.1002/advs.202516075)
Supplement: Supplementary file 1 — Supporting File 1: advs74394‐sup‐0001‐SuppMat.pdf. [file ADVS-13-e16075-s002.pdf]

# Supporting Information

## A Nanoparticle-Integrated Complete Manufacturing Pipeline of Chemically Engineered Exosomes

*Xiaowei Wen, Zixing Xu, Zerun Hao, Yanming Chen, Kai Xie, Haofan Yin, Xueying Wang, Jie Min, Sihan Sun, Baiding Chen, Chengxiu Ling, Mingming Xu, Yizhao Chen, Gang Ruan**

Corresponding author: G. Ruan, Email: Gang.Ruan@xjtlu.edu.cn
These authors contributed equally to this work: X. Wen, Z. Xu, and Z. Hao.

This Supporting Information file contains the following:

Figure S1-S24

Table S1

Video S1

Note S1-S2

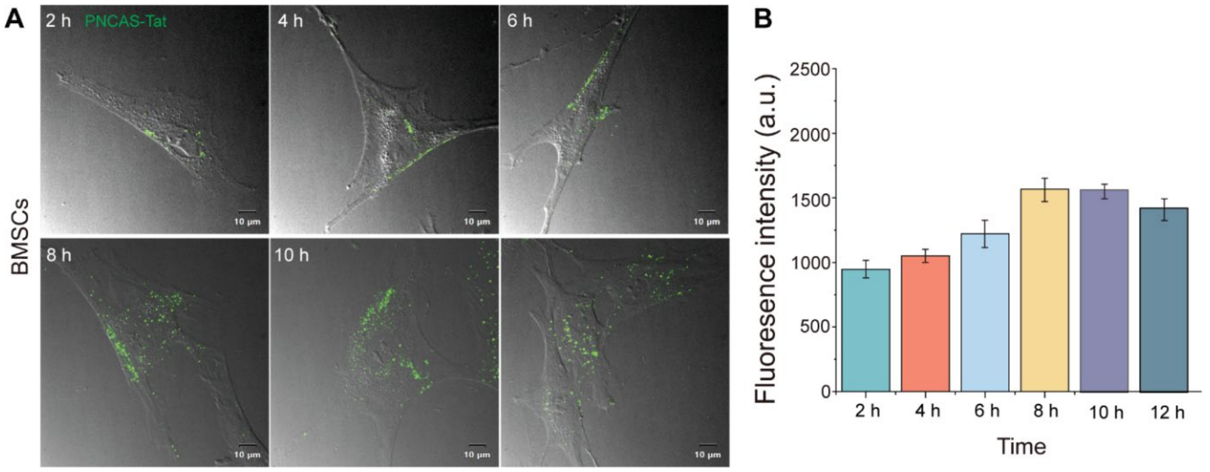


**Figure S1. Kinetics of net cellular uptake of PNCAS-Tat in BMSCs.** Quantum dots (QDs) were used in PNCAS-Tat to emit bright and stable fluorescence. Confocal fluorescence microscopy was used to visualize PNCAS-Tat in BMSCs at different time points of incubation. **(A)** shows representative microscopy images. Scale bar: 10 μm. **(B)** shows the quantification results based on imaging. n = 50 cells. Data are presented as mean ± SD. Concentration of PNCAS-Tat used: 0.12 mg/mL. Tat number per PNCAS used: 2000. Passage number used: 4.

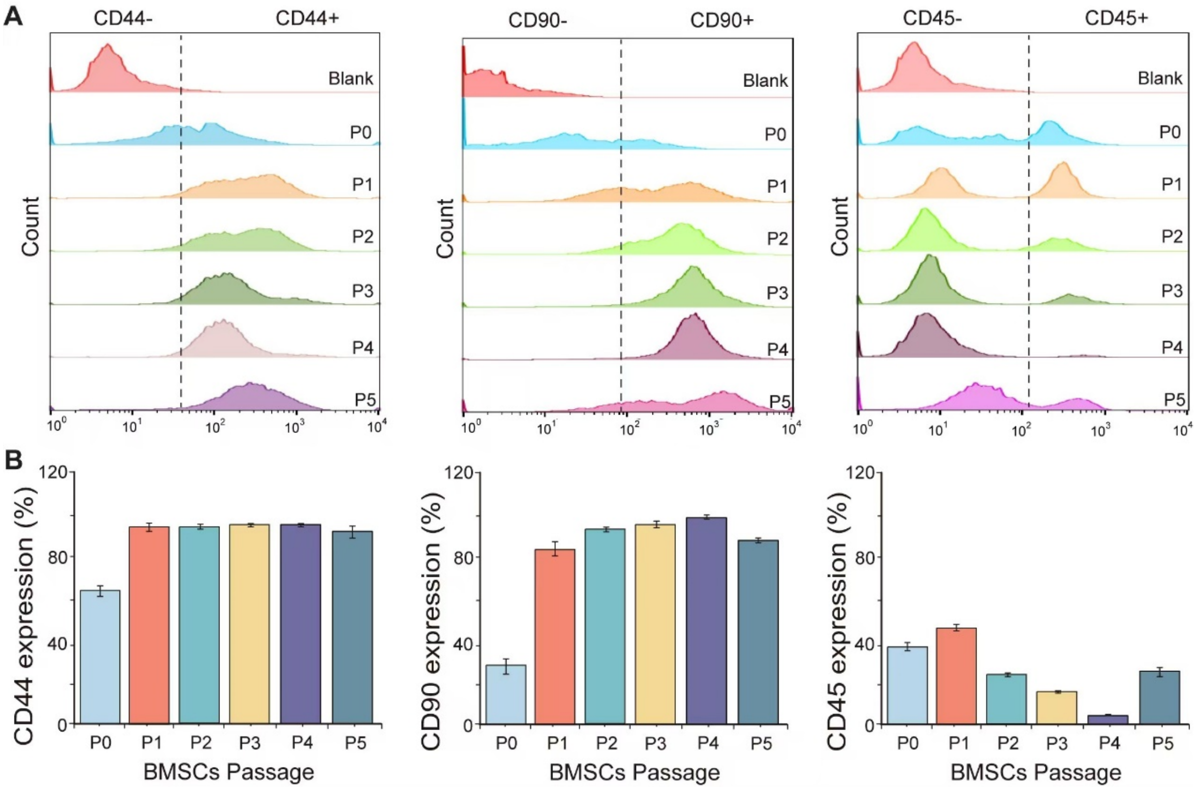


**Figure S2. MSC identification by molecular markers and change of molecular marker expression with different MSC passage number.** A MSC should have a high expression of CD44 (CD44+), a high expression of CD90 (CD90+), and a low expression of CD 45 (CD45-). Here flow cytometry was used to analyze the expression of CD44, CD90, and CD45 of BMSCs at different passages (P0-P5). P0: passage 0; P1: passage 1; P2: passage 2; P3: passage 3; P4: passage 4; P5: passage 5. **(A)** Flow cytometry histograms. **(B)** Quantification results based on flow cytometry histograms. Data are from three independent experiments and are presented as mean ± SD.

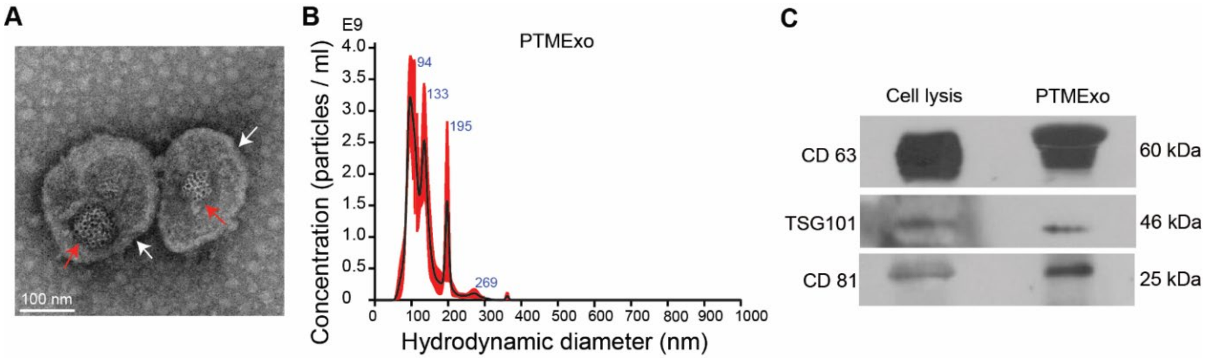


**Figure S3. Characterizations of PNCAS-Tat-encapsulated MSC-exosomes isolated by MIMS (PTMExo for short in the figure). (A)** Transmission electron microscopy (TEM) image. White arrows point to exosomes. Red arrows point to PNCAS-Tat (encapsulated in exosomes). Number of exosomes used to quantify the size of exosomes and the number of PNCAS in each exosome: 50. Scale bar: 100 nm. **(B)** Nanoparticle Tracking Analysis (NTA) result. **(C)** Western blotting bands of molecular markers of exosomes (CD 63, TSG 101 and CD 81).

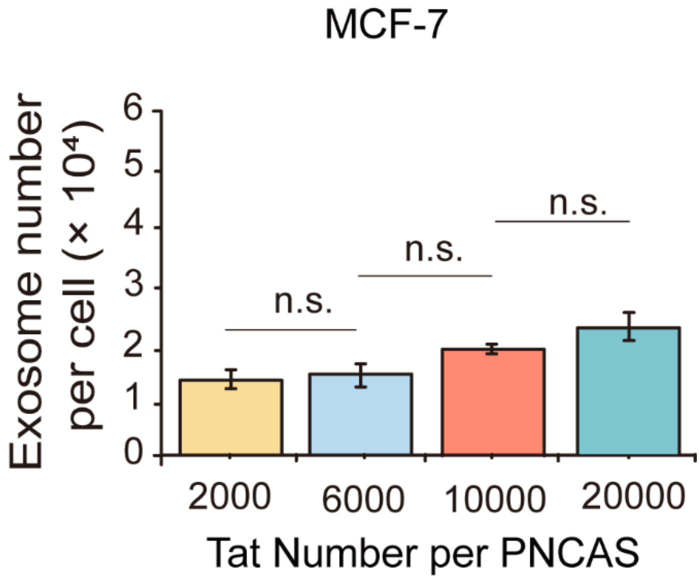


**Figure S4. In MCF-7 cells, effect of Tat number per PNCAS on the number of exosomes generated per cell.** PNCAS-Tat concentration used: 0.12 mg/mL. Exocytosis time used: 16 h. Data are from three independent experiments and are presented as mean ± SD. Data are analyzed with one-way ANOVA followed by Tukey's multiple comparisons test. n.s., not significant.

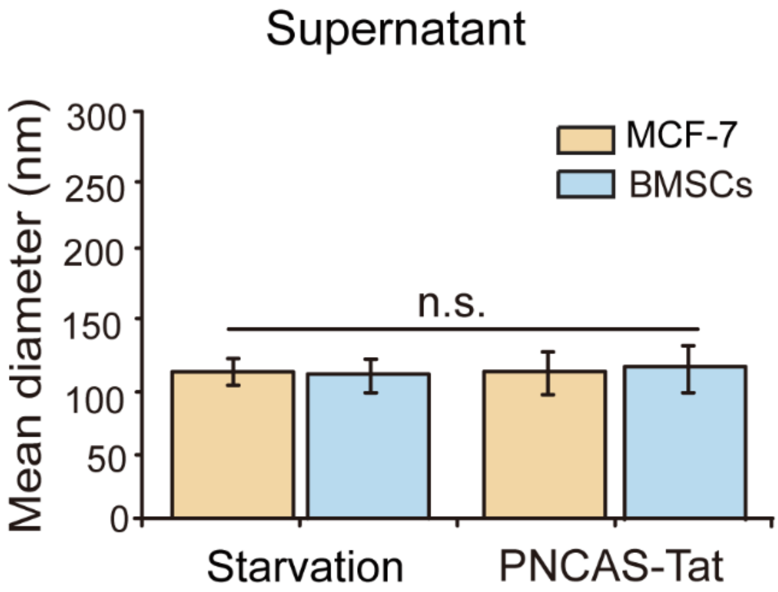


**Figure S5. Size comparison, as measured by NTA, of the exosomes generated by different stimulation methods (starvation *vs.* PNCAS-Tat) and in different cell types (BMSCs *vs.* MCF-7 cells).** Data are from three independent experiments and are presented as mean ± SD. Data are analyzed with a two-tailed, unpaired t-test. n.s., not significant.

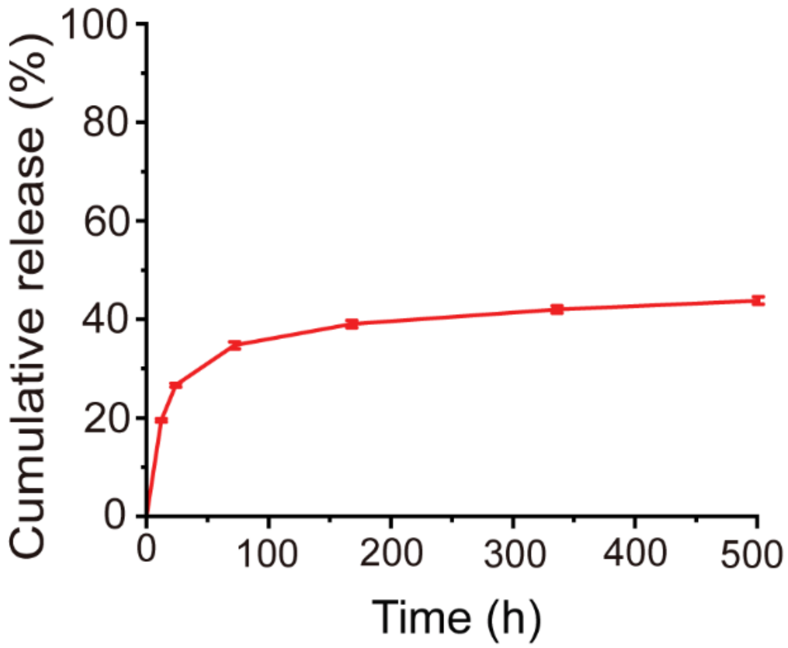


**Figure S6.** ***In vitro*** **release kinetics of curcumin from PNCAS-Tat in PBS at 37 °C.**

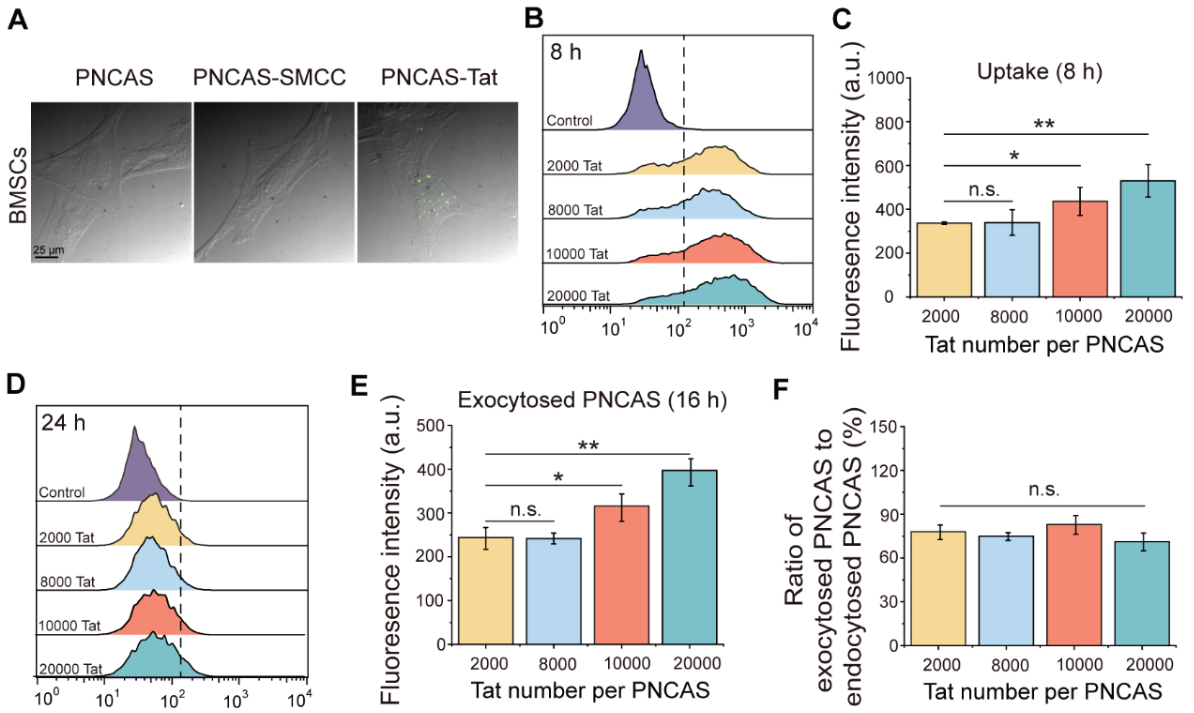


**Figure S7. Effects of Tat number per PNCAS on the amounts of endocytosed PNCAS-Tat and exocytosed PNCAS-Tat in BMSCs. (A)** Confocal microscopy images of BMSCs after incubation for 8 h with PNCAS, PNCAS-SMCC, and PNCAS-Tat, respectively. The PNCAS used here contained QDs emitting green fluorescence color. Tat number per PNCAS used here for the PNCAS-Tat sample: 2000. Scale bar: 25 μm. **(B,C)** Flow cytometry analysis of endocytosis of PNCAS-Tat into BMSCs. The control sample in (B) used PNCAS without Tat for cellular uptake. **(D,E)** Flow cytometry analysis of exocytosis of PNCAS-Tat. (D) measures the fluorescence intensity remaining in cells at the end of the exocytosis phase. (E) calculates the amount of exocytosed PNCAS-Tat by deducting the result of (D) from that of (B). **(F)** Ratio of exocytosed PNCAS-Tat to endocytosed PNCAS-Tat. The values are calculated from the results of (E,C). In (B-F), data are from three independent experiments and are presented as mean ± SD; data are analyzed with one-way ANOVA followed by Tukey's multiple comparisons test. n.s., not significant; * $P < 0.05$; ** $P < 0.01$.

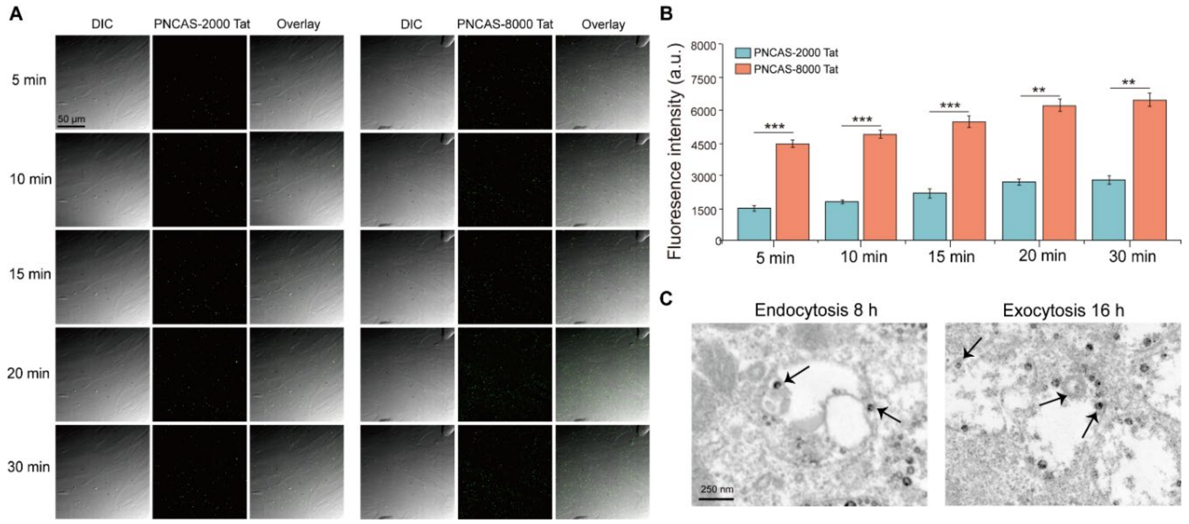


**Figure S8. PNCAS-Tat has strong affinity with membranes in cells (BMSCs). (A)** Confocal fluorescence microscopy of the earliest stage (5 min-30 min) of cellular uptake of PNCAS-Tat. Left: 2000 Tat per PNCAS; Right: 8000 Tat per PNCAS. Scale bar: 50 μm. **(B)** Quantification of fluorescence intensity based on the fluorescence images in (A). Number of cells studied for each set of experimental conditions: 100. Data are presented as mean ± SD, and are analyzed with a two-tailed, unpaired t-test. ** $P < 0.01$; *** $P < 0.001$. **(C)** TEM of BMSCs to show that inside the cells PNCAS-Tat often adhered to the inner membranes of intracellular vesicles. Black arrows point to PNCAS-Tat. Left: image captured at 8 h of the endocytosis phase; Right: image captured at 16 h of the exocytosis phase. Scale bar: 250 nm.

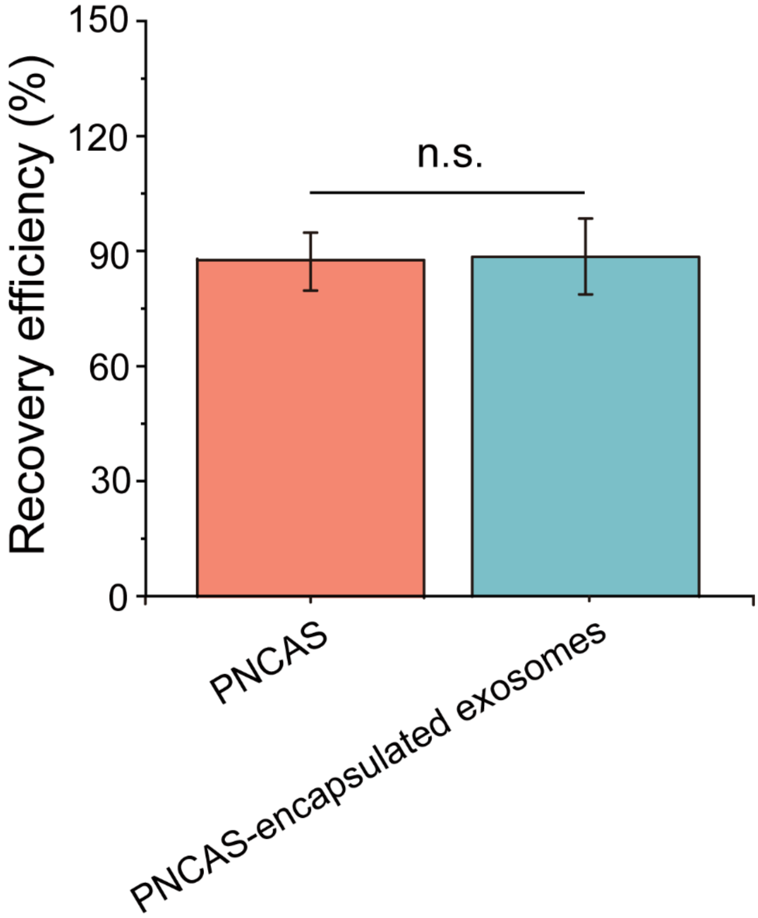


**Figure S9. Recovery efficiency of PNCAS and PNCAS-encapsulated exosomes using MIMS.** Data are from three independent experiments and are presented as mean ± SD. Data are analyzed with a two-tailed, unpaired t-test. n.s.; not significant.

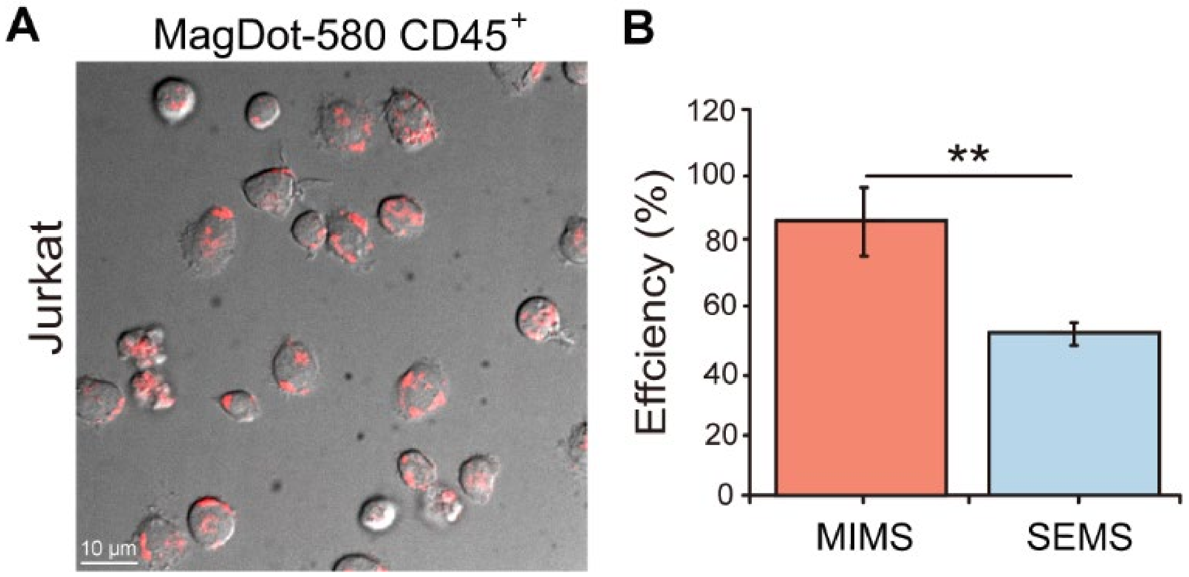


**Figure S10. Preliminary experiments of using MIMS for cell isolation.** A commercial nanoparticle probe was used. **(A)** Image of cells labelled by a commercial nanoparticle probe, namely MagDot-antibody conjugate, which incorporates QDs (red fluorescence), SPIONs, and antibody. The particle size of a MagDot is ~50 nm in TEM diameter (similar to PNCAS). **(B)** Comparison of isolation efficiency of cells after 1 h by MagDot-antibody conjugate using different magnet designs: conventional magnet design SEMS *vs.* MIMS. Data are from three independent experiments and are presented as mean ± SD. ** $P < 0.01$. Data are analyzed with a two-tailed, unpaired t-test. It is worth noting that, because in this cell isolation experiment each cell was attached to a large number of magnetic nanoparticles (MagDots), it was easier to isolate cells in this way than to isolate exosomes with just 1 to 2 PNCAS inside the exosomes, given the much larger magnetic force generated on each cell.

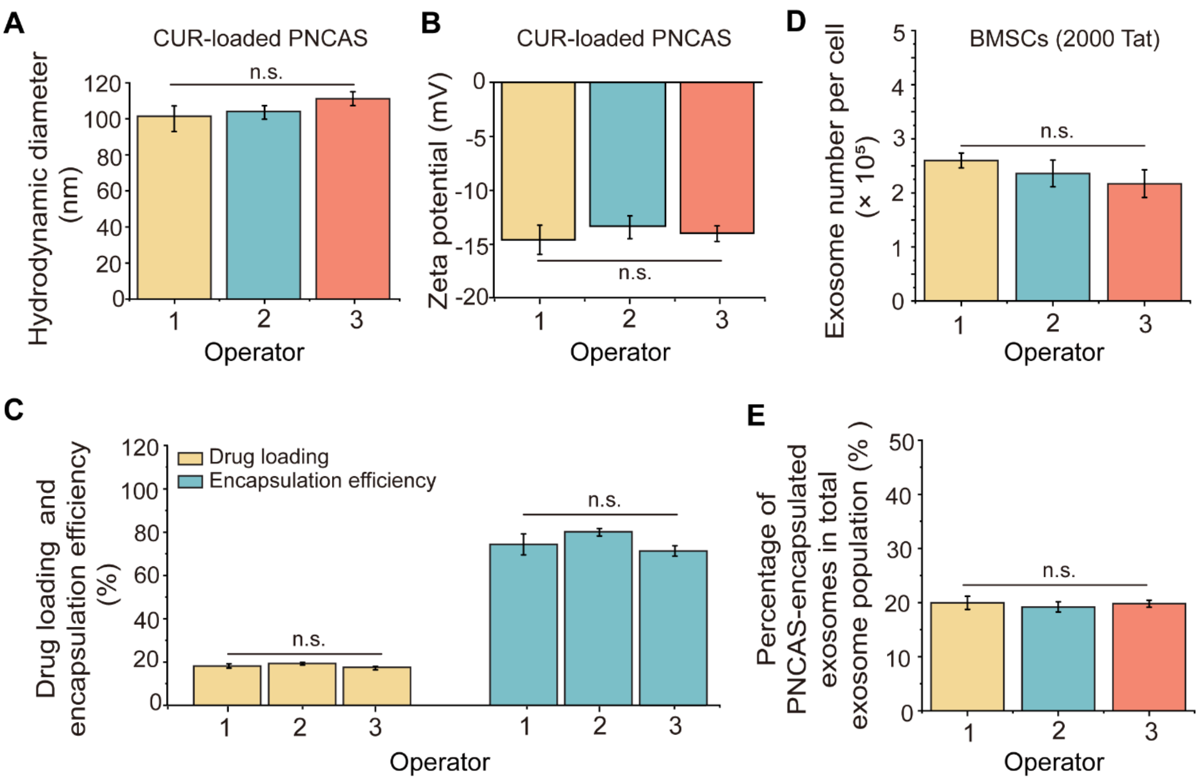


**Figure S11. Quality control of the manufacturing process: reproducibility.** 3 different operators were assigned to conduct the manufacturing, with each operator processing 3 samples in parallel to complete the entire manufacturing flow. The results show reproducibility both within the same operator and between different operators. **(A)** Reproducibility of hydrodynamic diameter of CUR-loaded PNCAS measured by DLS. **(B)** Reproducibility of zeta potential (surface charge) of CUR-loaded PNCAS. **(C)** Reproducibility of drug loading and encapsulation efficiency of CUR-loaded PNCAS. Drug concentration used in the drug loading: 2 mg/mL. **(D)** Reproducibility of number of exosomes generated per cell (BMSC). Tat number per PNCAS used: 2,000. **(E)** Reproducibility of percentage of PNCAS-encapsulated exosomes (isolated by MIMS) within the total exosome population from BMSCs. Tat number per PNCAS used: 2,000. Data are from three independent experiments and are presented as mean ± SD. Data are analyzed with one-way ANOVA followed by Tukey's multiple comparisons test. n.s., not significant.

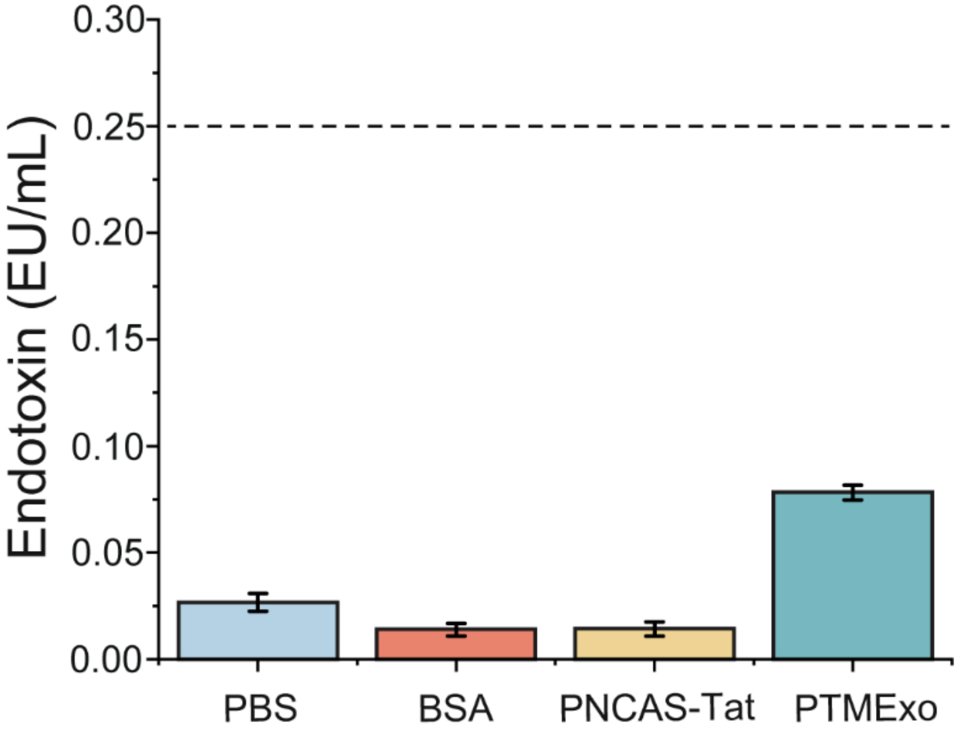


**Figure S12. Quality control of the manufacturing process: contamination by endotoxin.** The dashed line at 0.25 Endotoxin Units (EU)/mL refers to the upper limit set by the US FDA guideline on sterile water for injection and irrigation. The endotoxin levels of several key points of quality control along the manufacturing process are shown, including PBS, BSA (2 mg/mL), PNCAS-Tat ($10^9$ particles), PNCAS-Tat-encapsulated MSCs-derived exosomes (PTMExo in the figure, that is, the final product) ($10^9$ particles). Data are from three independent experiments and are presented as mean ± SD. The several mean values shown in the figure are 0.027 EU/mL for PBS, 0.014 EU/mL for BSA, 0.010 EU/mL for PNCAS-Tat, and 0.083 EU/mL for PTMExo, respectively.

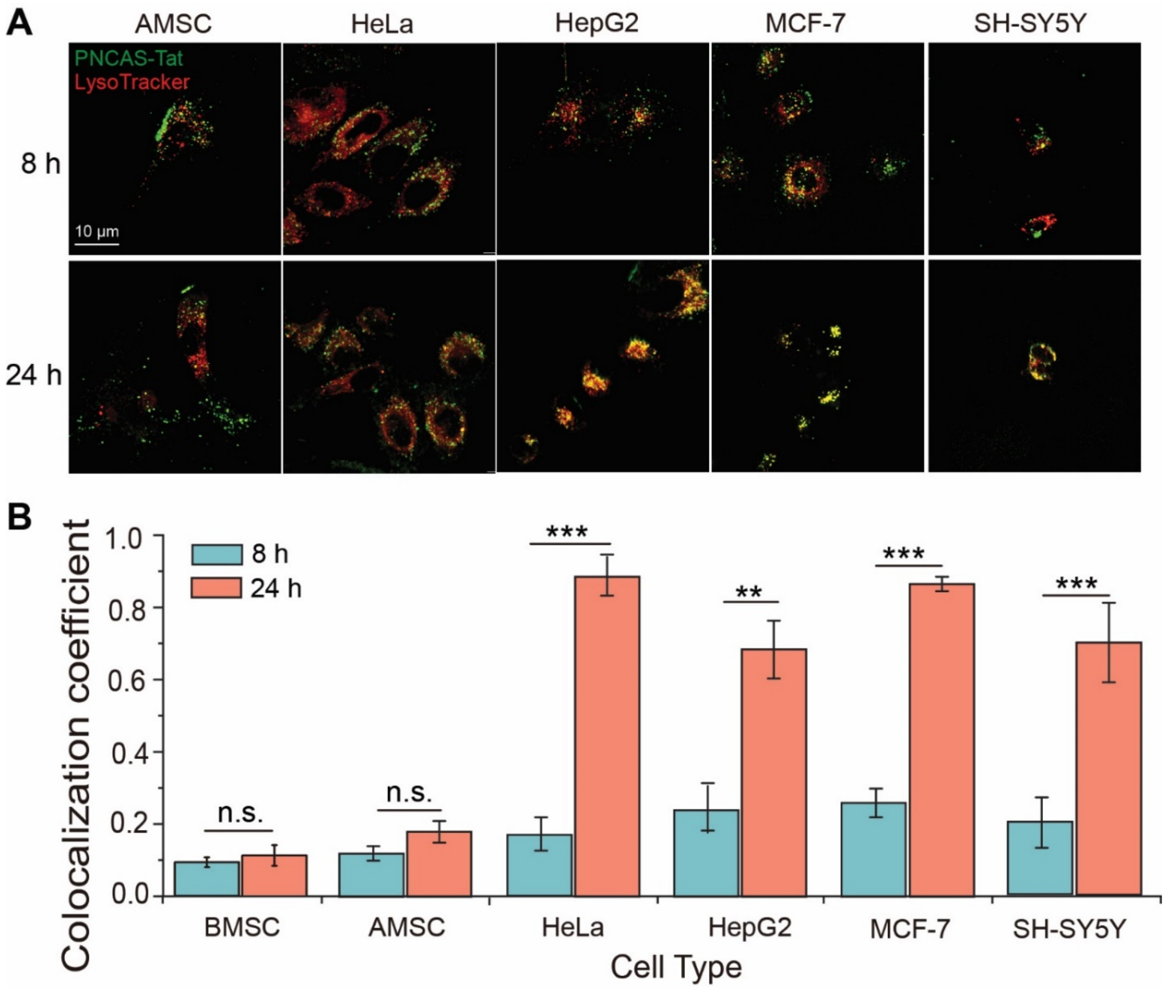


**Figure S13. Colocalization analysis of PNCAS-Tat with lysosomes in various cell types.** Lysosomes were stained by LysoTracker (red). PNCAS-Tat contained QDs (green). The cell types examined include 2 MSCs and 5 cancer cell lines. **(A)** Confocal fluorescence images for the colocalization analysis. **(B)** Quantification results of Manders colocalization coefficient based on the images. The images for BMSCs are in Fig. 6D. n = 100 cells. Data are presented as mean ± SD, and are analyzed with a two-tailed, unpaired t-test. n.s.: no significance; ** $P < 0.01$; *** $P < 0.001$.

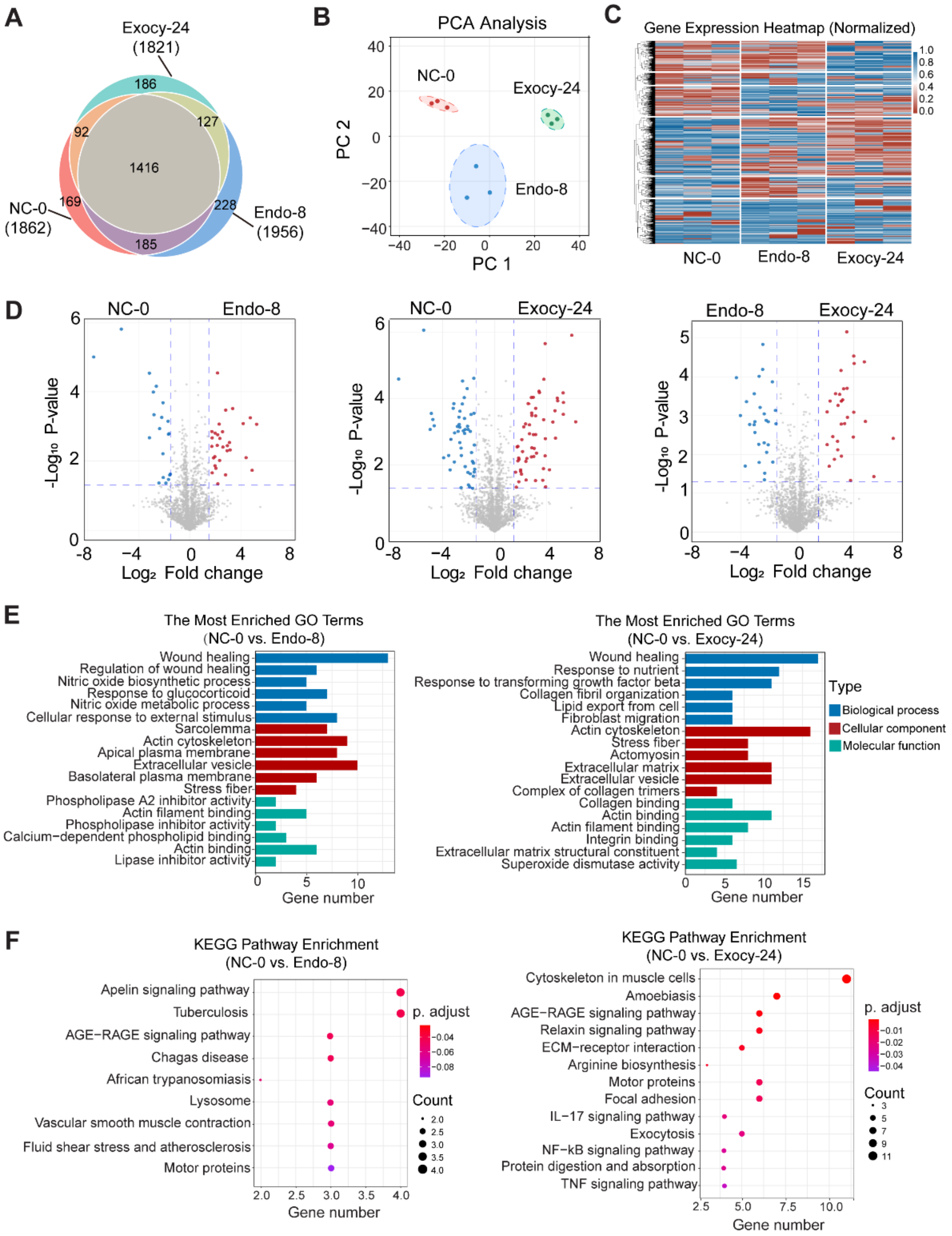


**Figure S14. Proteomic analysis of BMSCs with or without PNCAS-Tat incubation.** In this figure NC-0 refers to cells withou PNCAS-Tat at time point 0; Endo-8 refers to cells with PNCAS-Tat at 8 h (endocytosis phase); Exocy-24 refers to cells with PNCAS-Tat at 24 h (exocytosis phase). **(A)** Venn diagram showing shared and different proteins. **(B)** Principal component analysis (PCA). **(C)** Heatmap of the proteins identified from the proteomic profiling of BMSCs. **(D)** Volcano plots showing differently expressed proteins (DEPs) (fold change >1.5 and P value <0.05). **(E)** GO enrichment analysis of significantly up-regulated proteins. **(F)** KEGG pathway analysis of significantly up-regulated proteins. Data are from three independent experiments.

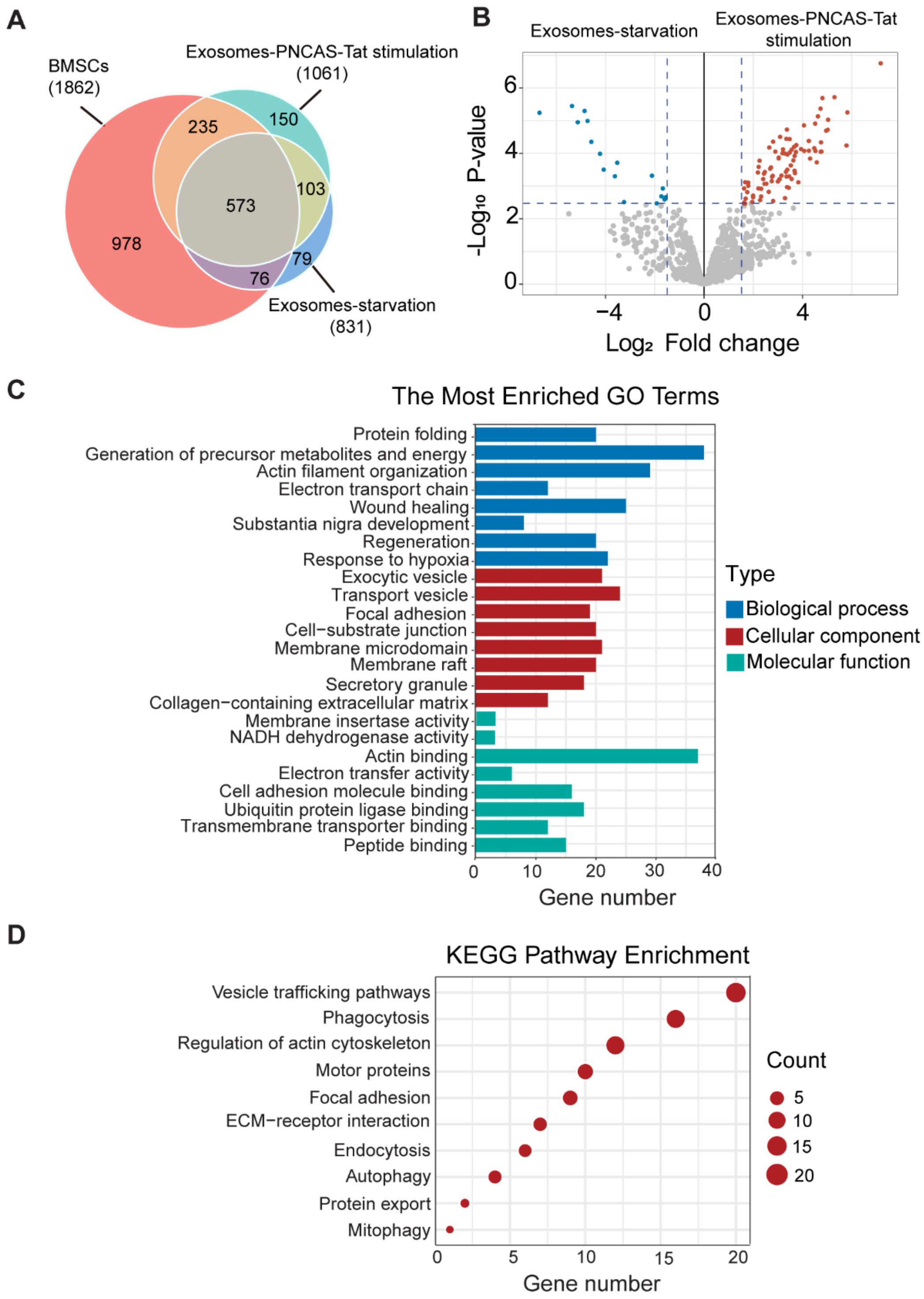


**Figure S15. Proteomic analysis of engineered MSC-exosomes (stimulated by PNCAS-Tat, isolated by MIMS) in comparison with conventional exosomes (stimulated by starvation). (A)** Venn diagram showing shared and different proteins. **(B)** Volcano plots showing differently expressed proteins (DEPs) (fold change >1.5 and P value <0.05). **(C)** GO enrichment analysis of significantly up-regulated proteins. **(D)** KEGG pathway analysis of significantly up-regulated proteins. Data are from three independent experiments.

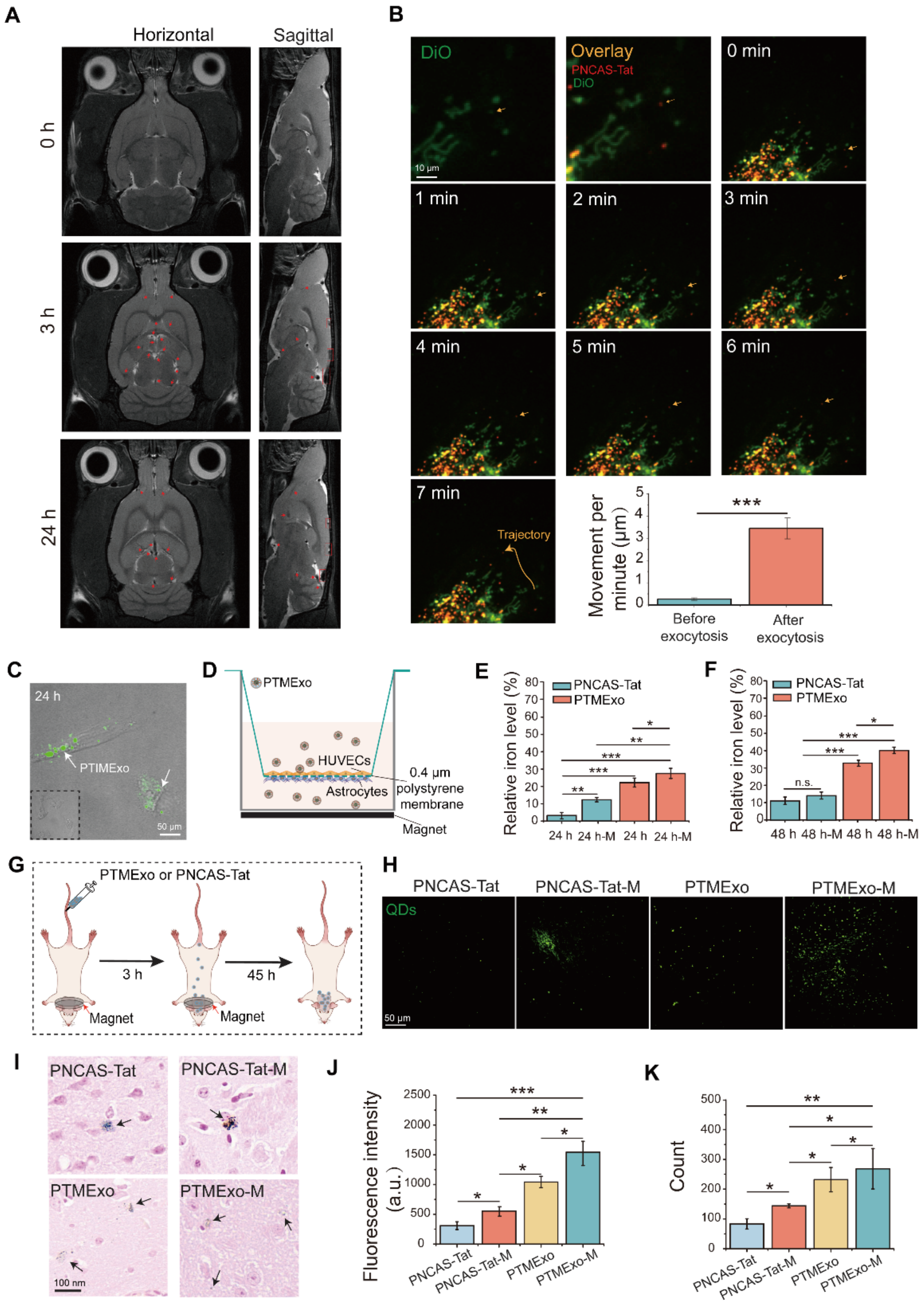


**Figure S16. Applications in neurological disease: imaging and delivery. (A-C) Demonstration of the imaging capacity of the engineered exosomes (PNCAS-Tat-encapsulated MSCs-derived exosomes) offered by the encapsulated nanoparticles (SPIONs or QDs). (A)** T2*-weighted MRI imaging of mouse brains at different time points after intravenous injection of engineered exosomes. Coronary and sagittal views of the brains are shown. The red arrows point to engineered exosomes (with encapsulated SPIONs). **(B)** Spinning disk confocal fluorescence imaging and tracking of a PNCAS-encapsulated vesicle (with green QDs) that was likely exiting the source cell (BMSC). The vesicle was stained by a red vesicle dye DiO. The images are from a video (Supplementary Video 1, video capture frame rate 60 frames/h) showing the dynamic process of a PNCAS-encapsulated vesicle's movement. Colocalization between green QDs and red vesicle dye DiO in throughout the frames of the video suggest that it is a PNCAS-encapsulated vesicle (indicated by an arrow). In the video, this PNCAS-encapsulated vesicle was initially stationary (likely bound to the cell membrane), and then suddenly showed rapid movement (likely diffusing after being released to the extracellular environment). At the downright corner of (B), a quantitative comparison is shown on the rate of movement between these two stages (described in the figure as 'before exocytosis' and 'after exocytosis', respectively). Data are presented as mean ± SD and are analyzed with a two-tailed, unpaired t-test. *** $P < 0.001$. **(C)** Confocal fluorescence imaging of the uptake of

PNCAS-Tat-encapsulated MSCs-derived exosomes ('PTMExo' in the image) by SH-SY5Y cells, a neuroblastoma cell line, after 24 h of incubation. The green fluorescence is from the QDs in the engineered exosomes. The image at the bottom-left corner shows a control image, in which no 'PTMExo' was used to incubate with the cells. Scale bar: 50 μm. **(D-K) Studies of the ability of engineered exosomes to cross the blood-brain barrier (BBB) enhanced by magnetism.** The different groups examined are as follows: PNCAS-Tat without magnet, PNCAS-Tat with magnet**,** PTMExo (PNCAS-Tat-encapsulated MSCs-derived exosomes) without magnet, PTMExo with magnet. Equal particle number concentration was used in the different groups. **(D)** Schematic of the Transwell experimental design to examine the BBB crossing ability *in vitro*. A magnet was placed below the well to exert magnetic attractive force on PNCAS (with SPIONs). Two time durations of delivery, namely 24 h and 48 h, were examined. The amount of delivery across BBB was quantified by measuring the iron content using ICP-OES. **(E)** Amounts of delivery across BBB in the Transwell model at 24 h measured by ICP-OES. '24 h-M' refers to groups with magnet treatment. **(F)** Amounts of delivery across BBB in the Transwell model at 48 h measured by ICP-OES. '48 h-M' refers to groups with magnet treatment. In (E,F), data are from three independent experiments and are presented as mean ± SD. Data are analyzed with one-way ANOVA followed by Tukey's multiple comparisons test. n.s., not significant; * $P < 0.05$; ** $P < 0.01$; *** $P < 0.001$. **(G)** Schematic of the experimental design to examine the BBB crossing ability *in vivo*. A magnet was placed on top of the mouse skull for 3 h to exert magnetic attractive force on PNCAS (containing SPIONs) towards the brain. Light microscopy was performed on the brain slices at the 48 h time point, using the fluorescence of QDs or Prussian blue staining. **(H)** Images of confocal fluorescence microscopy of brain slices at the 48 h time point. QDs emit green fluorescence. Scale bar: 100 μm. **(I)** Images of Prussian blue staining of brain slices at the 48 h time point. Arrows point to PNCAS (containing SPIONs) stained by Prussian blue. Scale bar: 100 μm. **(J)** Quantification of (H). n = 100 cells. **(K)** Quantification of (I). n = 100 cells. In (H-K), '-M' refers to magnet treatment. In (J, K), data are presented as mean ± SD; * $P < 0.05$; ** $P < 0.01$; *** $P < 0.001$. Data are analyzed with one-way ANOVA followed by Tukey's multiple comparisons test.

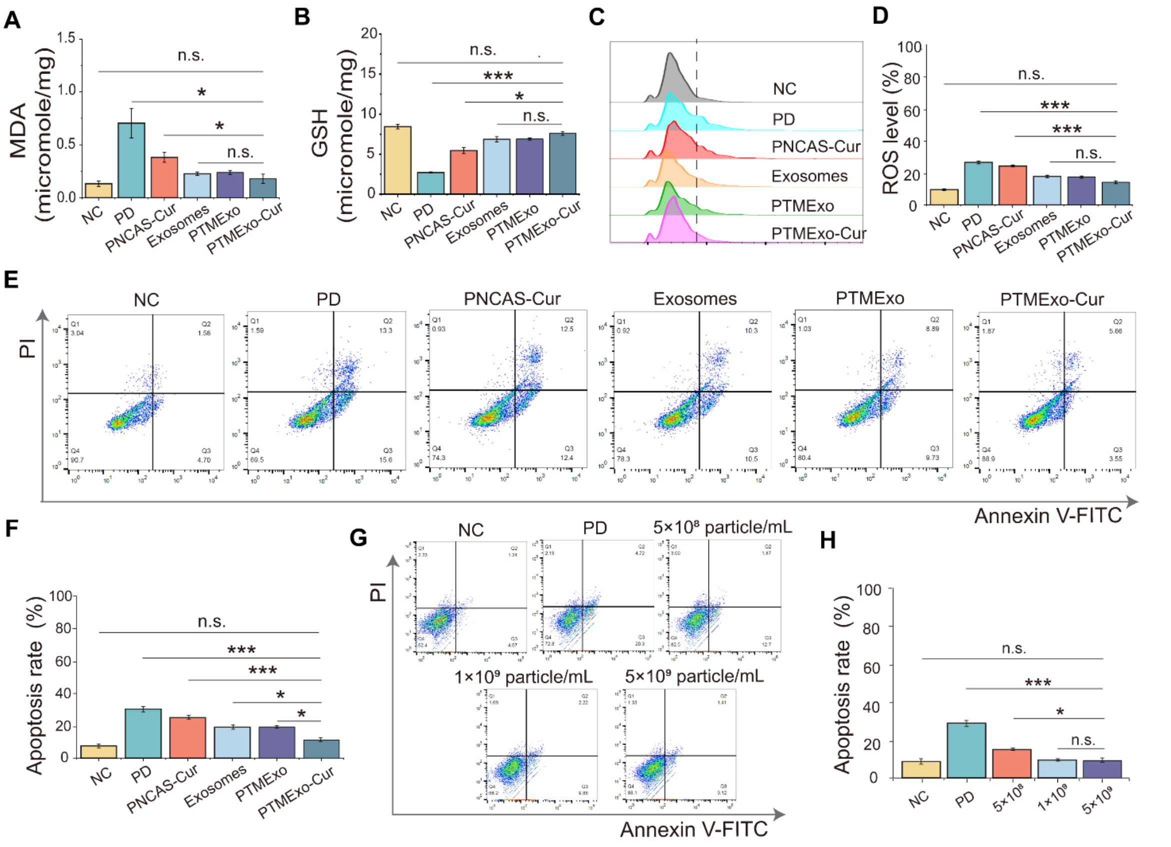


**Figure S17.** ***In vitro*** **cell model study of the therapeutic effects of curcumin (CUR)-loaded, PNCAS-Tat-encapsulated, MSCs-derived exosomes on Parkinson's Disease (PD).** SH-SY5Y cells were treated with MPP (1-methyl-4-phenylpyridinium) to establish the PD cell model, followed by different therapeutic treatments for 24 h (concentration $1\times10^9$ particles/mL, drug loading 53% in PNCAS for those formulations with CUR loading). NC: Normal cell control (no therapeutic treatment); PD: PD cells with no therapeutic treatment; PNCAS-Cur: PD cells treated with CUR-loaded PNCAS-Tat; Exosomes: PD cells treated with conventional MSCs-derived exosomes (no PNCAS, no CUR); PTMExo: PD cells treated with PNCAS-Tat-encapsulated MSCs-derived exosomes (no CUR); PTMExo-Cur: PD cells treated with CUR-loaded, PNCAS-Tat-encapsulated MSCs-derived exosomes. **(A)** Quantification of the level of oxidative stress biomarker MDA in cells after incubation with different treatments. **(B)** Quantification of the level of antioxidative stress biomarker GSH in cells after incubation with different treatments. **(C)** Flow cytometry analysis of reactive oxygen species (ROS) in cells after incubation with different treatments. **(D)** Quantification of the level of ROS in cells after incubation with different treatments. **(E)** Flow cytometry analysis of apoptosis in cells after incubation with different treatments. **(F)** Quantification of the level of apoptosis in cells after incubation with different treatments. **(G)** Flow cytometry analysis of the effect of different concentrations of the formulation PTMExo-Cur on cell apoptosis. **(H)** Quantification of the effect of different concentrations of the formulation PTMExo-Cur on cell apoptosis. Data are from three independent experiments and are presented as mean ± SD. Data are analyzed with one-way ANOVA followed by Tukey's multiple comparisons test. n.s., not significant; * $P < 0.05$; ** $P < 0.01$; *** $P < 0.001$.

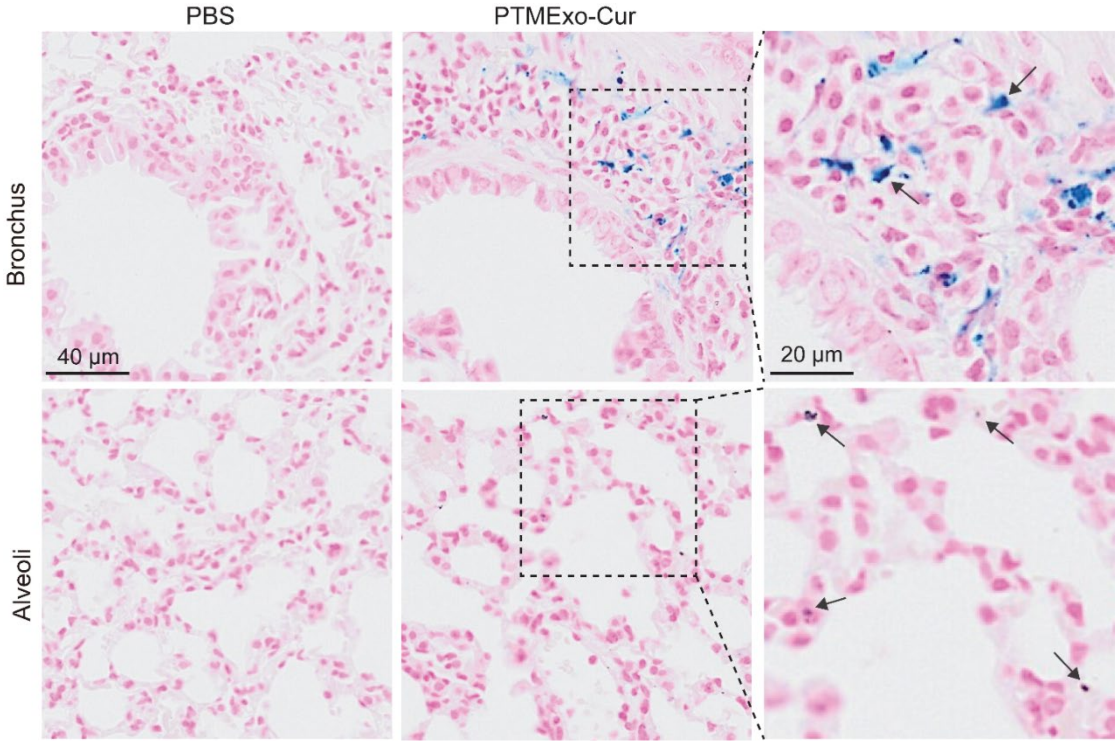


**Figure S18. Distribution of engineered exosomes ("PTMExo-Cur" in the figure, *i.e.,* CUR-loaded, PNCAS-Tat-encapsulated, MSCs-derived exosomes) in lung tissues of IPF mice after inhalation, as analyzed by Prussian blue staining.** Black arrows point to Prussian blue staining of engineered exosomes (containing SPIONs). It can be seen that the engineered exosomes can reach both bronchus and alveoli.

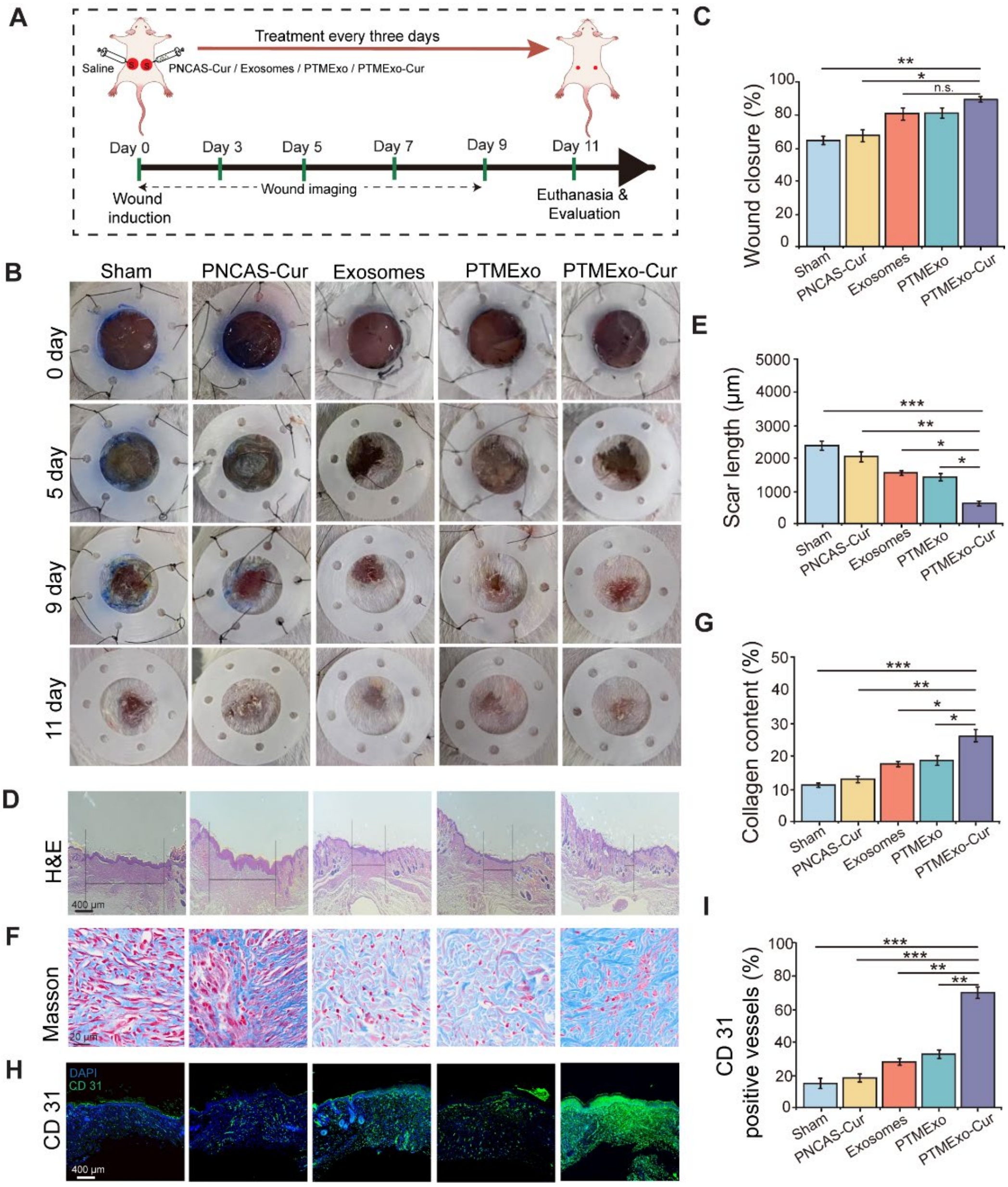


**Figure S19. Application of engineered exosomes in wound healing in mice model.** The initial wounds were 0.6 cm on the back skin of mice. The different mice groups include Sham (wounded mice with saline treatment), PNCAS-Cur (wounded mice treated with CUR-loaded PNCAS-Tat), Exosomes (wounded mice treated with MSCs-derived exosomes without PNCAS-Tat or CUR), PTMExo (wounded mice treated with PNCAS-Tat encapsulated MSCs-derived exosomes without CUR), and PTMExo-Cur (wounded mice treated with CUR-loaded PNCAS-Tat-encapsulated MSCs-derived exosomes). **(A)** Schematic of experimental procedure. **(B)** Images of wounds after different treatments. **(C)** Quantification of the wound closure based on the images in (B). **(D)** H&E staining of mice skin after different treatments for 11 days. **(E)** Quantification of scar length based on the H&E staining results. **(F)** Masson staining of mice skin after different treatments for 11 days. **(G)** Quantification of collagen content based on the Masson staining results. **(H)** Immunohistochemistry of CD31 (also called PECAM-1), a biomarker of new blood vessel formation (angiogenesis), after different treatments for 11 days. **(I)** Quantification of the CD31 immunohistochemistry results. Data are presented as mean ± SD (n = 5 mice) and analyzed with one-way ANOVA followed by Tukey's multiple comparisons test. n.s., not significant; * $P < 0.05$; ** $P < 0.01$; *** $P < 0.001$.

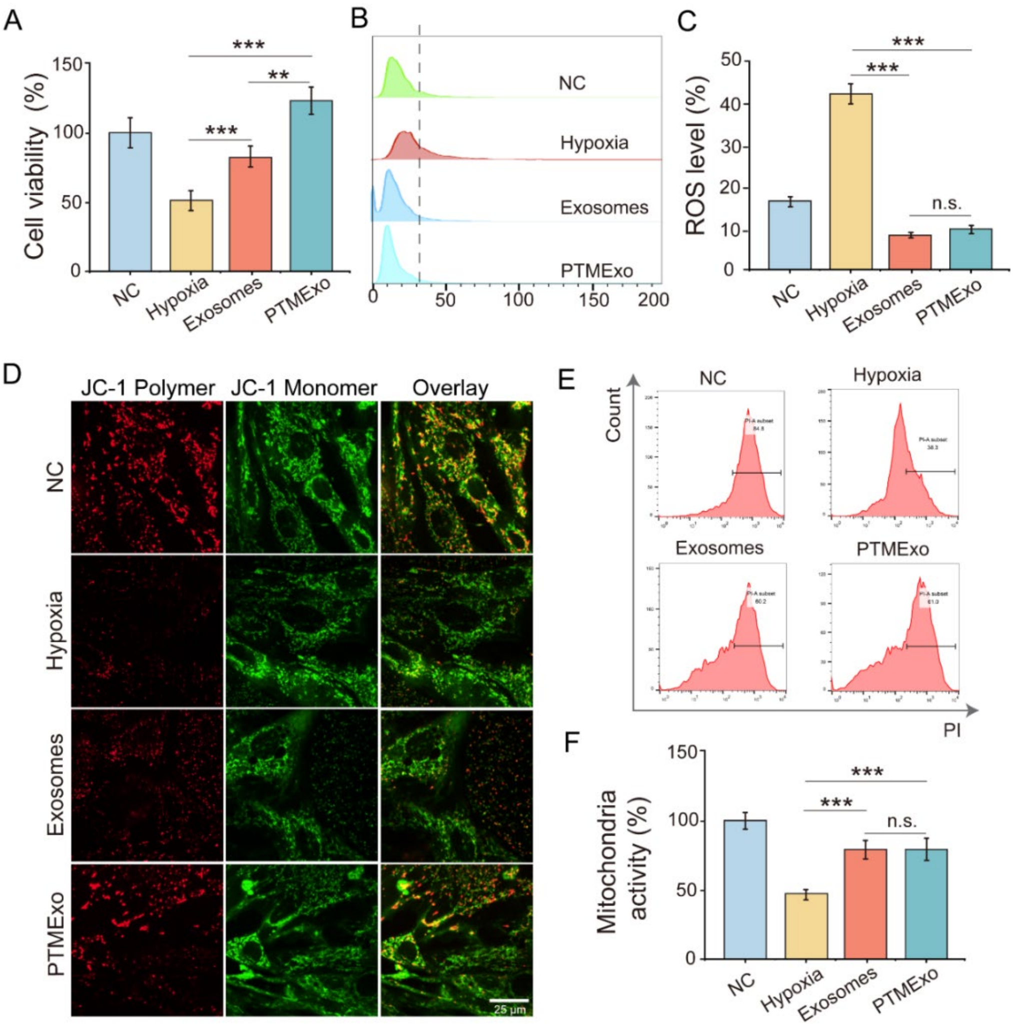


**Figure S20. Application of engineered exosomes in treating ischemic heart disease in a cell model.** The cell model was established by treating H9C2 cells (cardiomyocytes) under hypoxia stress. The diseased cells were treated with either MSCs-derived exosomes or PNCAS-Tat-encapsulated MSCs-derived exosomes ($1\times10^9$ particles/mL, 24 h). The different groups in this figure are as follows. NC: normal cell control; Hypoxia: diseased cells with no treatment; Exosomes: diseased cells treated with MSCs-derived exosomes; PTMExo: diseased cells treated with PNCAS-Tat encapsulated MSCs-derived exosomes. **(A)** Cell viability analysis after incubation with different formulations for 24 h. **(B)** ROS levels of cells after different treatments as analyzed by flow cytometry. **(C)** Quantification of the flow cytometry results of ROS levels. **(D)** Confocal fluorescence images of JC-1 dye in H9C2 cells after incubation with different formulations for 24 h. JC-1 dye allows measurement of mitochondria activity, using the ratio of red to green fluorescence intensity. Red fluorescence is from JC-1 polymer; green fluorescence is from JC-1 monomer. A higher red to green ratio indicates healthier mitochondria. **(E)** Flow cytometry analysis of JC-1 dye in H9C2 cells after incubation with different formulations for 24 h. **(F)** Quantification of the results of (E). Data are from three independent experiments and are presented as mean ± SD. Data are analyzed with one-way ANOVA followed by Tukey's multiple comparisons test. n.s., not significant; ** $P < 0.01$; *** $P < 0.001$.

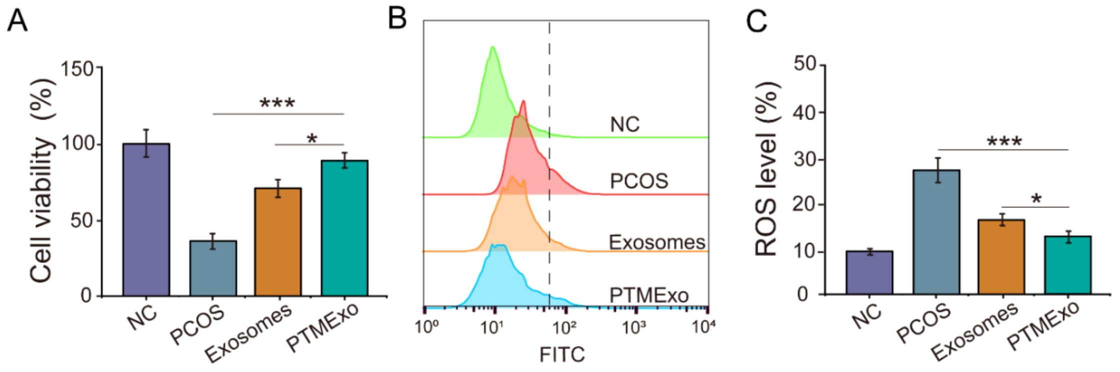


**Figure S21. Application of engineered exosomes in treating reproductive disease in a cell model of polycystic ovary syndrome (PCOS).** The hyper-androgen PCOS cell model was established by treating KGN cells with DHEA (dehydroepiandrosterone). The diseased cells were treated with either MSCs-derived exosomes or PNCAS-Tat-encapsulated MSCs-derived exosomes ($1\times10^9$ particles/mL, 24 h). The different groups in this figure are as follows. NC: normal cell control; PCOS: diseased cells with no treatment; Exosomes: diseased cells treated with MSCs-derived exosomes; PTMExo: PNCAS-Tat-encapsulated MSCs-derived exosomes. **(A)** Cell viability analysis after incubation with different formulations for 24 h. **(B)** ROS levels of cells after different treatments as analyzed by flow cytometry. **(C)** Quantification of the flow cytometry results of ROS levels. Data are from three independent experiments and are presented as mean ± SD. Data are analyzed with one-way ANOVA followed by Tukey's multiple comparisons test. n.s., not significant; * $P < 0.05$; *** $P < 0.001$.

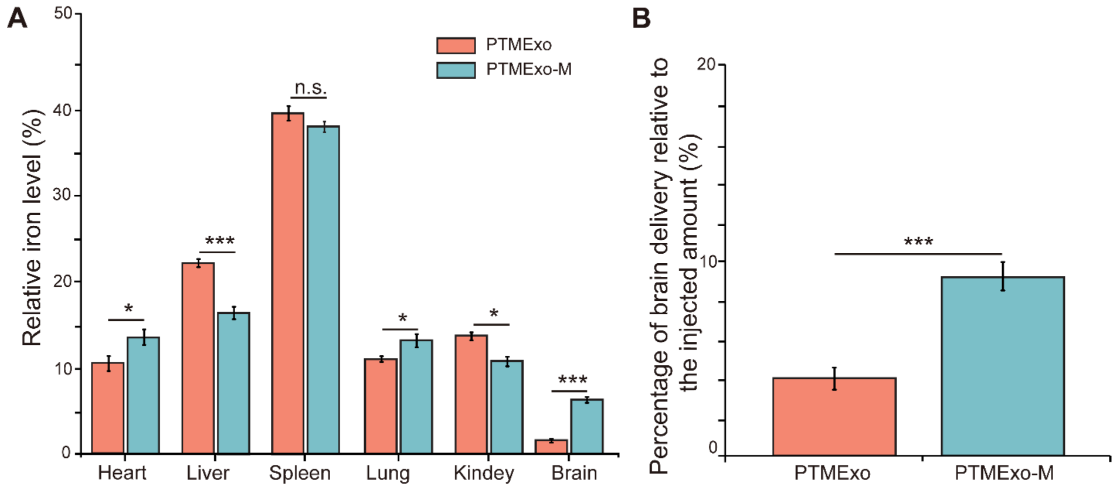


**Figure S22. Biodistribution of engineered exosomes (PNCAS-Tat-encapsulated MSCs-derived exosomes) in healthy mice 24 h after intravenous injection, with or without a magnet placed on the mice skulls.** The analysis was conducted by measuring the iron content (using ICP-OES) in each organ, subtracting the innate iron content (*i.e.,* the amount in an organ without exosome delivery) from the iron content with exosome delivery. "PTMExo" refers to PNCAS-Tat-encapsulated MSCs-derived exosomes. "-M" refers to using a magnet on the mice skulls. **(A)** Percentages of distribution of engineered exosomes in different major organs, relative to the total amount in these organs. **(B)** Percentage of engineered exosomes that had reached the brains, relative to the injected amount of engineered exosomes. Data are presented as mean ± SD (n = 5 mice) and analyzed with a two-tailed, unpaired t-test. n.s., not significant; * $P < 0.05$; *** $P < 0.001$.

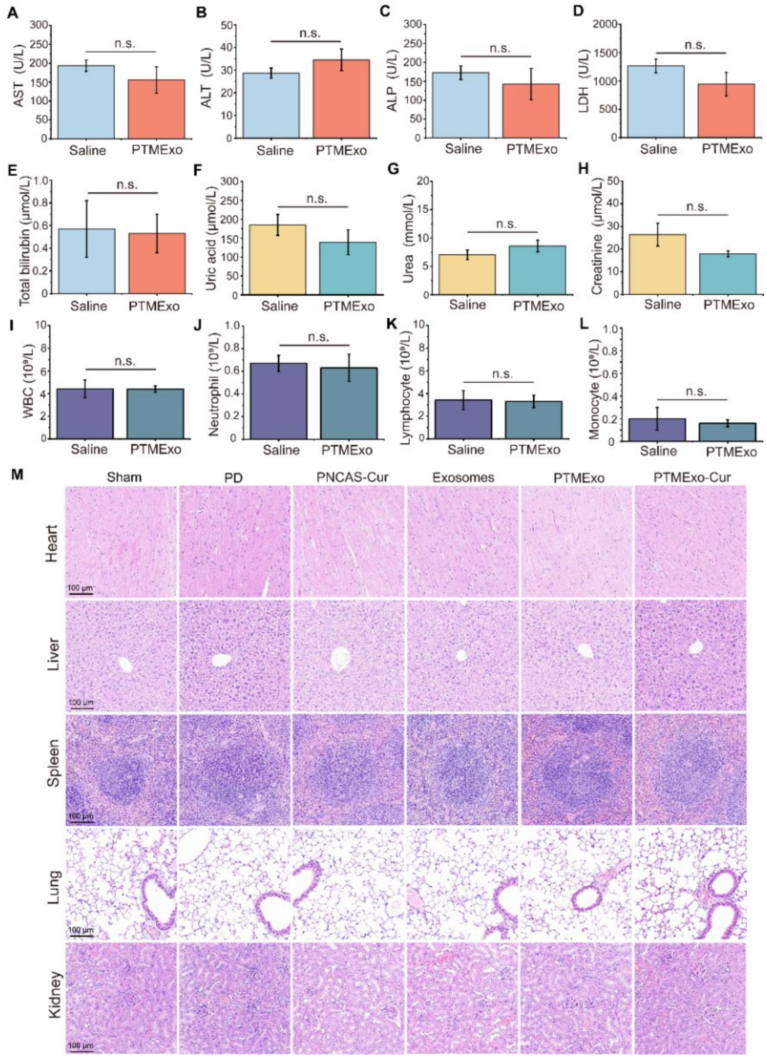


**Figure S23. Biocompatibility evaluation of engineered exosomes (PNCAS-Tat-encapsulated MSCs-derived exosomes).** PTMExo: PNCAS-Tat-encapsulated MSCs-derived exosomes. **(A-E)** Liver function analysis after an intravenous injection of high dose of engineered exosomes ($1\times10^{10}$ particles/mouse) in healthy mice. The evaluated biomarkers of liver function include aspartate aminotransferase, *i.e.,* AST, alanine aminotransferase, *i.e.,* ALT, alkaline phosphatase, *i.e.,* ALP, lactic dehydrogenase, *i.e.,* LDH, and total bilirubin. n = 4 mice (two males and two females). Data are presented as mean ± SD and analyzed with a two-tailed, unpaired t-test. n.s., not significant. **(F-H)** Kidney function analysis after an intravenous injection of high dose of engineered exosomes ($1\times10^{10}$ particles/mouse) in healthy mice. The evaluated biomarkers of kidney function include uric acid, urea, and creatinine. n = 4 mice (two males and two females). Data are presented as mean ± SD and analyzed with a two-tailed, unpaired t-test. n.s., not significant. **(I-L)** immunogenicity analysis after an intravenous injection of high dose of engineered exosomes ($1\times10^{10}$ particles/mouse) in healthy mice. The evaluated biomarkers of immunogenicity include white blood cell, neutrophil, lymphocyte, and monocyte. n = 4 mice (two males and two females). Data are presented as mean ± SD and analyzed with a two-tailed, unpaired t-test. n.s., not significant. **(M)** Tissue morphology analysis by H&E staining of heart, liver, spleen, lung, and kidney after 30 days of treatments of PD mice. The different mice groups include Sham (normal mice control with PBS treatment), PD (PD mice with PBS treatment), PNCAS-Cur (PD mice treated with CUR-loaded PNCAS-Tat), Exosomes (PD mice treated with MSCs-derived exosomes without PNCAS-Tat or CUR), PTMExo (PD mice treated with PNCAS-Tat-encapsulated MSCs-derived exosomes without CUR), and PTMExo-Cur (PD mice treated with CUR-loaded PNCAS-Tat-encapsulated MSCs-derived exosomes). n = 5 mice.

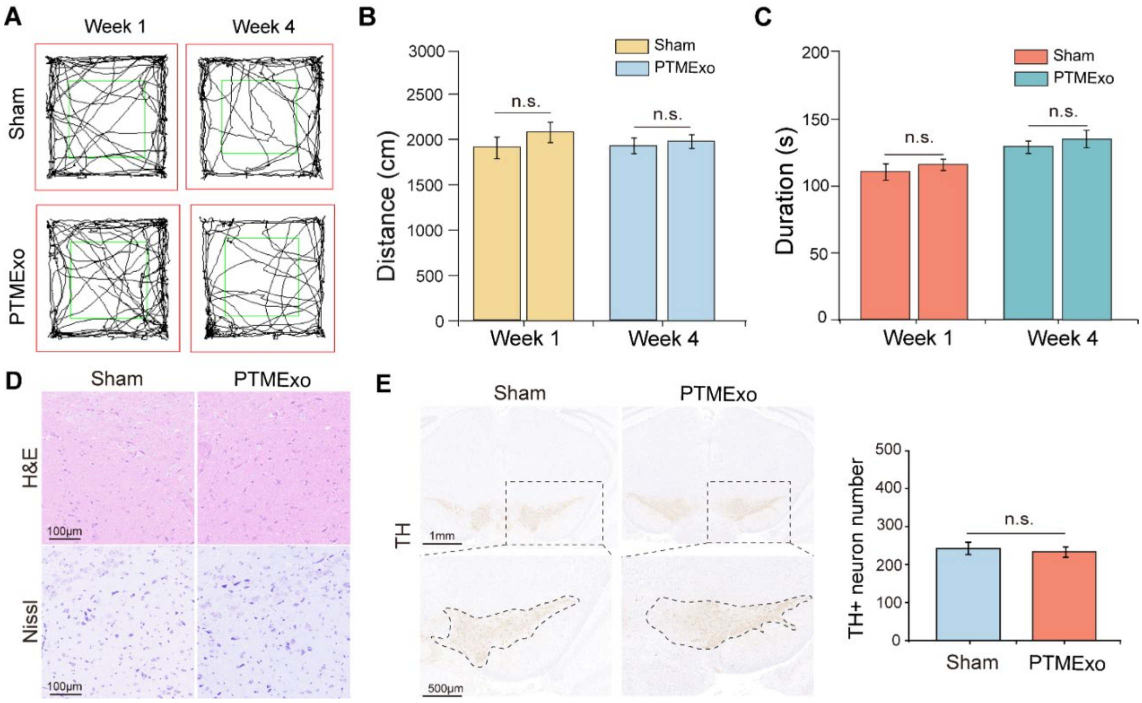


**Figure S24. Evaluation of neurotoxicity of engineered exosomes (PNCAS-Tat-encapsulated MSCs-derived exosomes) in healthy mice.** The evaluated mouse groups are as follows. Sham: healthy mice with PBS treatment. PTMExo: healthy mice treated with PNCAS-Tat-encapsulated MSCs-derived exosomes at an equivalent dose level as the therapeutic treatment ($1\times10^{10}$ particles/mouse). **(A)** Trajectories of mouse movement in the open-field experiment for behavior evaluation (motion capability). The evaluation was performed at week 1 and week 4. **(B)** Quantification (distance) of the results of open-field experiment. **(C)** Quantification (time duration) of the results of open-field experiment. **(D)** H&E staining and Nissl staining of brain tissue slices in mice substantia nigra region 30 days after injection of formulations. **(E)** TH immunohistochemistry of brain tissue slices in mice substantia nigra region 30 days after injection of formulations. Data are presented as mean ± SD (n = 5 mice) and analyzed with a two-tailed, unpaired t-test. n.s., not significant.

**Table S1. Comparison of the efficacy and cost of different exosome engineering methods.** It should be noted that most publications in this field did not report detailed quantitative analysis of the efficacy of loading. Here, representative publications with detailed quantitative analysis of loading efficacy are included. The list of publications is not exhaustive.

| | Name of methods | Efficacy | Cost | Comments |
|---|---|---|---|---|
| **Chemical engineering of exosomes** | Drug co-incubation with exosomes | ~2 × $10^5$ drug per vesicle | Low (because it involves only simple incubation) | Reference #20. Hydrophobic drugs are in the vesicle membrane, thus could destabilize the exosomes. |
| | Electroporation of exosomes | ~2 × $10^5$ drug per vesicle | High (because it involves the use of additional instrument that is difficult to scale up) | Reference #20. Hydrophobic drugs are in the vesicle membrane, thus could destabilize the exosomes. |
| | Loading drug to the source cells, to achieve drug loading to exosomes | ~3× $10^2$ drug per vesicle | Low (because it involves only simple incubation) | Reference #21. Hydrophobic drugs are in the vesicle membrane, thus could destabilize the exosomes. |
| | Our method | ~1×$10^6$ drug per vesicle | Low (because it involves only simple incubation) | Hydrophobic drugs are in the vesicle core, thus have no risk of destabilizing the exosomes. |
| **Genetic engineering of exosomes** | Transfection of source cells with Lipofectamine 2000 to send in plasmid DNAs, so as to load mRNAs in exosomes | 0.002-0.01 mRNA molecules in each exosome | Low (because it involves only simple incubation) | Reference #22. |
| | Electroporation | 0.002-0.01 | High | Reference #22. |

| | | | | |
|---|---|---|---|---|
| | of source cells to send in plasmid DNAs, so as to load mRNAs in exosomes | mRNA molecules in each exosome | (because it involves the use of additional instrument that is difficult to scale up) | |
| | Electroporation of exosomes to load mRNAs | 0.02-0.1 mRNA molecules in each exosome | High (because it involves the use of additional instrument that is difficult to scale up) | Reference #22. |
| | Cellular nanoporation to load mRNAs in exosomes: nano-electroporation of source cells to send in plasmid DNAs | 2-10 mRNA molecules in each exosome | High (because it involves the use of additional instrument that is difficult to scale up) | Reference #22; and *Nat. Biomed. Eng.* **2020,** *4,* 9–11. |

**Video S1. Tracking of the movement of a PNCAS-encapsulated vesicle that was probably exiting the source cell (BMSC).** An arrow is drawn to indicate the PNCAS-encapsulated vesicle. The PNCAS contained green QDs, and the vesicle was stained by a red vesicle dye DiO. Colocalization between green QDs and red vesicle dye DiO throughout the frames of the video suggests that it is a PNCAS-encapsulated vesicle. The video was captured at a frame rate 60 frames/hour. In the video, this PNCAS-encapsulated vesicle was initially stationary (probably bound to the cell membrane), and then suddenly showed rapid movement (probably diffusing after being released to the extracellular environment).

**Note S1. Calculation of the maximum number of curcumin (CUR) molecules loaded in each PNCAS and in each exosome, respectively.**

A sample of CUR-loaded PNCAS with maximum CUR loading (52.63% drug loading) was prepared. The sample was dispersed in water. The dry weight and particle number were determined by a balance and NTA, respectively. Only a portion of the original sample was used for NTA measurement (because in a NTA experiment a small sample volume is needed). The calculation below is conducted for this portion of the sample.

The dry weight is 675.50 μg. The particle number of PNCAS is $8.48\times10^{11}$. Thus, the mass of each CUR-loaded PNCAS is:

$$\frac{\text{Total mass of PNCAS (with CUR loading)}}{\text{Particle number of PNCAS}}=\frac{675.50\ \mu\text{g}}{8.48\times10^{11}}=7.97\times10^{-10}\ \mu\text{g}$$

The mass of CUR molecules in each PNCAS (with CUR loading) is:

$$\text{Mass of each PNCAS (with CUR loading)} \times \text{Drug Loading (\%)} = 7.97\times10^{-10}\ \mu\text{g} \times 52.63\% = 4.19\times10^{-10}\ \mu\text{g}$$

Therefore, the number of CUR molecules in each PNCAS (with CUR loading) is:

$$\frac{\text{Mass of CUR in each PNCAS (with CUR loading)}}{\text{Molecular weight of CUR}} \times 6.02\times10^{23}= \frac{4.19\times10^{-10}\ \mu\text{g}}{368.38\text{g/mol}} \times 6.02\times10^{23}/\text{mol}=6.85 \times 10^{5}$$

Because each PNCAS-encapsulated exosome contains on average ~1.5 PNCAS (Fig. 3H), the maximum number of CUR molecules loaded in each exosome is:

$$6.85 \times 10^{5} \times 1.5 = 1.0275 \times 10^{6}$$

**Note S2. A simplistic calculation to compare the scalability of conventional design of magnet setup (SEMS, *i.e.,* Static External Magnetic Separation) *vs.* novel design of magnet setup (MIMS, *i.e.,* Mobile Internal Magnetic Separation).**

Consider a PNCAS as the representative object (called "the Object" from this point on) to be magnetically isolated. The governing equation for calculating its isolation time is derived from the force balance between magnetic attraction force and viscous drag force, which are the main forces acted upon the Object during the magnetic separation process.

The magnetic attraction force (a force between the magnet and the Object) is given by

$$\mathrm{F_{mag}} = \mu_0 \cdot V \cdot M_p \cdot \nabla H$$

Where:

$\mathrm{F_{mag}}$: Magnetic attraction force (N).

$\mu_0$ : Permeability of free space ( $4\pi \times 10^{-7}$ H/m ).

$M_p$ : Saturation magnetization.

*V*: Volume of the Object ( $\mathrm{V} = \frac{4}{3}\pi \mathrm{r}^3$ , where r is the radius of the Object in meters)

$\nabla H$ : Magnetic field gradient (A/m$^2$).

The viscous drag force on the Object moving through a fluid (resistance to the magnetic isolation) is given by

$$\mathrm{F_{drag}} = 6\pi\eta r v$$

Where:

$\mathrm{F_{drag}}$: Viscous drag force (N).

$\eta$ : Dynamic viscosity of the fluid (Pa·s or N·s/m$^2$).

$r$ : Radius of the Object (m).

$v$ : Velocity of the Object relative to the fluid (m/s).

At equilibrium, the magnetic attraction force balances the viscous drag force:

$$\mathrm{F_{mag}} = \mathrm{F_{drag}}$$

Thus,

$$v = \frac{\mu_0 \cdot V \cdot M_p \cdot \nabla H}{6\pi\eta r} = \frac{2}{9\eta} \cdot r^2 \mu_0 \cdot \mathrm{M}_p \cdot \nabla H$$

The values of the parameters are as follows:

$\eta = 1.005\times10^{-3}\,\mathrm{N\cdot s}/m^2, r = 5\times10^{-8}m, \mu_0 = 4\pi\times10^{-7}\,\mathrm{H}/m$

The saturation magnetization of the Object (PNCAS) was measured by SQUID to be:

$M_p = 39.075\mathrm{emu/g}$

To change the unit of saturation magnetization from emu/g to emu/cm$^3$, the density value of the Object is needed, which is calculated as follows:

For 0.4 mg (dry mass) of PNCAS, there are ~$2\times10^{12}$ particles of PNCAS as measured by NTA. Thus, the density of PNCAS is:

$\rho = \frac{\mathrm{m}}{nV} = \frac{3\mathrm{m}}{4n\pi r^3} = 3800kg/m^3, (r = 5\times10^{-8}m, n = 2\times10^{12})$

$M_p = 39.075\times3800 = 1.48\times10^5 emu/cm^3$

For the value of the magnetic field gradient $\nabla H$, as a sample calculation, we set

$$\nabla H \approx 1.38\times10^7\,\mathrm{A/m^2}$$

This gives

$$v \approx 1.42\times10^{-6}\,\mathrm{m/s}$$

The time for the Object to travel a distance is given by

$$t = \frac{d}{v}$$

As a sample calculation, we set the distance value to be 0.025 m; this gives rise to the isolation time:

$$t \approx 17600\mathrm{s} \approx 4.89\mathrm{h}$$

As shown in the following schematic illustration, as the scale of the container goes up, in the SEMS design (the conventional design), the distance for the Object to travel goes up, while in the MIMS design (the novel design), the distance for the Object to travel stays virtually the same. Here, the location of the Object is set to represent the goal of near-complete separation.

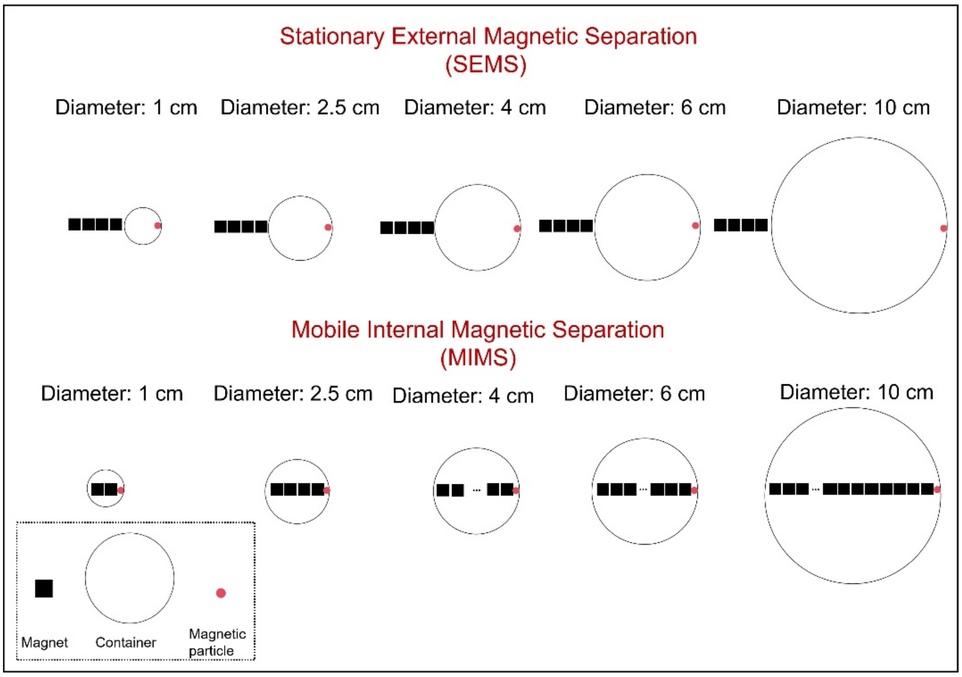


Using the above equations, the calculated separation times are shown in the following table, comparing SEMS *vs.* MIMS. Note that as the distance goes up, the value of magnetic field gradient $\nabla H$ goes down drastically.

| **SEMS (Stationary external magnetic separation)** | | | | |
|---|---|---|---|---|
| **Scale (cm)** | $\nabla$H **(T/m)** | $\nabla$H **(A/m²)** | $v$ **(m/s)** | t($h$) |
| 1 | 43.63 | $3.47\times10^{7}$ | $3.57\times10^{-6}$ | 0.78 |
| 2.5 | 17.39 | $1.38\times10^{7}$ | $1.42\times10^{-6}$ | 4.89 |
| 4 | 10.89 | $8.67\times10^{6}$ | $8.93\times10^{-7}$ | 12.5 |
| 6 | 7.27 | $5.79\times10^{6}$ | $5.96\times10^{-7}$ | 27.97 |
| 10 | 4.37 | $3.48\times10^{6}$ | $3.58\times10^{-7}$ | 77.78 |
| **MIMS (Mobile internal magnetic separation)** | | | | |
| **Scale (cm)** | $\nabla$H **(T/m)** **(from tip:1 mm)** | $\nabla$H **(A/m²)** **(from tip:1 mm)** | $v$ **(m/s)** | t($h$) |
| 1 | 173.9 | $1.38\times10^{8}$ | $1.42\times10^{-5}$ | 0.02 |
| 2.5 | 173.9 | $1.38\times10^{8}$ | $1.42\times10^{-5}$ | 0.02 |
| 4 | 173.9 | $1.38\times10^{8}$ | $1.42\times10^{-5}$ | 0.02 |
| 6 | 173.9 | $1.38\times10^{8}$ | $1.42\times10^{-5}$ | 0.02 |
| 10 | 173.9 | $1.38\times10^{8}$ | $1.42\times10^{-5}$ | 0.02 |

These results are also shown in the following figure.

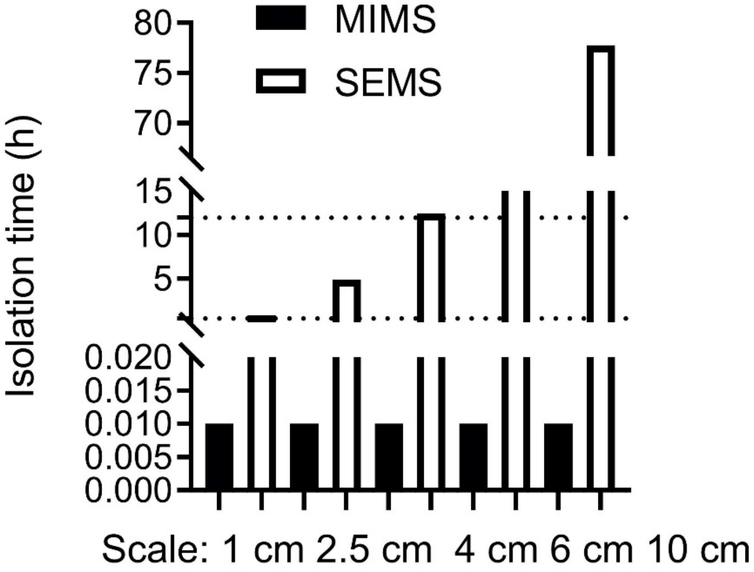


In these calculation results, for cylindrical containers with different diameters (1 cm, 2.5 cm, 4 cm, 6 cm, 10 cm), the separation times of the Object by SEMS are 0.78 h, 4.89 h, 12.5 h, 27.97 h (that is, over 1 day), and 77.78 h (that is, over 3 days), respectively. In contrast, the separation times of the Object by MIMS for the containers with the above different diameters can consistently reach 0.02 h (calculated at a separation distance of 1 mm).

To summarize, with the SEMS design, as the scale (diameter of the container) goes up, the travel distance goes up, and the magnetic field gradient goes down drastically, thereby leading to greatly increased separation time; in contrast, with the MIMS design, as the scale (diameter of the container) goes up, both the travel distance and magnetic field gradient remain the same, thereby leading to identical separation time.
