# Supplementary figures and images for: A Nanoparticle‐Integrated Complete Manufacturing Pipeline of Chemically Engineered Exosomes
Source: Adv Sci (Weinh). 2026 Mar 24;13(32):e16075. doi: 10.1002/advs.202516075 (PMC13252649; doi:10.1002/advs.202516075)

## Slide 1
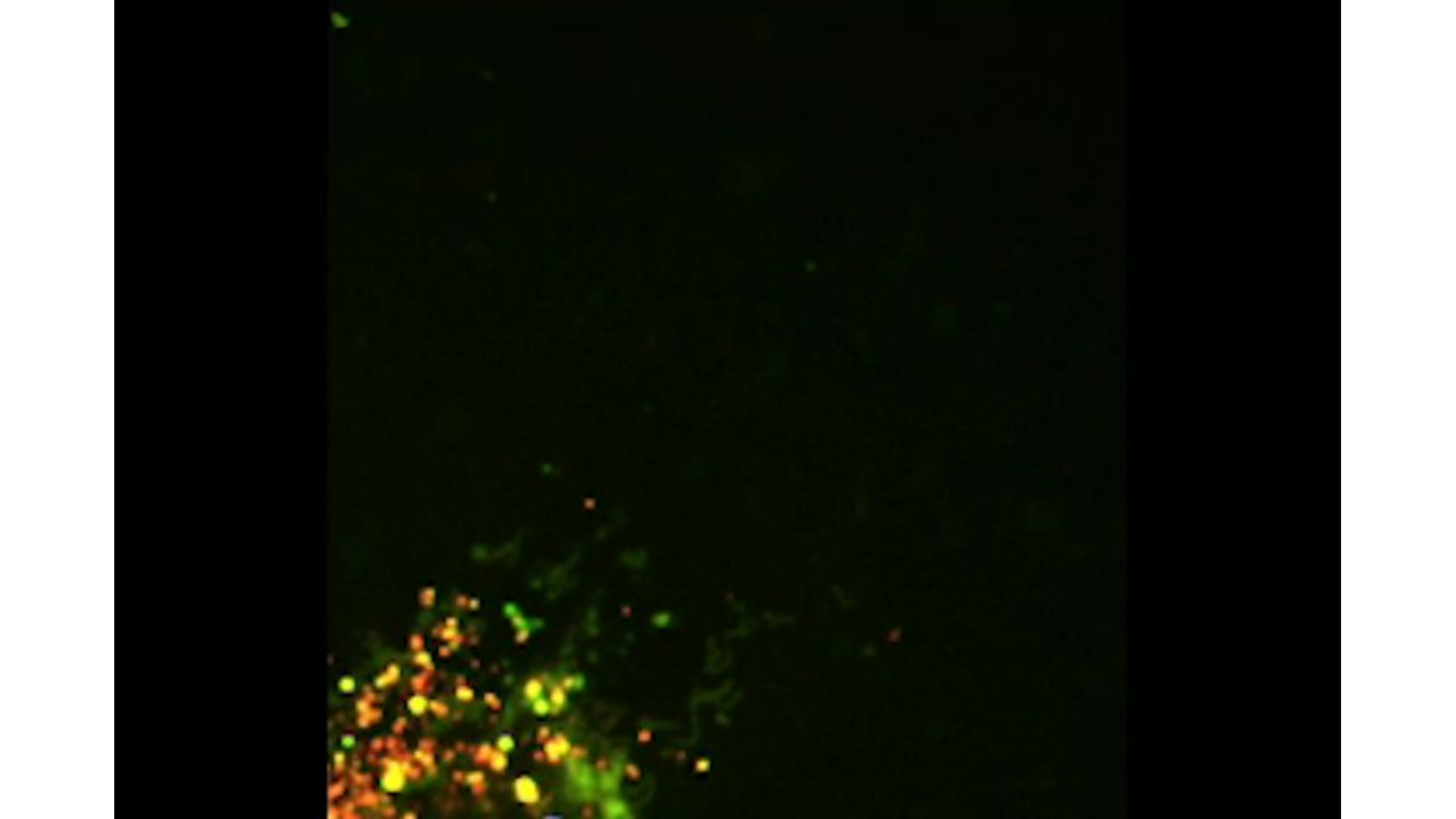

#

Supplement: Supplementary file 2 — Supporting File 2: advs74394‐sup‐0002‐Video1.pptx. [file ADVS-13-e16075-s001.pptx]
